# Physicochemical Properties, Equilibrium Adsorption Performance, Manufacturability, and Stability of TIFSIX-3-Ni for Direct Air Capture of CO_2_

**DOI:** 10.1021/acs.energyfuels.4c01368

**Published:** 2024-06-18

**Authors:** May-Yin Ashlyn Low, David Danaci, Hassan Azzan, Amanda Lim Jiayi, Gordon Wu Shun Yong, Ioanna Itskou, Camille Petit

**Affiliations:** † Department of Chemical Engineering, Imperial College London, London SW7 2AZ, United Kingdom; ‡ The Sargent Centre for Process Systems Engineering, Imperial College London, London SW7 2AZ, United Kingdom; § I-X Centre for AI in Science, Imperial College London, London W12 0BZ, United Kingdom

## Abstract

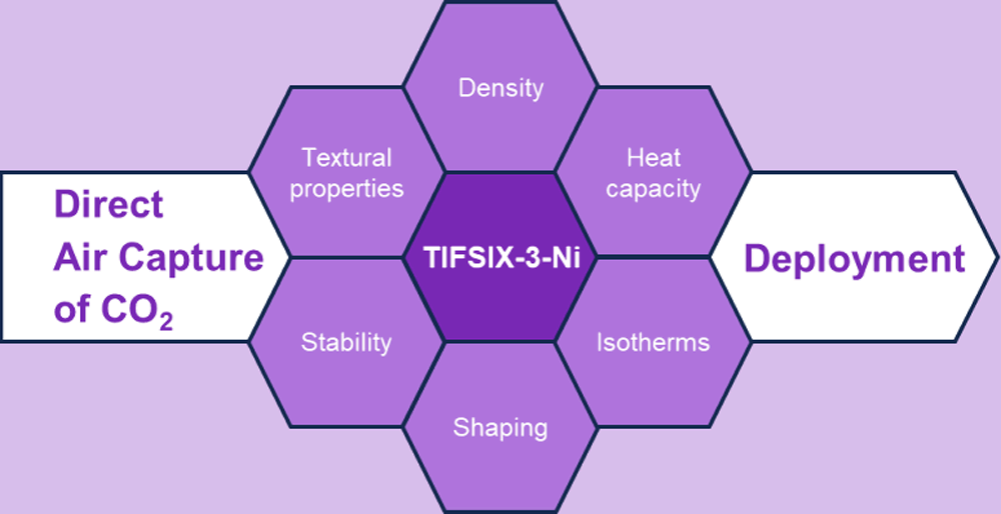

The use of adsorbents for direct air capture (DAC) of CO_2_ is regarded as a promising and essential carbon dioxide removal technology to help meet the goals outlined by the 2015 Paris Agreement. A class of adsorbents that has gained significant attention for this application is ultramicroporous metal organic frameworks (MOFs). However, the necessary data needed to facilitate process scale evaluation of these materials is not currently available. Here, we investigate TIFSIX-3-Ni, a previously reported ultramicroporous MOF for DAC, and measure several physicochemical and equilibrium adsorption properties. We report its crystal structure, textural properties, thermal stability, specific heat capacity, CO_2_, N_2_, and H_2_O equilibrium adsorption isotherms at multiple temperatures, and Ar and O_2_ isotherms at a single temperature. For CO_2_, N_2_, and H_2_O, we also report isotherm model fitting parameters and calculate heats of adsorption. We assess the manufacturability and process stability of TIFSIX-3-Ni by investigating the impact of batch reproducibility, binderless pelletization, humidity, and adsorption–desorption cycling (50 cycles) on its crystal structure, textural properties, and CO_2_ adsorption. For pelletized TIFSIX-3-Ni, we also report its skeletal, pellet, and bed density, total pore volume, and pellet porosity. Overall, our data enable initial process modeling and optimization studies to evaluate TIFSIX-3-Ni for DAC at the process scale. They also highlight the possibility to pelletize TIFSIX-3-Ni and the limited stability of the MOF under humid and oxidative conditions as well as upon multiple adsorption–desorption cycles.

## 1 Introduction

Direct air capture (DAC) of CO_2_ from the atmosphere using adsorbents is regarded as a promising and necessary technology to help limit global temperature rise to below 2 °C, a commitment made by 196 countries through the 2015 Paris Agreement. It is a challenge to isolate ultradilute concentrations of CO_2_ from the air (∼0.04%_vol_ or 0.4 mbar) under varying temperature and relative humidity conditions, but DAC allows for the quantitative measurement of captured CO_2_ and facilitates a pathway for long-term carbon storage.

Since 2016, over 200 adsorbents have been reported for DAC in the literature.^1^ However, to properly assess these adsorbents at scale, adsorbent screening should be conducted using key performance indicators determined from process modeling and optimization (*i.e.*, purity, recovery, productivity, energy consumption).^2−4^ To facilitate this process scale evaluation, various adsorbent properties are required such as equilibrium isotherms of all relevant species, heat and mass transfer coefficients, porosity, density (*i.e.*, skeletal, pellet, bed), heat capacity, and heat of adsorption.^1^

In a recent work, we investigated amine-functionalized polymeric resins as a promising class of adsorbents for DAC.^5^ Many of these are already commercially available, thereby facilitating the deployment of adsorption-based DAC processes. We experimentally measured and reported as many of the adsorbent properties listed above as possible for Purolite A110, and compared them to those of Lewatit VP OC 1065, a current benchmark adsorbent for DAC. The data for Purolite A110 and Lewatit VP OC 1065 can now be employed for DAC process modeling. Sufficient data is also available for zeolite 13X, however, these are scattered across different reports.^6−8^

Another class of adsorbent which has received a lot of attention for DAC are metal organic frameworks (MOFs), with over 30% of the recent DAC adsorbent literature focused on these materials.^1^ Among these, ultramicroporous MOFs, *i.e.*, MOFs with pores smaller than 0.7 nm,^9^ are often highlighted. Their small pore sizes and chemical nature allow for high CO_2_ adsorption capacity at the relevant low pressures (>1.0 mmol g^–1^ at 0.4 mbar CO_2_ and 25 °C) and limited N_2_ adsorption, as well as lower CO_2_ heat of adsorption than many amine-functionalized chemisorbents. Notable ultramicroporous MOFs reported for DAC include NbOFFIVE-1-Ni, SIFSIX-3-Cu, SIFSIX-18-Ni-β, TIFSIX-3-Ni, TIFSIX-3-Co, GeFSIX-3-Ni, WO_2_F_4_-1-Ni, and FJI-H38.^9−16^ We direct the reader to Zhang et al.^17^ and Bose et al.^18^ for a comprehensive overview of these, and other MOF adsorbents for DAC.

Among ultramicroporous MOFs, TIFSIX-3-Ni is a particularly promising candidate adsorbent for DAC. It is formed of a grid-like framework of Ni nodes joined together by pyrazine organic linkers and TiF_6_^–^ anion pillars, with a pore size of 0.325 nm,^19^ defined as the diagonal F–F distance across a channel.^18^ TIFSIX-3-Ni was first studied for DAC in 2017 by Kumar et al.^9^ They reported a high CO_2_ adsorption capacity of 1.1 mmol g^–1^ at 0.4 mbar CO_2_ partial pressure and 25 °C, a moderate CO_2_ heat of adsorption of 50 kJ mol^–1^, and minimal loss in BET area and CO_2_ adsorption performance after exposure to 75% relative humidity (RH) at 40 °C for 14 days. Volumetric CO_2_ adsorption isotherms measured at 0, 10, and 25 °C, and a N_2_ adsorption isotherm measured at 25 °C were also reported, though no isotherm model fitting was conducted. Mukherjee et al.^12^ also reported a gravimetric H_2_O adsorption isotherm measurement at 25 °C. In addition, they conducted dynamic column breakthrough (DCB) measurements for TIFSIX-3-Ni using both dry and humid CO_2_ at 1 mbar and 3 mbar balanced with N_2_, where they observed a significant decrease in CO_2_ adsorption capacity in the presence of humidity. However, Ullah et al.^19^ reported slow H_2_O kinetics in comparison to CO_2_ from their DCB experiments and that shortened adsorption cycles would allow for ∼90% retention of the dry CO_2_ adsorption capacity. They further elucidated the CO_2_–H_2_O coadsorption mechanisms using *in situ* spectroscopy and molecular simulations, proposing that CO_2_ and H_2_O can occupy the same ultramicropore at low water loadings, up to a maximum of one CO_2_ molecule and three H_2_O molecules. At higher water loadings, however, an energetically favored water network will form with four H_2_O molecules in one ultramicropore and displace CO_2_.

TIFSIX-3-Ni also seems to be promising in terms of its manufacturability and scalability. Its synthesis is relatively straightforward and does not require the use of hydrofluoric acid as a precursor, unlike other ultramicroporous MOFs such as NbOFFIVE-1-Ni.^10^ A disclosure by the Commonwealth Scientific and Industrial Research Organisation (CSIRO) has documented the synthesis of up to 65 g of powdered TIFSIX-3-Ni to be used in a MOF-polymer nanocomposite coating for application in a DAC adsorption unit.^20^ CSIRO termed this nanocomposite technology “Airthena” and conducted a pilot-scale demonstration of their DAC unit where they captured 8 kg of CO_2_ from ambient air over 2680 cycles.^20,21^ The company MOF Technologies (now known as Nuada) previously sold TIFSIX-3-Ni in both powder and shaped form, though no further details are currently available.^22,23^ Kearns et al.^24^ produced 3D printed monoliths of TIFSIX-3-Ni mixed with graphite and bentonite, and also proposed an improved synthesis of TIFSIX-3-Ni powder using a lower-cost nickel hydroxide precursor instead of anhydrous nickel carbonate. Warfsmann et al.^25^ had also previously reported a thin-film synthesis of TIFSIX-3-Ni.

Despite many promising studies on TIFSIX-3-Ni, this MOF has not yet been widely considered in DAC process modeling and optimization studies. This is due to the unavailability of key properties for TIFSIX-3-Ni such as its density, porosity, specific heat capacity, and isotherm model fitting parameters for CO_2_, N_2_, and H_2_O. Isotherms for other gases present in air which may compete with CO_2_, such as O_2_ and Ar, have also not been measured. Besides, the previous studies highlighted above present some discrepancies regarding the effect of moisture on TIFSIX-3-Ni stability and performance.

Therefore, in this study, we aim to report as many of the material and equilibrium adsorption properties for TIFSIX-3-Ni as possible, to facilitate process-scale evaluation of the adsorbent for DAC. These include: physical properties such as the skeletal, pellet, and bed density; thermal properties such as the specific heat capacity and thermal stability; textural properties such as micropore and total pore volume, BET area, and pellet porosity; and adsorption properties including Ar and O_2_ isotherms at a single temperature, and CO_2_, N_2_, and H_2_O isotherms at multiple temperatures. Where isotherms at multiple temperatures are measured, we determined isotherm model fitting parameters and heats of adsorption. We also assessed the practicality of using this adsorbent in a DAC process by studying its manufacturability in terms of reproducibility and binderless shaping, as well as investigating its stability to different process conditions such as humidity and cyclic adsorption.

## 2 Materials and Methods

### 2.1 Chemicals

Details of the chemicals used in this study are provided in Table S1. The purity of the gases used for the study were as follows; for gas adsorption measurements: CO_2_ (BOC, N5.0 grade), N_2_ (BOC, N6.0 grade), Ar (BOC, N5.0 grade), O_2_ (BOC, N5.0 grade). For pycnometry measurements: He (BOC, N5.0 grade). For thermogravimetric measurements: N_2_ (BOC, N4.8 grade). For differential scanning calorimetry (DSC) measurements: N_2_ (BOC, N5.2 grade). For *in situ* XRD measurements: CO_2_ (BOC, N4.5 grade), N_2_ (BOC, N4.8 grade).

### 2.2 Material Synthesis

#### 2.2.1 Synthesis of NiTiF_6_·6H_2_O

Nickel hexafluorotitanate hexahydrate crystals were synthesized following the procedure published by Wu at one-fourth scale.^26^ Specifically, 3.823 g of anhydrous nickel(II) carbonate was dispersed in a plastic beaker containing 10.678 g of deionized water while stirring. Then, 6.125 mL of dihydrogen hexafluorotitanate was added dropwise while the solution was stirring continuously. The solution was then filtered through a poly(ether sulfone) (PES) 0.45 μm syringe filter into a PTFE evaporating dish and left in the fume cupboard at room temperature to crystallize. Green hexagonal crystal pillars formed over 5 days which were then ground with a pestle and mortar and left to dry in the fume cupboard at room temperature for a further 2 days.

#### 2.2.2 Synthesis of TIFSIX-3-Ni

TIFSIX-3-Ni powder was synthesized following the procedure published by Kumar et al.^9^ First, 2.50 g of pyrazine was dissolved in 1.00 mL of deionized water in a 30 mL glass vial (solution was stirred at 1000 rpm for 30 min and then sonicated to fully dissolve pyrazine). Then, 500 mg of ground NiTiF_6_ crystals were added to the solution, which was left to stir at room temperature for 48 h with a closed lid. This yielded a blue powder precursor, which was air-dried for 2 h and then heated in an oven at 160 °C for 24 h, to yield approximately 500 mg of the final TIFSIX-3-Ni powder. The same synthesis was also successfully done at four times scale to yield approximately 2 g of final product per synthesis.

#### 2.2.3 Pelletization of TIFSIX-3-Ni Powder

Powdered TIFSIX-3-Ni (400 mg) was ground using a mortar and pestle and placed into a pellet die (13 mm evacuable stainless steel, Specac). This was positioned into a manual hydraulic press (Atlas Manual 15T, Specac) fitted with a low tonnage gauge conversion kit (0–1 t, Specac). A load of 0.5 t was applied and maintained for 45 s to form a pellet approximately 5 mm in height. The pellet was then ejected with a small load applied to an extractor ring placed onto the base of the pellet die.

### 2.3 Characterization of Chemical Features

Powder X-ray diffraction (XRD) was carried out using three different instruments depending on their availability. The first option was a PANalytical X’Pert Pro X-ray diffractometer with an anode voltage of 40 kV and an emission current of 20 mA using monochromatic Cu Kα radiation (λ = 1.54178 Å). The detector used was an X’Celerator silicon strip detector. Points were recorded over a 2θ angle range of 5 to 45° with a step size of 0.0167°. The second option was a PANalytical X’Pert Pro X-ray diffractometer with an anode voltage of 40 kV and an emission current of 40 mA using monochromatic Cu Kα radiation (λ = 1.54178 Å). The detector used was an X’Celerator silicon strip detector. Points were recorded over a 2θ angle range of 5 to 45 or 50° with a step size of 0.0167°. The third option was a Bruker 2D PHASE X-ray diffractometer with an anode voltage of 30 kV and an emission current of 10 mA using monochromatic Cu Kα radiation (λ = 1.54178 Å). The detector used was a LYNXEYE SSD160. Points were recorded over a 2θ angle range of 6 to 45° with a step size of 0.0161°.

In-situ XRD was performed using a PANalytical Empyrean X-ray diffractometer with an anode voltage of 40 kV and an emission current of 40 mA using monochromatic Cu Kα radiation (λ = 1.54178 Å). The detector used was a PIXcel^1D^ detector. Points were recorded over a 2θ angle range of 5 to 50° with a step size of 0.0131°. TIFSIX-3-Ni powder and pellet samples were subjected to the following procedure: 1) Sample exposed to ambient air. 2) Flow N_2_ at 2 mL min^–1^ for 10 min at room temperature. 3) Increase the temperature to 160 °C at a rate of 10 °C min^–1^ and hold for 10 min at 160 °C under the same N_2_ flow. 4) Cool down to room temperature under the same N_2_ flow. 5) Flow CO_2_ at 2 mL min^–1^ for 40 min at room temperature. An XRD pattern was measured at the end of steps 1, 2, 3, and 5.

X-ray photoelectron spectroscopy (XPS) analyses were performed using a Thermo Scientific K-Alpha X-ray photoelectron spectrometer equipped with an MXR3 Al Kα monochromatic X-ray source (*h*ν = 1486.6 eV) set to 72 W (6 mA and 12 kV). Powder samples were mounted onto carbon tape for measurement. The data was analyzed with Thermo Scientific Avantage software for C 1*s*, N 1*s*, O 1*s*, Ni 2*p*, Ti 2*p* and F 1*s* spectra. The adventitious carbon (C–C) peak set at 284.8 eV was used for binding energy calibration.

### 2.4 Characterization of Textural Properties and Density

N_2_ adsorption isotherms at −196 °C and CO_2_ adsorption isotherms at 0 °C were measured using a Micromeritics 3Flex porosity analyzer. Temperature control at 0 °C was achieved by filling the provided liquid nitrogen dewar with an ice bath. Samples were first degassed ex-situ using a Micromeritics VacPrep 061 at 0.02 mbar and 160 °C for 24 h, with the temperature increased from room temperature at a rate of 10 °C min^–1^, then degassed *in situ* at 120 °C and 0.00002 mbar for 24 h. Approximately 100 mg of dried samples were used for the measurements. The specific surface area from N_2_ adsorption was determined using the “BET surface identification” method (BETSI),^27^ while the specific surface area from CO_2_ adsorption was determined using the BET method^28^ with the Rouquerol criteria applied.^29^ The micropore volume was estimated using the Dubinin-Astakhov method for all measurements.^30,31^ The total pore volumes for powder samples were estimated from the N_2_ loading at a relative pressure (P/P_0_) of 0.97, using a molar volume of 28.775 mmol cm^–3^ for N_2_ at 1.00 atm and −196 °C.^32^ For pelletized samples, a pore size distribution in the pore width range 0.45 to 45 nm was determined using the density functional theory (DFT) model available in the 3Flex software, “N2 – Tarazona NLDFT, Esf = 30.0K”, with a cylinder pore geometry kernel and 0.20000 regularization.

Mercury intrusion porosimetry (MIP) was done on pelletized samples using a Micromeritics AutoPore IV Series Mercury Porosimeter. A 400 mg pellet was cut into 9 pieces following a grid pattern, yielding pieces of ∼4 mm in dimension, which were degassed ex-situ for at least 12 h at 0.02 mbar and 160 °C using a Micromeritics VacPrep 061, with the temperature increased from room temperature at a rate of 10 °C min^–1^. These were then added to a mercury penetrometer with a capillary intrusion stem volume of 1.1 cm^3^ and subjected to measurements in the pressure range of 3.7 × 10^–2^ bar to 2.23 × 10^3^ bar. The AutoPore IV software (version 9500) was used to obtain the pore size distribution in the pore width range 5 nm to 345 μm.

For pelletized samples, the total pore volume was estimated by combining the pore size distribution results from N_2_ adsorption isotherm and MIP measurements, based on the procedure described in our previous work.^33^ The two pore size distributions were combined by selecting a transition point and assuming continuity between both pore size distributions; 45 nm was chosen as the transition point as the N_2_ adsorption results would be more accurate in the micro to mesoporous range. The pellet density (ρ_pellet_) was also extracted from the MIP results. Assuming that cylindrical pellets with a height to diameter aspect ratio of 0.9 can be manufactured, a maximum random packing density of 0.72 can be achieved,^34^ or a bed voidage (ε_bed_) of 0.28. Using this and the total pore volume (*V*_total pores_) obtained from the method described above, additional adsorbent properties needed for process modeling such as the bed density (ρ_bed_), skeletal density (ρ_skeletal_), and porosity (ε_pellet_) of TIFSIX-3-Ni pellets can be calculated using eqs 1 to (3):
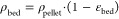
1
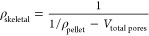
2
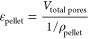
3

### 2.5 Characterization of Thermal Properties

Thermogravimetric analysis (TGA) was carried out using a Netzsch TG 209 F1 Libra thermal analyzer for TIFSIX-3-Ni powder samples. Approximately 20 mg of untreated sample was placed in an alumina crucible and subjected to a heat treatment from room temperature to 900 °C at a ramp rate of 10 °C min^–1^ under protective N_2_ flow (100 mL min^–1^). A correction run with an empty alumina crucible and identical analysis conditions was performed prior to these measurements to account for buoyancy effects.

The specific heat capacity was measured using a PerkinElmer DSC 8000 differential scanning calorimeter (DSC) following a methodology adapted from Moosavi et al.^35^ Approximately 5 mg of untreated TIFSIX-3-Ni powder sample was placed into an aluminum DSC pan and crimped with a corresponding aluminum lid with a manually pierced pinhole. The sample pan was placed into the sample chamber, while an identical empty aluminum pan and lid (uncrimped) was placed into the reference chamber. The external chiller was set to −20 °C, and the chambers were then subjected to the following temperature profile: isothermal at 20 °C for 5 min, heating ramp to 160 °C at a rate of 10 °C min^–1^, isothermal at 160 °C for 15 min, all under a N_2_ flow of 40 mL min^–1^. This profile was repeated without opening the sample and reference chambers until three overlapping heat flow curves were obtained. Prior to each series of measurements, an empty aluminum DSC pan and lid, and a sapphire standard, were used to obtain baseline and reference curves, respectively. The same procedure as outlined above was used for these measurements, though the aluminum pans were not crimped. Heat flow curves for all measurements are shown in Figure S1a. The specific heat capacity curves were then derived with the PerkinElmer DSC 8000 software using the last three overlapping heat flow curves for each component (baseline, reference, sample). This methodology was verified by measuring the heat capacity of the α-alumina (sapphire) disc provided by the DSC instrument manufacturer, where the experimental results were comparable to the literature values provided by the National Institute of Standards and Technology (NIST)^36^ (Figure S1b).

### 2.6 Analyses of Gas and Vapor Adsorption Properties

Gas and vapor adsorption isotherms were measured volumetrically using a Micromeritics 3Flex porosity analyzer for CO_2_ at 15, 25, 35, 60, 70, 80, and 120 °C, for N_2_ and H_2_O at 15, 25, and 35 °C, and for Ar and O_2_ at 25 °C. All experimental isotherm data are provided as AIF files^37^ and CSV files as Supporting Information. Samples were first degassed ex-situ using a Micromeritics VacPrep 061 at 0.02 mbar and 160 °C for 24 h, with the temperature increased from room temperature at a rate of 10 °C min^–1^, then degassed *in situ* at 120 °C and 0.00002 mbar for 24 h. A given aliquot of sample (∼100 mg after drying) was used to measure all isotherms for CO_2_, and a new aliquot used for all H_2_O isotherms. A final new aliquot of sample was used for the N_2_, Ar, and O_2_ measurements, which were done in the gas order listed. A glass stirring rod was also placed in each sample tube for all measurements to reduce the free space volume. For measurements at and below 35 °C, temperature control was achieved by submerging the sample tubes in a dewar containing a water-glycol based bath fluid. The bath temperature was measured with a glass thermometer and maintained using a Julabo F250 recirculating cooler. For measurements at and above 60 °C, temperature control was achieved by using the heating mantle provided with the 3Flex instrument.

Dual-site Langmuir (DSL) isotherm model fits for the CO_2_ adsorption data, single-site Langmuir (SSL) isotherm model fits for the N_2_ data, and “universal isotherm model”^38^ fits for the H_2_O adsorption data were performed using the procedure described in our previous works.^33,39,40^ This procedure was carried out with MATLAB R2020a (The Mathworks Inc.) using the in-house software package isothermFittingTool.^41^

The isosteric heats of adsorption for CO_2_, N_2_, and H_2_O were calculated using the van ’t Hoff equation for specified loading (*n*) and corresponding pressure (*P*) values. Isotherm data (*P* vs *n*) at each temperature (*T*) were first interpolated using smoothing spline interpolation in MATLAB. A set of points with 0.01 mmol g^–1^ intervals were generated over the loading range common to all temperatures, and the corresponding *P* values were determined using the smoothing spline interpolation. A linear regression was then applied to the ln(*P*) vs 1/*T* data for each value of *n* using the LINEST function in Microsoft Excel. The heat of adsorption for each value of *n* (*ΔH*_*n*_) was then calculated using eq 4, where the derivative corresponds to the slope of the linear regression and *R* is the universal gas constant.
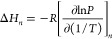
4

The limiting heats of adsorption (Δ*H*_0_) for CO_2_, N_2_, and H_2_O were calculated using the Henry constants determined for each isotherm temperature.^42^ Virial plots (*n* vs ln(*P*/*n*)) were created and the low loading data at each temperature was fitted to a linear equation. The negative exponent of the intercept gives the Henry constant (*K*). A linear regression can then be applied to ln*K* vs 1/*T*, from which the slope can be used to calculate Δ*H*_0_ as shown in eq 5.
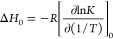
5

### 2.7 Effect of Humidity Studies

#### 2.7.1 Exposure to H_2_O Only

H_2_O adsorption isotherms were measured volumetrically using a Micromeritics 3Flex porosity analyzer at 25 °C up to 15% RH, 48% RH, and 77% RH, respectively, following the procedure in Section 2.6. These samples were then removed from the 3Flex, where ∼5 mg were used for XRD measurements following the procedure in Section 2.3. N_2_ isotherms at −196 °C and CO_2_ isotherms at 25 °C were measured on the remaining ∼95 mg samples following the procedures in Section 2.4 and 2.6, respectively.

#### 2.7.2 Exposure to O_2_ and H_2_O (Closed Environment)

Samples were subjected to a closed environment of 75% RH and 90% RH at 40 °C for 14 days following a procedure adapted from Kumar et al.^9^ Two 250 mL beakers were filled with separate saturated salt solutions of sodium chloride and potassium nitrate to produce RH levels of 75% and 90%, respectively.^43^ Each beaker was placed in its own 1 L Kilner clip top mason jar with a rubber seal, along with ∼100 mg of sample placed in an open 10 mL glass vial and a digital hygrometer-thermometer (Figure S2). Both mason jars were then placed in an oven set to 40 °C for 14 days. After this time, the samples were removed and XRD patterns were immediately measured using ∼5 mg of these wet samples following the procedure in Section 2.3. N_2_ isotherms at −196 °C and CO_2_ isotherms at 25 °C were then measured on the remaining ∼95 mg samples following the procedures in Section 2.4 and 2.6, respectively.

#### 2.7.3 Exposure to O_2_ and H_2_O (Atmospheric Exposure)

Approximately 100 mg of sample was placed in an open 30 mL glass vial and left on a shelf in the laboratory for 6 months. The average temperature and RH level in the lab is approximately 22 °C and 45% RH, respectively. After this time, XRD was performed on ∼5 mg of sample following the procedure in Section 2.3, and N_2_ isotherms at −196 °C and CO_2_ isotherms at 25 °C were measured on the remaining ∼95 mg samples following the procedures in Section 2.4 and 2.6, respectively.

### 2.8 Cycling Studies

A ∼4 mm pellet piece of TIFSIX-3-Ni (obtained from the procedure described in Section 2.4) was cut into granules of approximately 1 to 2 mm in diameter. Twenty mg of these granules were subjected to multiple adsorption–desorption cycles in ambient air (CO_2_ concentration ∼400 ppm) using a Netzsch TG 209 F1 Libra thermal analyzer. The sample was first subjected to a “long cycle” to achieve gas adsorption saturation as follows: sample heated to 160 °C from 30 °C at a rate of 5 °C min^–1^, held at 160 °C for 6 h, cooled to 30 °C at a rate of 5 °C min^–1^, held at 30 °C for 14 h, heated to 160 °C at a rate of 5 °C min^–1^, and held at 160 °C for 6 h, all under a mixture of ambient air (190 mL min^–1^) and protective He flow (10 mL min^–1^). The same sample was then subjected to 50 “short cycles” as follows: sample initially heated to 160 °C from 30 °C at a rate of 5 °C min^–1^, held at 160 °C for 6 h, cooled to 30 °C at a rate of 5 °C min^–1^, held at 30 °C for 1 h, heated to 160 °C at a rate of 5 °C min^–1^, held at 160 °C for 15 min, and the final cooling and heating steps repeated another 49 times, with all steps carried out under a mixture of ambient air (190 mL min^–1^) and protective He flow (10 mL min^–1^). After the series of “short cycles”, the same sample was subjected to another “long cycle” exactly as described previously. We recorded the relative humidity of the ambient air over the course of each measurement using a Lascar EL-SIE-2 temperature and humidity data logger.

Before each cycling measurement, the sample was degassed ex-situ for at least 12 h at 0.02 mbar and 160 °C using a Micromeritics VacPrep 061, with the temperature increased from room temperature at a rate of 10 °C min^–1^. A correction run with an empty crucible and identical analysis conditions was also performed before each measurement to account for buoyancy effects. An aluminum crucible with 0.2 mm holes drilled at 0.5 mm pitch and placed in a perforated stainless-steel holder (Figure S3) was used for these measurements to ensure optimal gas flow through the sample.

The postcyclic sample was characterized using XRD, N_2_ adsorption at −196 °C, and CO_2_ adsorption at 25 °C. For XRD measurements, the postcyclic TIFSIX-3-Ni sample was crushed into powder as the granules did not have a sufficient surface width for the beam. For isotherm measurements, glass beads of 2 mm diameter were used in addition to a glass rod to fill the remaining volume of the 3Flex tubes to minimize measurement error as only ∼20 mg of the postcyclic sample was available. The same procedure and amount of sample were also used to characterize and compare as-synthesized TIFSIX-3-Ni pellets.

## 3 Results and Discussion

### 3.1 Material Properties

**Figure 1:**
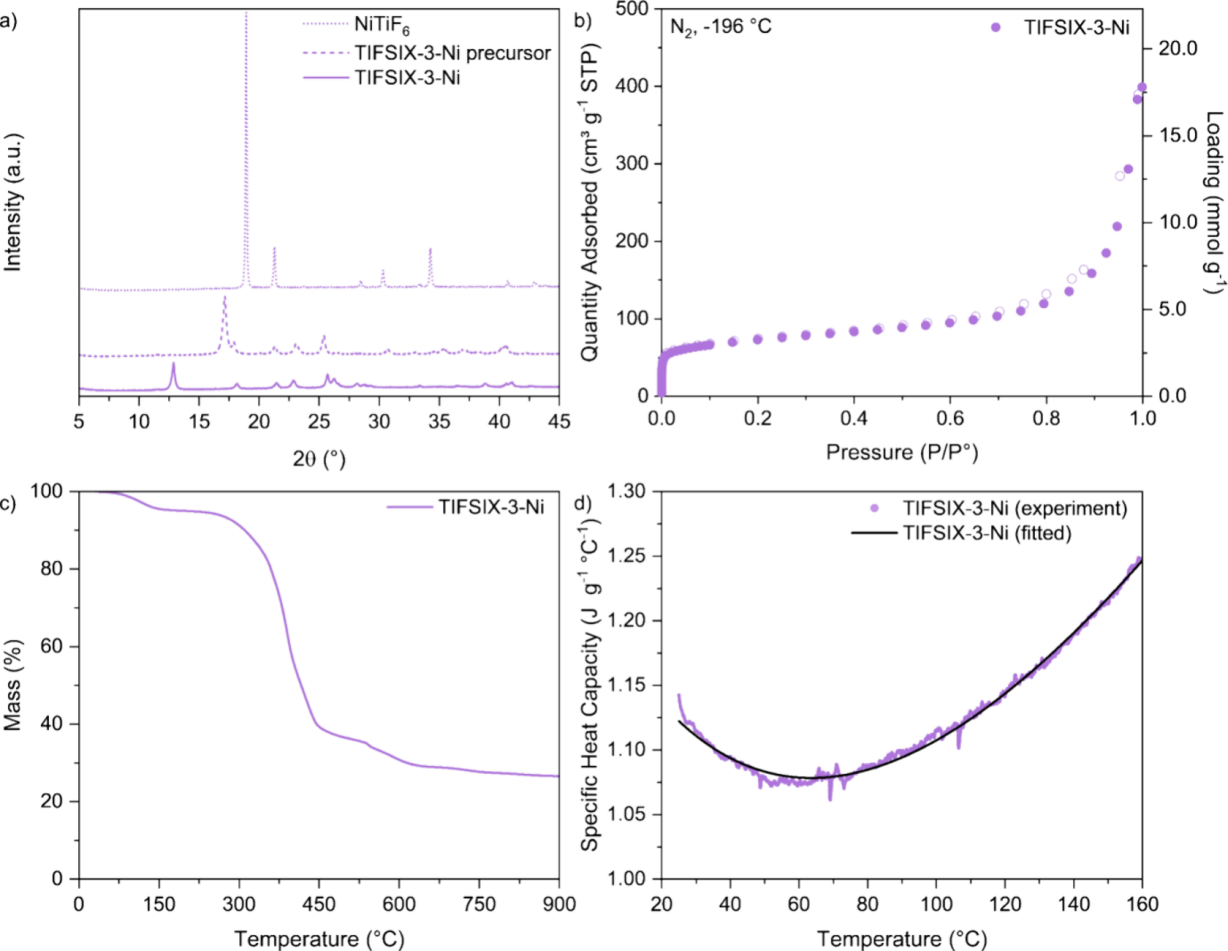
(a) XRD patterns of NiTiF_6_, TIFSIX-3-Ni precursor, and final TIFSIX-3-Ni. (b) N_2_ adsorption (filled symbols) and desorption (open symbols) isotherms at −196 °C, (c) TG curve under N_2_ atmosphere up to 900 °C, and (d) specific heat capacity as a function of temperature for TIFSIX-3-Ni.

The XRD patterns of ground NiTiF_6_ crystals, TIFSIX-3-Ni precursor powder, and the final TIFSIX-3-Ni powder (Figure 1a) match those reported in the literature^9,44^ (Figure S4) and suggest the successful synthesis of the final TIFSIX-3-Ni sample. Images of these compounds are shown in Figure S5. We then used N_2_ adsorption at −196 °C to assess its porosity (Figure 1b), revealing a total pore volume of 0.50 cm^3^ g^–1^, a micropore volume of 0.11 cm^3^ g^–1^, and a BET area of 264 m^2^ g^–1^.

To determine the thermal stability of TIFSIX-3-Ni, which influences the maximum temperature that can be used for regeneration of this material in a DAC process, we conducted TGA with a N_2_ flow up to 900 °C. From Figure 1c, we see that water desorbs from the sample up to 150 °C, after which there is no further mass loss until 230 °C, when the crystal structure begins to degrade. The results correspond with those reported by Kumar et al., who reported a thermal stability up to 280 °C in a N_2_ atmosphere.^9^

We also used DSC to determine the specific heat capacity of TIFSIX-3-Ni between 25 to 160 °C (Figure 1d), *i.e.*, a range of temperatures relevant to a DAC process. The specific heat capacity relates to the energy input required for sensible heating of the adsorbent during regeneration, where a higher specific heat capacity indicates that the adsorbent requires more energy to be heated to a given temperature. This property is a necessary input in process modeling and was not previously reported for TIFSIX-3-Ni. Following the methodology discussed in Section 2.5, we determined the specific heat capacity of TIFSIX-3-Ni to range from 1.06 to 1.25 J g^–1^ °C^1–^. This is comparable to the specific heat capacity reported for SIFSIX-3-Ni (1.07 J g^–1^ °C ^–1^),^45^ which has the same crystal structure as TIFSIX-3-Ni with Si atoms in place of Ti.

We can model our experimental data using eq 6:^46^
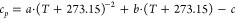
6where *c*_*p*_ is the specific heat capacity, *T* is the temperature (in °C), and *a*, *b*, and *c* are fitted parameters with values and uncertainty bounds of 1.077 ± 0.004 × 10^5^ J °C g^–1^, 5.65 ± 0.02 × 10^–3^ J g^–1^ °C^2–^, and 1.773 ± 0.008 J g^–1^ °C^1–^, respectively. The increase in *c*_*p*_ as the temperature decreases below approximately 60 °C is opposite to the expected trend. This “anomaly” has been observed in ZIF-8 and was associated with flexibility in its crystal structure due to linker rotation.^47^ The possibility of flexibility in the crystal structure of TIFSIX-3-Ni is further discussed in Section 3.3.2.

### 3.2 Adsorption Properties

#### 3.2.1 CO_2_ Adsorption

**Figure 2:**
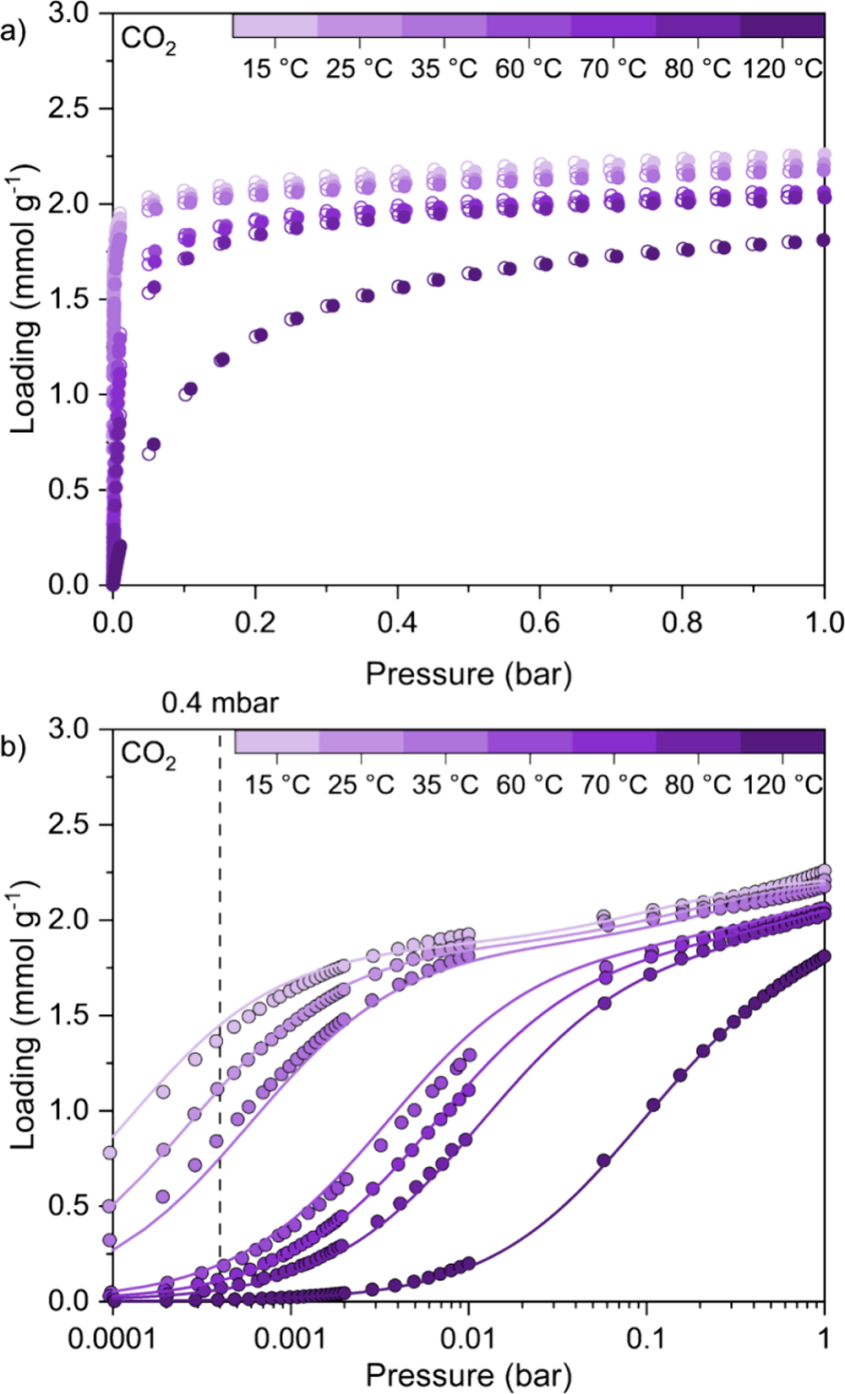
(a) CO_2_ equilibrium adsorption (filled symbols) and desorption (open symbols) isotherms measured at 15, 25, 35, 60, 70, 80, and 120 °C up to 1 bar for TIFSIX-3-Ni powder. (b) CO_2_ equilibrium adsorption isotherms shown with a log-scale of pressure measured at 15, 25, 35, 60, 70, 80, and 120 °C up to 1 bar for TIFSIX-3-Ni powder. Solid lines represent the fitting results from the DSL isotherm model, whose fitting parameters are found in Table 1.

We measured equilibrium CO_2_ adsorption and desorption isotherms up to 1 bar for TIFSIX-3-Ni powder at 15, 25, 35, 60, 70, 80, and 120 °C (Figure 2a). We confirmed equilibrium was reached for these analyses by measuring the isotherms at 35 °C using 60 and 90 s as the equilibrium interval and observing no change in the results (Figure S6).

According to Ullah et al.,^19^ the CO_2_ capture mechanism in TIFSIX-3-Ni is that one CO_2_ molecule fits within the cavity of a single unit cell cage, giving a theoretical maximum gravimetric CO_2_ loading for TIFSIX-3-Ni of 2.6 mmol g^–1^ (see SI for details). At 0.4 mbar, which is representative of the CO_2_ feed stream for a DAC process, and at an adsorption temperature of 25 °C, TIFSIX-3-Ni has a gravimetric CO_2_ adsorption capacity of 1.11 mmol g^–1^. This is very similar to the value reported by Kumar et al. (1.05 mmol g^–1^)^9^ and is higher than the gravimetric CO_2_ adsorption capacity measured at the same conditions for Lewatit VP OC 1065 (0.95 mmol g^–1^),^5^ a current benchmark adsorbent for DAC.

The isotherms of 15, 25, and 35 °C seem to converge at the same saturation capacity at 1 bar, and the same can be observed for the 60, 70, and 80 °C isotherms. This convergence of CO_2_ isotherms can also be observed in the isotherms reported by Kumar et al. in their Supporting Information.^9^ Interestingly, the CO_2_ loadings at 1 bar for the 15, 25, and 35 °C isotherms are all ∼2.2 mmol^–1^ g^–1^, and those of the 60, 70, and 80 °C isotherms are ∼2.0 mmol g^–1^. Both of these values are below the maximum theoretical loading of 2.6 mmol g^–1^. We hypothesized that this could be due to defects in the crystal structure and/or impurities in our sample which would decrease the number of adsorption sites for CO_2_. We performed XPS analysis on as-synthesized TIFSIX-3-Ni to investigate this and identified a non-negligible presence of oxygen in our sample (Table S2, approximately 5 at%). Deconvolution of the O 1*s*, N 1*s*, Ti 2*p*, and Ni 2*p* core levels (Figure S7) suggested the presence of NiO/Ni(OH)_2_ and TiO_2_/TiOF_2_ species and interaction of oxygen with the pyrazine linker,^48,49^ perhaps due to surface oxidation of the TIFSIX-3-Ni sample during synthesis, storage, and/or handling. Indeed, Barsoum et al. recently reported the presence of nickel oxide nanolayers on SIFSIX-3-Ni (the silicon analogue of TIFSIX-3-Ni) after exposure to humidity, though these were at different conditions (70 °C and 80% RH) than those that would be experienced by our sample during synthesis and/or storage.^50^ No anomalies were observed in the C 1*s* and F 1*s* spectra.

To enable the use of the adsorption data in process models, we fitted the experimental CO_2_ isotherms using isotherm model equations using the dual-site Langmuir (DSL) isotherm model shown below in eqs 7 to (9):
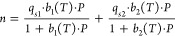
7
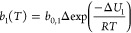
8
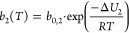
9where *T* is the temperature, *P* is the pressure, *q*_*s*1_ and *q*_*s*2_ are the maximum CO_2_ capacities, *b*_1_(*T*) and *b*_2_(*T*) are the CO_2_ adsorption coefficients defined by constant *b*_0,1_ and *b*_0,2_ and the internal energy change during adsorption Δ*U*_1_ and Δ*U*_2_, respectively, and *R* is the universal gas constant. The fitting results are shown in Figure 2b, with the fitted parameters and corresponding uncertainty bounds found in Table 1. The lower and upper bounds applied on each parameter during the fitting process are summarized in Table S3.

**Table 1:** Fitting Parameters with Uncertainty Bounds for a 95% Confidence Interval for the DSL, SSL, and UNIV6 Isotherm Models for CO_2_, N_2_, and H_2_O Adsorption on TIFSIX-3-Ni, Respectively

Adsorbate	Equation	Parameter	Unit	TIFSIX-3-Ni
CO_2_	DSL	*q*_*s*1_	mmol g^–1^	0.38737 ± 0.02037
		*b*_0,1_	(×10^–5^) bar^–1^	3.4932 ± 0.4670
		–Δ*U*_1_	kJ mol^–1^	29.256 ± 0.360
		*q*_*s*2_	mmol g^–1^	1.8730 ± 0.0085
		*b*_0,2_	(×10^–7^) bar^–1^	1.2200 ± 0.0355
		–Δ*U*_2_	kJ mol^–1^	59.798 ± 0.079
N_2_	SSL	*q*_*s*_	mmol g^–1^	1.7934 ± 0.0059
		*b*_0_	(×10^–5^) bar^–1^	1.9277 ± 0.0069
		–Δ*U*	kJ mol^–1^	21.175 ± 0.009
H_2_O	UNIV6	*q*_*s*_	mmol g^–1^	79.519 ± 1.780
		*α*_1_	–	0.069935 ± 0.003326
		*α*_2_	–	0.62604 ± 0.02311
		*α*_3_	–	0.022151 ± 0.004976
		*ε*_1_	kJ mol^–1^	45.444 ± 1.089
		*ε*_2_	kJ mol^–1^	33.169 ± 0.092
		*ε*_3_	kJ mol^–1^	37.485 ± 0.201
		*ε*_4_	kJ mol^–1^	31.471 ± 0.243
		*m*_1_	kJ mol^–1^	2.6902 ± 0.6773
		*m*_2_	kJ mol^–1^	0.59117 ± 0.04319
		*m*_3_	kJ mol^–1^	0.056502 ± 0.141182
		*m*_4_	kJ mol^–1^	2.8646 ± 0.1485
		*K*	(×10^–5^) bar^–1^	3.6905 ± 0.1113

Interestingly, the single-site Langmuir (SSL) isotherm model did not accurately represent our experimental data (Figure S8 and Table S4), particularly at the higher pressures and lower temperatures. This suggests additional binding sites for CO_2_ other than within the cavity of a unit cell cage as previously reported,^19^ which would support our previous hypotheses of the presence of defects, the presence of oxides of Ni and/or Ti, and/or the flexibility of the crystal structure.

#### 3.2.2 N_2_, Ar, and O_2_ Adsorption

**Figure 3:**
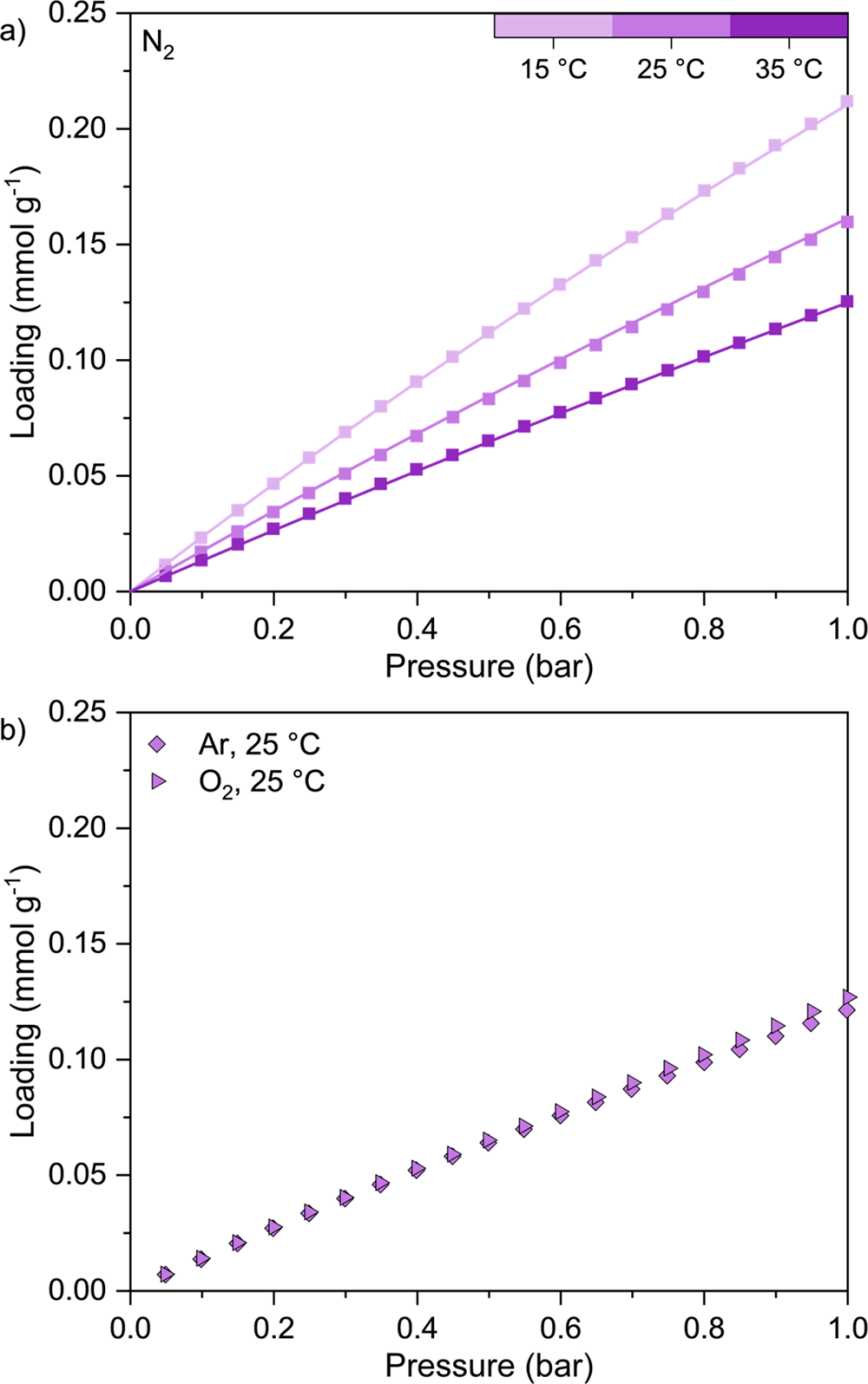
(a) N_2_ equilibrium adsorption isotherms for (squares) measured at 15, 25, and 35 °C. (b) Ar (diamonds) and O_2_ (triangles) equilibrium adsorption isotherms measured at 25 °C up to 1 bar for TIFSIX-3-Ni. Solid lines represent the fitting results from the SSL isotherm model, whose fitting parameters are found in Table 1.

We measured N_2_ adsorption and desorption isotherms up to 1 bar for TIFSIX-3-Ni powder at 15, 25, and 35 °C, as well as Ar and O_2_ adsorption and desorption isotherms up to 1 bar at 25 °C (Figure 3, desorption data in Figure S9).

N_2_, Ar, and O_2_ are more abundant than CO_2_ in air and could compete for CO_2_ adsorption sites. For TIFSIX-3-Ni, the adsorption levels of all three gases are very low compared to CO_2_, with N_2_ showing slightly higher adsorption compared to Ar and O_2_ at the same temperature. However, these adsorption levels are a magnitude higher than those observed for Lewatit VP OC 1065.^5^ As a result, the CO_2_ selectivity of TIFSIX-3-Ni is likely lower than that of the resin, which is not unexpected when comparing a physisorbent to a chemisorbent. We used the SSL isotherm model shown below in eq 10 to represent the measured N_2_ isotherm data:
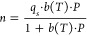
10where *n* is the loading, *P* is the pressure, *T* is the temperature, *q*_*s*_ is the maximum CO_2_ capacity, and *b*(*T*) is given by eq 8. The fitting parameters and corresponding uncertainty bounds are reported in Table 1, while the lower and upper bounds applied on each parameter during the fitting process are summarized in Table S3.

#### 3.2.3 H_2_O Adsorption

**Figure 4:**
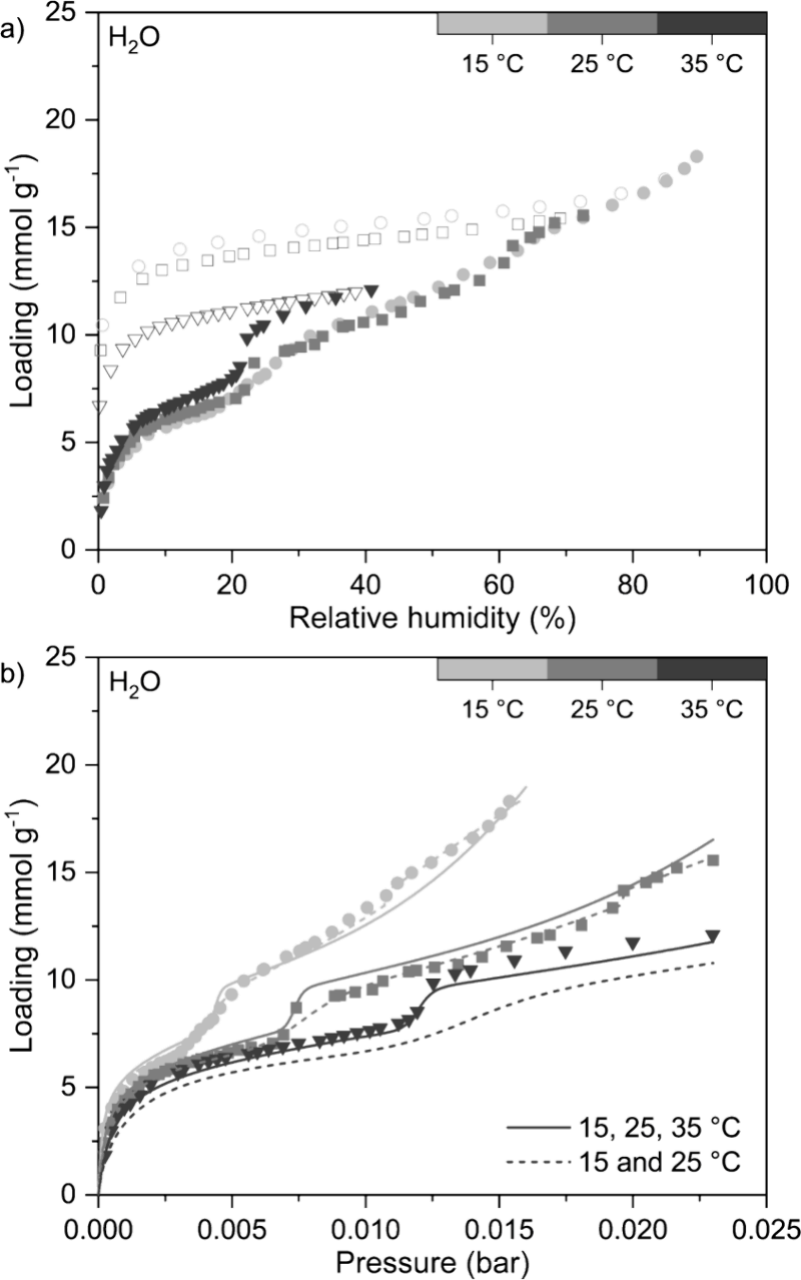
(a) H_2_O equilibrium adsorption (filled symbols) and desorption (open symbols) isotherms measured at 15 °C (circles), 25 °C (squares), and 35 °C (triangles) for TIFSIX-3-Ni as a function relative humidity. (b) H_2_O equilibrium adsorption isotherms measured at 15 °C (circles), 25 °C (squares), and 35 °C (triangles) as a function of absolute pressure in bar. Solid lines represent the UNIV6 isotherm model fitting results determined from using the 15, 25, and 35 °C experimental data in the fitting process, while dashed lines represent the fitting results using only the 15 and 25 °C experimental data. Fitting parameters are found in Table 1 and Table S6, respectively.

We measured H_2_O adsorption and desorption isotherms for TIFSIX-3-Ni powder at 15 °C up to 0.016 bar (94% RH), and at 25 and 35 °C up to 0.023 bar (73% RH and 41% RH, respectively) (Figure 4). We note that we measured these H_2_O isotherms on a different batch of TIFSIX-3-Ni, denoted by the gray color scheme instead of purple, as compared to all previous measurements up to this point. Differences in batches of TIFSIX-3-Ni are discussed later in Section 3.3.1.

TIFSIX-3-Ni has almost three times higher H_2_O loading compared to Lewatit VP OC 1065 at 25 °C and 70% RH,^5^ and displays an unusual stepwise adsorption behavior at all temperatures along with severe hysteresis demonstrating irreversible adsorption. This stepwise adsorption was observed previously by Mukherjee et al.,^12^ but desorption data was not measured. They reported slightly lower loading at 25 °C (∼13 mmol g^–1^ at 0.025 bar, *i.e.*, 79% RH) and only observed a single step at ∼0.008 bar (*i.e.*, 25% RH). They also reported a step decrease in the H_2_O loading after 0.025 bar, a humidity level we were not able to measure up to with our apparatus.

According to Ullah et al.,^19^ four H_2_O molecules can fit within the cavity of a single TIFSIX-3-Ni unit cell cage, leading to a theoretical maximum H_2_O loading of 10.5 mmol g^–1^. However, from Figure 4, the adsorption isotherms measured at all temperatures exceed this value. Furthermore, the data measured at 35 °C does not overlay with those measured at 15 and 25 °C when the loading data is plotted as a function of the relative humidity (Figure 4a), as well as the adsorption potential ϵ^51^ (Figure S10), given by eq 11:
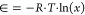
11where *R* is the universal gas constant, *T* is the temperature, and *x* is the relative pressure. These observations (*i.e.*, unusual stepwise adsorption, hysteresis, exceeding the theoretical H_2_O loading, and apparent disagreement of the 35 °C isotherm) again suggest that the TIFSIX-3-Ni sample may have flexibility/defects in its crystal structure, and/or be degrading upon exposure to H_2_O, both of which have recently been observed for SIFSIX-3-Ni.^50^ A more thorough investigation of the H_2_O interaction with, and H_2_O stability of, TIFSIX-3-Ni is discussed in Section 3.4.1.

According to the IUPAC classification,^29^ the stepwise H_2_O adsorption behavior follows that of a Type VI isotherm. This can be modeled using the universal adsorption isotherm model for a Type VI isotherm reported by Ng et al.:^38^
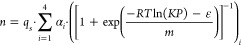
12
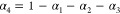
13where *n* is the loading, *T* is the temperature, *P* is the pressure, *q*_*s*_ is the saturation capacity, *K* is the adsorption equilibrium constant, *α*_*i*_ are probability factors, *ε*_*i*_ is the adsorption energy site with maximum frequency, *m*_*i*_ is the surface heterogeneity, and *R* is the universal gas constant. From here on, we refer to this model as the UNIV6 isotherm model.

Figure 4b shows the fitted results from using the isotherm data measured at all three temperatures for the fitting process, as well as using only the 15 and 25 °C isotherm data to obtain the fitting parameters. The first method provides a relatively good fit for the experimental data at all three temperatures, although the presence of the third adsorption step for 15 and 25 °C is not captured. The latter procedure provides an excellent fit of the experimental data at 15 and 25 °C, but underestimates the loading at 35 °C. This is not unexpected given that the 35 °C loading data did not overlap with the 15 and 25 °C data when plotted against the relative humidity and adsorption potential as discussed previously. Overall, the fitting parameters obtained using all three temperatures would be more representative of the experimental data for process modeling, and these are reported in Table 1 along with their corresponding uncertainty bounds. The lower and upper bounds applied on each parameter during the fitting process are summarized in Table S5. The fitting parameters and corresponding uncertainty bounds obtained using only the 15 and 25 °C isotherm data are presented in Table S6, along with the lower and upper bounds applied on each parameter during the fitting process.

#### 3.2.4 Heats of Adsorption

**Figure 5:**
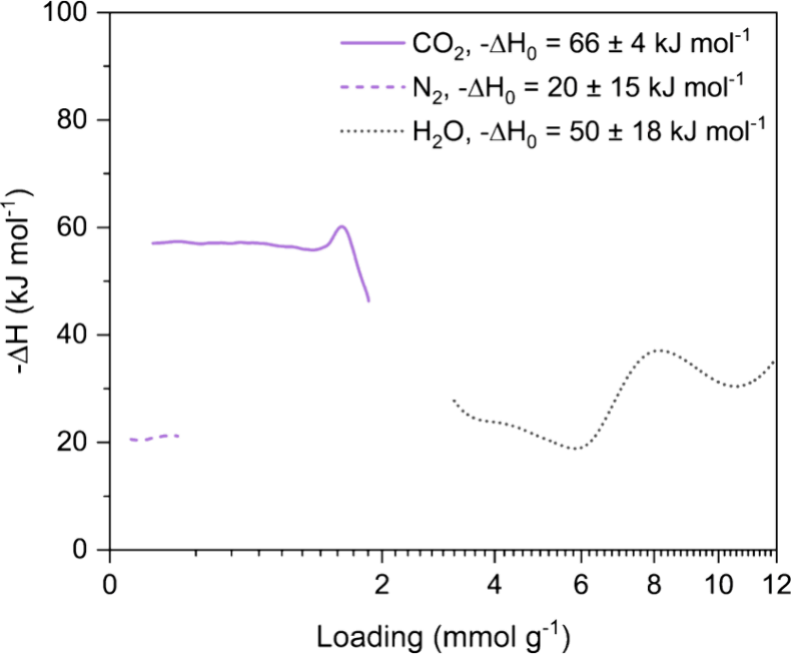
CO_2_, N_2_, and H_2_O isosteric heats of adsorption for TIFSIX-3-Ni with the loading plotted on square root scale for clarity. The Δ*H*_0_ values correspond to the limiting heats of adsorption.

We calculated the CO_2_, N_2_, and H_2_O heats of adsorption as a function of loading (*i.e.*, isosteric heat of adsorption), as well as the limiting heats of adsorption (Figure 5), for TIFSIX-3-Ni. These parameters are necessary inputs in process models and relate to the energy required for adsorbate desorption in a DAC process, where a higher heat of adsorption means higher desorption energy needed. Confidence intervals linked to the isosteric heats of adsorption calculations are presented in Figure S11, along with results determined using an alternative set of isotherm temperatures for CO_2_. The smoothing parameter values used for each gas as described in Section 2.6 are presented in Table S7. The intermediate calculation steps for the limiting heats of adsorption as described in Section 2.6 are presented in Figures S12 to S14.

The CO_2_ isosteric heat of adsorption presented in Figure 5 is based on the 60, 70, 80, and 120 °C CO_2_ isotherm data, as calculations done with the 15, 25, and 35 °C isotherms resulted in large uncertainty bounds at loading levels above ∼1.7 mmol g^–1^, corresponding with the convergence of the isotherms. At the loading level corresponding to 0.4 mbar CO_2_, TIFISX-3-Ni has a heat of adsorption of ∼56 kJ mol^–1^, which agrees well with the values previously reported in the literature.^9,12^ This is also significantly lower than the CO_2_ heat of adsorption at the same loading of Lewatit VP OC 1065 (81 kJ mol^–1^).^5^ The heat of adsorption stays relatively constant up to a loading of approximately 1.5 mmol g^–1^, indicating the presence of homogeneous CO_2_ binding sites, *i.e.*, the cavity of each cage. Beyond this loading, the heat of adsorption begins to decrease, which corresponds with the convergence of the isotherms at higher loadings we observed in Figure 2. This suggests the presence of additional CO_2_ binding sites of lower energy and correlates with the need to use the DSL model to fit the CO_2_ experimental isotherm data instead of the SSL model. As discussed previously, these additional binding sites could be due to defects, the presence of oxides of Ni and/or Ti, and/or flexibility of the crystal structure. For N_2_, TIFSIX-3-Ni exhibits a low and constant heat of adsorption of ∼20 kJ mol^–1^, where the latter indicates homogeneous interaction with the adsorbent. This was also observed for Lewatit VP OC 1065.^5^ For H_2_O, we determined the limiting heat of adsorption to be 50 kJ mol^–1^, but were unable to calculate the subsequent isosteric heat of adsorption between 0 and 3 mmol g^–1^ due to the lack of experimental data in this range. Between 3 to 6 mmol g^–1^ the heat of adsorption averages between 20 to 25 kJ mol^–1^, after which it increases to between 30 to 40 kJ mol^–1^. This increase in heat of adsorption corresponds with the loading level at which we observe the first step in the experimental H_2_O isotherm data at all temperatures. We note however that there is a large uncertainty range in the calculated isosteric heat of adsorption for H_2_O (Figure S11), likely associated with the previously discussed discrepancy between the 35 °C isotherm and the 15 and 25 °C isotherms.

To put the adsorption performance of TIFSIX-3-Ni into context, we provide in Table 2 a comparison of the CO_2_ isosteric heats of adsorption (corresponding to the loadings at ∼0.4 mbar CO_2_) and CO_2_, N_2_, and H_2_O uptakes between TIFSIX-3-Ni and other physical adsorbents previously studied for DAC. Among these adsorbents, TIFSIX-3-Ni has one of the highest CO_2_ adsorption capacities at the low pressures relevant for DAC, and comparable CO_2_ heat of adsorption and N_2_ and H_2_O uptakes. The closest material to TIFSIX-3-NI with respect to these features is NbOFFIVE-1-Ni.

**Table 2:** Summary of the CO_2_ Isosteric Heats of Adsorption and CO_2_, N_2_, and H_2_O Uptakes of TIFSIX-3-Ni and Other Physical Adsorbents Investigated for DAC

Adsorbent	–Δ*H* (kJ mol^–1^)	CO_2_ uptake @ 0.5 mbar, 25 °C (mmol g^–1^)	N_2_ uptake @ 1 bar, 25 °C (mmol g^–1^)	H_2_O uptake @ 95% RH, 25 °C (mmol g^–1^)	Reference
TIFSIX-3-Ni	56	1.2	0.16	15.6* (*73% RH, 25 °C)	This work
Zeolite 13X	39	0.4	0.42	18.8	(12)
Mg-MOF-74	42	0.05	0.85	33.3	(12)
SIFSIX-3-Ni	45	0.4	0.16	8.8	(12)
SIFSIX-18-Ni-β	52	0.4	0.04	1.6	(12)
NbOFFIVE-1-Ni	54	1.3	0.15	10.1	(12)

### 3.3 Manufacturing Considerations

The ability to manufacture consistent batches of TIFSIX-3-Ni, both as powder and in shaped form should be investigated to understand the deployment potential of this MOF.

#### 3.3.1 Batch Reproducibility

Lack of batch-to-batch conformity in TIFSIX-3-Ni will impact its materials and adsorption properties and thereby process performance metrics. To investigate this, we synthesized various batches of NiTiF_6_, TIFSIX-3-Ni precursor, and final TIFSIX-3-Ni powder at various times, scales, and by separate researchers. These details are summarized below in Table 3.

**Table 3:** Summary of the Synthesis Procedure for Different Batches of TIFSIX-3-Ni

NiTiF_6_ batch	TIFSIX-3-Ni precursor batch	TIFSIX-3-Ni batch	TIFISX-3-Ni synthesis scale	Synthesizing researcher (all)	Synthesis date (NiTiF_6_/TIFSIX-3-Ni)
A	1	1	1×	MYL	09/01/21–09/13/21
B	2	2	1×	DD	12/02/21–12/10/21
C	3	3	1×	MYL	12/02/21–12/10/21
A	4	4	4×	MYL	09/01/21–12/10/21
C	5	5	4×	MYL	12/02/21–05/04/22

**Figure 6:**
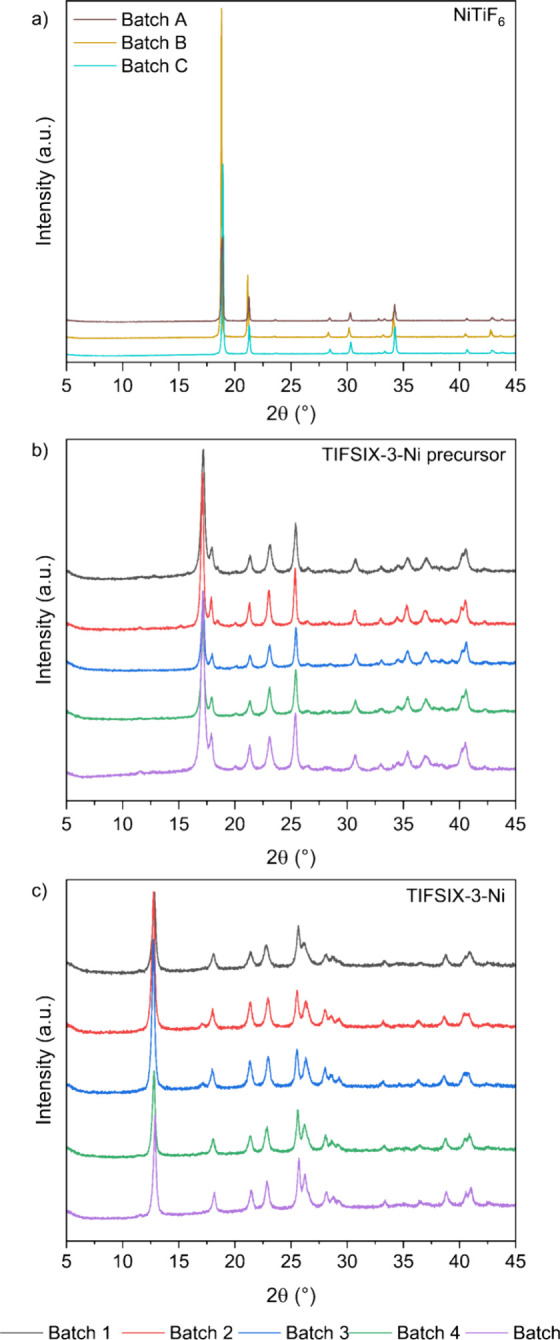
XRD patterns of synthesized batches of (a) NiTiF_6_, (b) TIFSIX-3-Ni precursor, and (c) final TIFSIX-3-Ni.

The XRD patterns of NiTiF_6_, TIFSIX-3-Ni precursor, and final TIFSIX-3-Ni samples are shown in Figure 6. The same peaks are present and are not shifted between the different batches of each material, indicating good reproducibility of the crystal structure. We do note however the presence of an extra peak at 17° in batches 2 and 3 of TIFSIX-3-Ni, which might be associated with incomplete conversion of the precursor to the final TIFSIX-3-Ni product.

**Figure 7:**
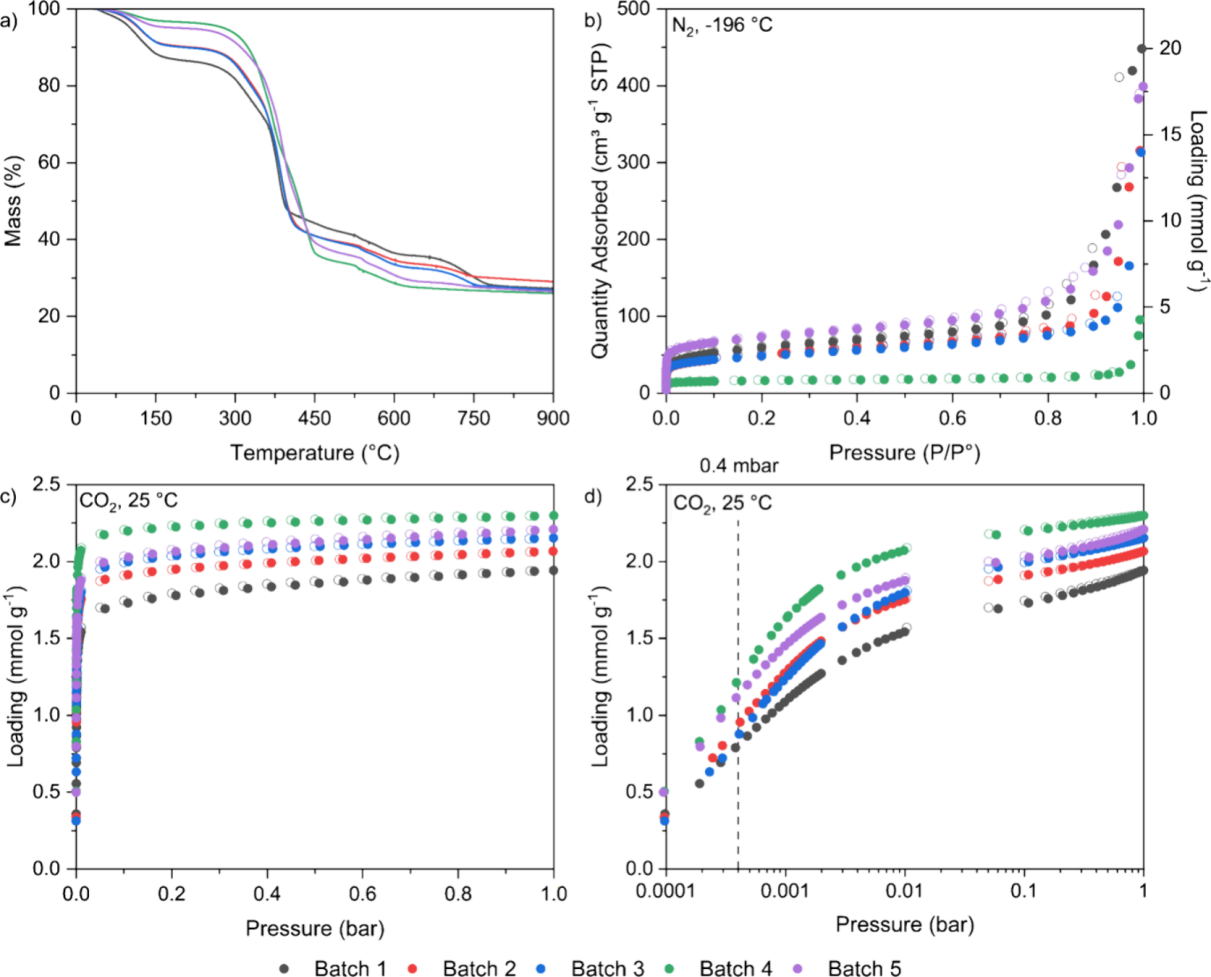
(a) TG curves under N_2_ air atmosphere up to 900 °C, (b) N_2_ adsorption isotherms at −196 °C, and CO_2_ adsorption isotherms at 25 °C with (c) linear and (d) log-scale of pressure (filled symbols are adsorption; open symbols are desorption) for different batches of TIFSIX-3-Ni.

The TG curves of the different batches of TIFSIX-3-Ni are shown in Figure 7a. All batches appear to lose water content up to 150 °C, with degradation of the main crystal structure starting at 230 °C. There are slight variations in the mass loss between the different batches, but the overall shape of the TG curve is similar between all samples.

The N_2_ adsorption data at −196 °C are shown in Figure 7b, with log–log scales of pressure and loading shown in Figure S15a, from which we determined the BET areas, micropore volumes, and total pore volumes as described in Section 2.4. We observed significant variation in these parameters (>70%) as shown below in Table 4. The CO_2_ adsorption at 25 °C results are shown in Figure 7c and 7d and summarized in Table 4. Here, there is a 35% variation between the highest and lowest values of CO_2_ adsorption at 0.4 mbar. There does not seem to be a correlation between the textural properties of the samples and the CO_2_ adsorption, as the batch with the lowest BET area, micropore volume, and total pore volume has the highest CO_2_ adsorption (batch 4), and the batch with the highest BET area, micropore volume, and total pore volume has the second highest CO_2_ adsorption (batch 5). This suggests that N_2_ and CO_2_ have different mechanisms to access the pore structure.

**Table 4:** Summary of the Textural Properties Derived from N_2_ Adsorption at −196 °C, and CO_2_ Adsorption at 0.4 mbar and 25 °C for Different Batches of TIFSIX-3-Ni

TIFSIX-3-Ni batch	*S*_BET_ (m^2^ g^–1^)	*V*_micropores_ (cm^3^ g^–1^)	*V*_total pores_ (cm^3^ g^–1^)	CO_2_ uptake @ 0.4 mbar, 25 °C (mmol g^–1^)
1	213	0.11	0.65	0.79
2	175	0.08	0.42	0.96
3	175	0.08	0.26	0.88
4	62	0.03	0.06	1.21
5	264	0.11	0.50	1.11

Overall, it appears that the crystal structure and thermal stability are reproducible between different batches of TIFSIX-3-Ni despite different researchers, times of synthesis, and scale, however there is wide variation in the textural properties and CO_2_ adsorption, which may be linked to the presence of impurities/defects as highlighted earlier. All the material and adsorption properties discussed in previous sections were measured using batch 5 TIFSIX-3-Ni with the results shown in purple, except for the H_2_O adsorption isotherms which were measured with batch 1 TIFSIX-3-Ni and these results are shown in gray.

#### 3.3.2 Pelletization

TIFSIX-3-Ni is synthesized as a powder, however, adsorbents in this form would not be practical in an actual process as they would be difficult to handle, and their dense packing would result in high pressure drop and poor heat and mass transfer. The ability to shape TIFSIX-3-Ni powder into a structured form such as pellets without significantly affecting its performance for DAC is therefore another important factor to investigate.

We pelletized TIFSIX-3-Ni powder (batch 5) without any binders by applying a pressure equivalent to ∼37 MPa. This pressure is well below the compression pressure of 340 MPa previously reported to induce irreversible amorphization on ZIF-8.^52^ A study by Ribeiro et al. also demonstrated that compression pressures of 62 and 125 MPa still allowed the crystallinity of ZIF-8 and MIL-53(Al) to be maintained, though reductions in the textural properties of ∼10% and ∼30 to 40%, respectively, were observed.^53^

**Figure 8:**
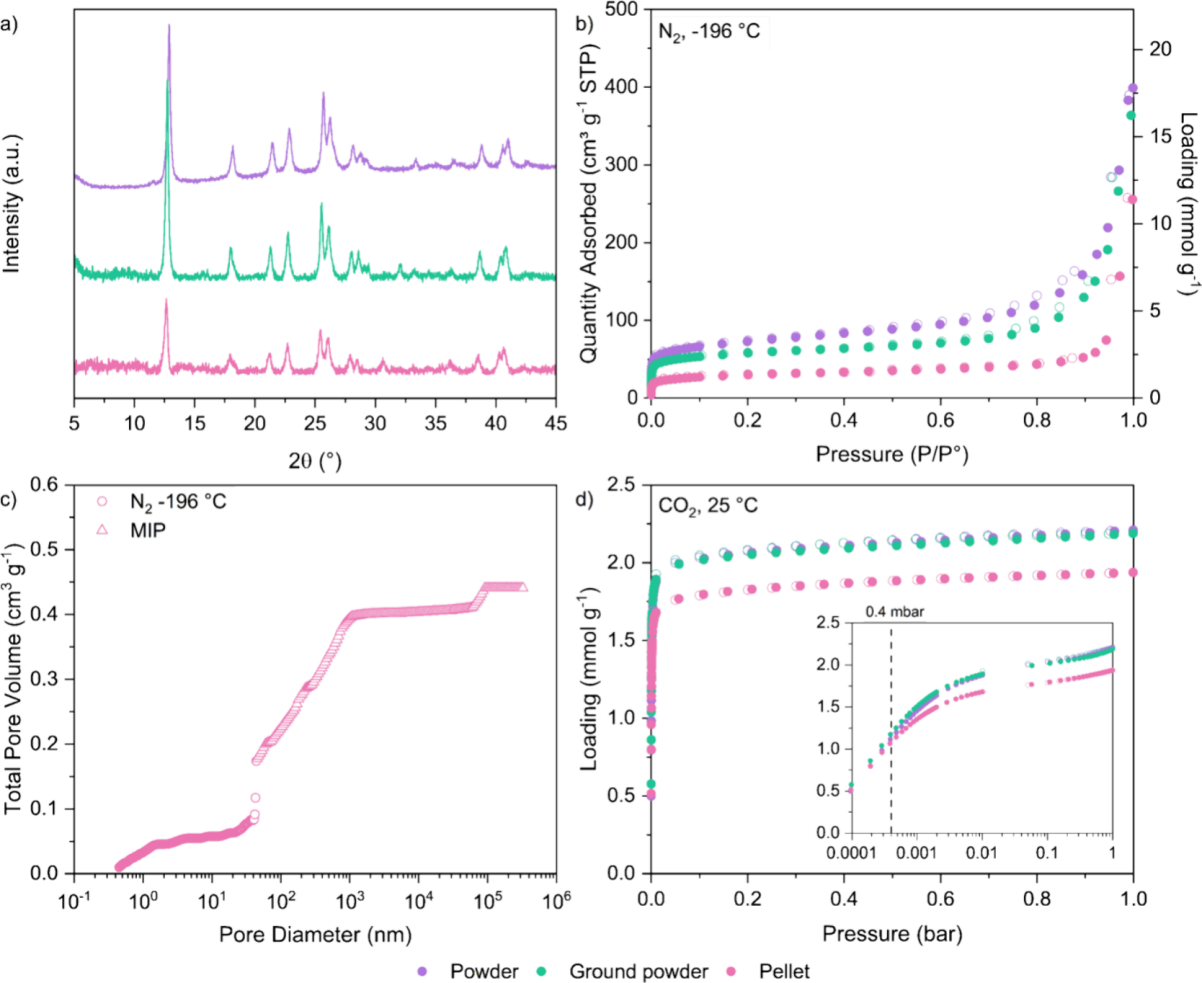
(a) XRD patterns and (b) N_2_ adsorption (filled symbols) and desorption (open symbols) isotherms at −196 °C of batch 5 TIFSIX-3-Ni powder, ground powder, and pellet. (c) Cumulative pore volume as a function of pore diameter of TIFSIX-3-Ni pellet, obtained from N_2_ adsorption at −196 °C (circles) and mercury intrusion porosimetry (triangles). (d) CO_2_ adsorption (filled symbols) and desorption (open symbols) isotherms at 25 °C for batch 5 TIFSIX-3-Ni powder, ground powder, and pellet, with log-scale of pressure shown in the inset.

The XRD patterns of the as-synthesized TIFSIX-3-Ni powder, intermediate ground powder, and final pellet are shown in Figure 8a. Overall, the crystallinity of TIFSIX-3-Ni appears to be maintained in pellet form. Yet, we note that most peaks are very slightly left-shifted in the ground powder and pellet sample compared to the original powder. There is also an additional peak that appears in the ground powder sample at 32°, which is then left-shifted in the pellet sample. Based on the experiments conducted, we are uncertain of the cause of this observation.

The N_2_ adsorption data at −196 °C are reported in Figure 8b, with log–log scales of pressure and loading shown in Figure S15b, from which we determined the textural parameters. Grinding of TIFSIX-3-Ni powder resulted in a 20% decrease in the BET area, micropore volume, and total pore volume of the sample, while pelletization resulted in a further 30% to 40% decrease for all three parameters, with final values of 106 m^2^ g^–1^, 0.06 cm^3^ g^–1^, and 0.24 cm^3^ g^–1^, respectively. For pelletized samples, macropores contribute significantly to the total pore volume. As in our previous work,^33^ we estimated the total pore volume of the pelletized sample through a combination of N_2_ adsorption and MIP measurements (Figure 8c), yielding a total pore volume of 0.44 cm^3^ g^–1^. The incremental pore size distribution is shown in Figure S16. The MIP measurement also provided us with the pellet density, determined to be 0.81 g cm^–3^. This value is similar to the pellet density of 0.85 g cm^–3^ reported for a binder-free zeolite 13X pellet.^33^ Using these values, we then calculated the bed density, skeletal density, and porosity of the pellet sample, all necessary inputs in process models. These values, along with the textural properties of the as-synthesized powder and ground powder samples, are summarized below in Table 5.

**Table 5:** Summary of Textural Properties and Densities of TIFSIX-3-Ni Powder, Ground Powder, and Pellet (Batch 5), as Derived from N_2_ Adsorption at −196 °C and MIP Measurements

TIFSIX-3-Ni (batch 5)	*S*_BET_ (m^2^ g^–1^)	*V*_micropores_ (cm^3^ g^–1^)	*V*_total pores_ (cm^3^ g^–1^)	ρ_skeletal_ (g cm^–3^)	ρ_pellet_ (g cm^–3^)	ρ_bed_ (g cm^–3^)	ε_pellet_
Powder	264	0.11	0.50	–	–	–	–
Ground powder	213	0.09	0.41	–	–	–	–
Pellet	106	0.06	0.44	1.26	0.81	0.58	0.36

For further analysis, we measured CO_2_ adsorption at 0 °C for all three samples to see if this could better access the ultramicropores of TIFSIX-3-Ni, as N_2_ at −196 °C may be kinetically restricted when trying to enter these pores.^54^ The measured isotherms are shown in Figure S17, and the calculated textural properties are summarized and compared with that of N_2_ adsorption at −196 °C in Table S8. From the results, CO_2_ can access the same amount of micropores in all three samples at 0 °C, while N_2_ at −196 °C appears to be more restricted in accessing some of the pores in the pellet sample. Pelletization may have compressed the sample’s crystal structure and reduced the pore size to an extent which further hindered N_2_ access, but left CO_2_ unaffected. However, due to the inability of CO_2_ at 0 °C to probe the larger pores, and the lack of suitable DFT models to calculate the pore size distribution and thus total pore volume, we continue to use N_2_ adsorption at −196 °C to assess the textural properties of TIFSIX-3-Ni throughout this study. We observed an unusual hysteresis (as well as the beginning of an inflection point) in the CO_2_ 0 °C isotherm in Figure S17 for the pellet sample, which might indicate flexibility in the crystal structure. Other observations discussed previously such as the unusual trend of the specific heat capacity and the behaviors of the CO_2_ and H_2_O isotherms also suggested possible flexibility in the crystal structure. The pyrazine linkers in TIFSIX-3-Ni are only coordinated at one point on each pole, which would allow a rotational degree of freedom. This was indeed recently reported for SIFSIX-3-Ni,^50^ and also previously observed for ZIFs-7, −8, −11, −65, −71, and −90.^47,55−58^ We tested this hypothesis by performing *in situ* XRD measurements of both powder and pellet samples of TIFSIX-3-Ni under ambient air, then under a flow of N_2_ at room temperature and 160 °C, and then under a flow of CO_2_ at room temperature to observe how the samples might change at different stages of a DAC process. Figure S18a and S18b reveal peak shifts and new peaks under the different conditions, as well as differences between the powder and pellet sample that were not present in Figure 8a, that could further suggest flexibility or change in the TIFSIX-3-Ni crystal structure. However, a dedicated investigation involving further measurements at higher pressures or lower temperatures, as well as structure refinement is needed to draw any quantitative conclusions.

Finally, to assess the impact of pelletization on the CO_2_ adsorption performance of TIFSIX-3-Ni, we measured CO_2_ adsorption isotherms at 25 °C up to 1 bar, shown in Figure 8d. The results show that grinding has minimal impact on the CO_2_ adsorption of the sample across the whole pressure range. Pelletization causes a ∼ 10% decrease in the CO_2_ adsorption capacity at 1 bar compared to the powder, but there is minimal difference at 0.4 mbar (Figure 8d inset). Therefore, while pelletization seems to have restricted N_2_ access (at least at −196 °C) within the crystal structure, it has not negatively impacted CO_2_ access at the relevant DAC conditions. Pelletized TIFSIX-3-Ni may therefore have higher CO_2_/N_2_ selectivity when compared to its powder form.

With a bed density of 0.58 g cm^–3^ and gravimetric CO_2_ adsorption capacity of 1.07 mmol g^–1^ at 0.4 mbar and 25 °C, the volumetric CO_2_ adsorption capacity of pelletized TIFSIX-3-Ni is 0.62 mmol cm^–3^_bed_ at these conditions. This is higher than the volumetric CO_2_ adsorption capacity of 0.45 mmol cm^–3^_bed_ previously reported for Lewatit VP OC 1065.^5^

We note that these results, along with the crystallinity and textural properties of TIFSIX-3-Ni, are dependent on the pelletization pressure used. For example, higher pressures could result in higher pellet and bed density but may potentially damage the crystal structure of TIFSIX-3-Ni and lower its gravimetric CO_2_ adsorption capacity. On the other hand, lower pressures may better preserve the crystal structure of TIFSIX-3-Ni but result in lower volumetric CO_2_ adsorption capacity. Studies could therefore be conducted in the future to determine an optimal pelletization pressure for TIFSIX-3-Ni.

### 3.4 Adsorbent Stability under Process Conditions

Although not a direct input for process modeling, the lifetime of an adsorbent based on its rate of degradation when subjected to various process conditions, will greatly influence the cost and carbon footprint associated with adsorbent production and disposal.^59^ Factors that could potentially impact the adsorbent include exposure to humidity, at both ambient and elevated temperatures, and exposure to multiple adsorption–desorption cycles.

#### 3.4.1 Humidity Studies

##### 3.4.1.1 Exposure to H_2_O Only

**Figure 9:**
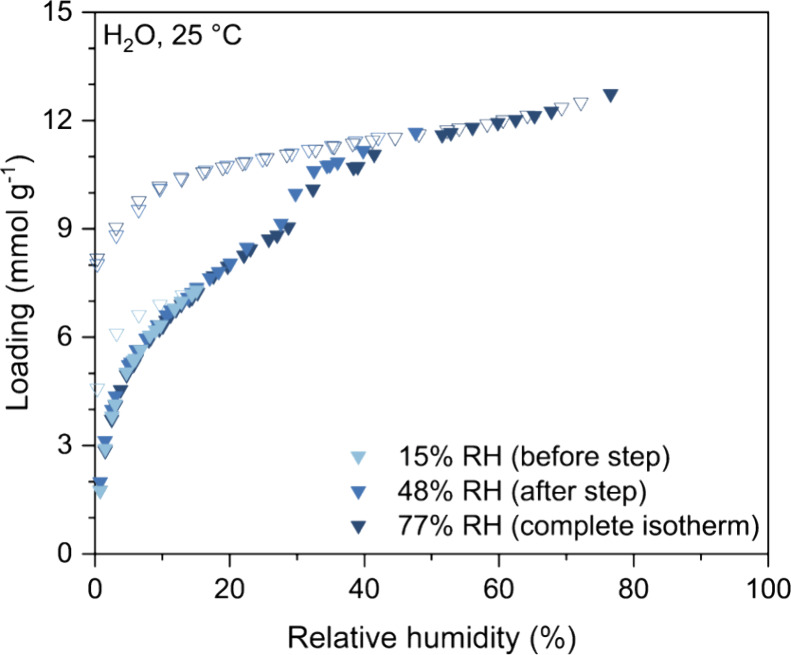
Equilibrium adsorption (filled symbols) and desorption (open symbols) isotherms for H_2_O measured at 25 °C for batch 5 TIFSIX-3-Ni powder up to 15, 48, and 77% RH.

To understand the stability of TIFSIX-3-Ni to humidity alone without the presence of oxygen, samples of batch 5 TIFSIX-3-Ni powder were used to measure H_2_O isotherms at 25 °C, up to 15% RH, 48% RH, and 77% RH, where the latter is the highest relative humidity our instrument can achieve at this temperature (Figure 9). These humidity levels correspond to the different steps observed in the H_2_O isotherms from Figure 4 (obtained with batch 1 TIFSIX-3-Ni), where 15% RH would be before the first isotherm step, 48% RH would be between the first and second isotherm step, and 77% RH would be the complete isotherm (after the second step). However, when we measured the H_2_O isotherms described above with batch 5 TIFSIX-3-Ni, we only observed one distinct step in the isotherm and achieved slightly lower loading compared to batch 1, both similar to that reported by Mukherjee et al.^12^ This observation indicates that the H_2_O adsorption can vary between different synthesized batches of TIFSIX-3-Ni, as we also observed with the N_2_ and CO_2_ adsorption in Section 3.3.1. We note again the presence of an irreversible hysteresis in these isotherms. The hysteresis is much more severe when the RH level is beyond that where we observe the first isotherm step, suggesting that above a certain RH level, the MOF may irreversibly react with water.

**Figure 10:**
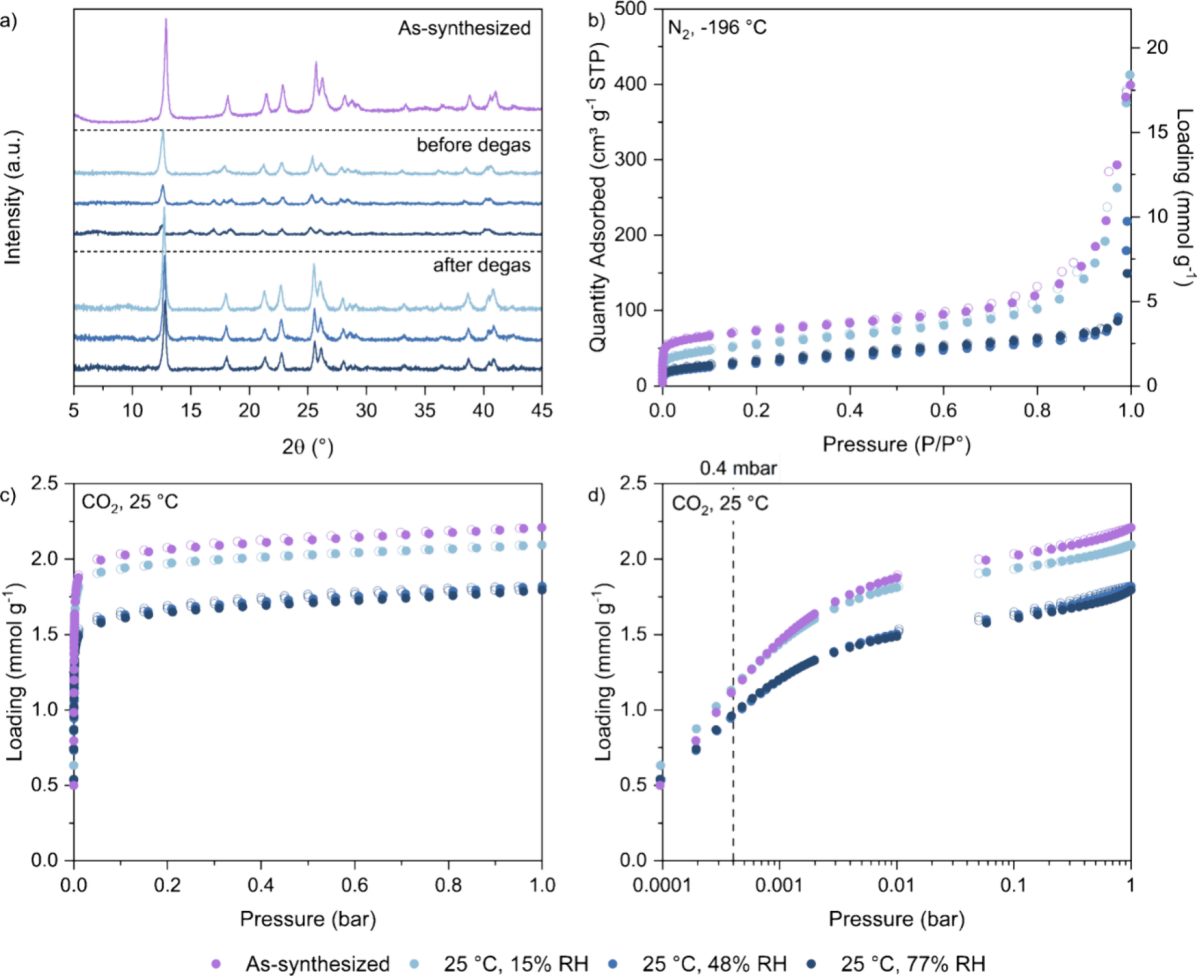
(a) XRD patterns, (b) N_2_ adsorption isotherms at −196 °C, and CO_2_ adsorption isotherms at 25 °C with (c) linear and (d) log-scale of pressure (filled symbols are adsorption; open symbols are desorption) for batch 5 TIFSIX-3-Ni powder before (purple) and after exposure to 15% RH (light navy), 48% RH (medium navy), and 77% RH of H_2_O (dark navy).

We then characterized the samples’ crystallinity, textural properties, and CO_2_ adsorption and compared them to that of the as-synthesized sample, as shown in Figure 10. We measured XRD patterns of the samples immediately after the H_2_O isotherm measurements, *i.e.*, when there should still be adsorbed water in the sample, and after the same samples were degassed. We then measured N_2_ adsorption isotherms at −196 °C and CO_2_ adsorption isotherms at 25 °C on these degassed samples. Figure 10a reports the changes in the XRD pattern of all three samples before degassing compared to the as-synthesized sample. These include a left shift in most peaks such as those originally at 12° and 26°, the presence of additional peaks in the 15 to 20° range, and the disappearance of peaks in the 37 to 40° range. These changes are more prominent in the 48% RH and 77% RH samples, *i.e.*, after the step in the H_2_O isotherm has occurred. After degassing of the samples, their XRD patterns are very similar to that of the as-synthesized sample, with all the original peaks still present, though some are now slightly left-shifted. We also observed a slight color change in the 48% RH and 77% RH samples as shown in Table S9, while the color of the 15% RH sample is very similar to that of the as-synthesized sample. Overall, there appears to be minimal change in the crystal structure of TIFSIX-3-Ni after exposure to H_2_O under these conditions and subsequent degassing.

We also performed XPS analysis on the degassed 77% RH sample. Compared to the as-synthesized sample, there appears to be a slight increase in the oxygen content in the sample (Table S10), and deconvolution of the O 1*s* and Ti 2*p* core level suggests an increased presence of oxides of Ti (*e.g.*, TiO_2_/TiOF_2_)^49^ (Figure S19). No distinct changes were observed for the C 1*s*, N 1*s*, F 1*s*, and Ni 2*p* spectra.

The N_2_ adsorption data at −196 °C are shown in Figure 10b and Figure S20a, from which we determined the textural parameters summarized in Table 6. After exposure to 15% RH, *i.e.*, before the first step in the H_2_O isotherm, TIFSIX-3-Ni experiences a 20% to 30% reduction in its textural properties. After exposure to 48% RH and 77% RH, *i.e.*, after the step in the H_2_O isotherm, there is a more significant reduction in the BET area and micropore volume of the samples of ∼60%, and a 75% loss in total pore volume. Hence, despite the XRD patterns suggesting a “recovery” of the initial crystallinity, it seems that while part of the crystalline structure is maintained/recovered after exposure to water beyond the H_2_O isotherm step, another part is lost and leads to a reduced porosity.

**Table 6:** Summary of the Textural Properties Derived from N_2_ Adsorption at −196 °C and CO_2_ Adsorption at 0.4 mbar and 25 °C for TIFSIX-3-Ni Samples Exposed to Various Stability Studies

Study	Sample	Condition	*S*_BET_ (m^2^ g^–1^)	*V*_micropores_ (cm^3^ g^–1^)	*V*_total pores_ (cm^3^ g^–1^)	CO_2_ uptake @ 0.4 mbar, 25 °C (mmol g^–1^)
H_2_O exposure	TIFSIX-3-Ni batch 5, powder	As-synthesized	264	0.11	0.50	1.11
		15% RH	196	0.08	0.41	1.13
		48% RH	109	0.05	0.14	0.94
		77% RH	120	0.05	0.13	0.96
H_2_O and O_2_ exposure	TIFSIX-3-Ni batch 3, powder	As-synthesized	175	0.08	0.26	0.88
		40 °C, 75% RH air, 14 days	36	0.02	0.05	0.51
		40 °C, 90% RH air, 14 days	49	0.02	0.05	0.47
		Ambient air, 6 months	40	0.02	0.05	0.39
Adsorption cycling	TIFSIX-3-Ni batch 5, pellet	As-synthesized	147	0.07	0.34	0.94
		Postcyclic	82	0.04	0.11	0.44

Lastly, from the CO_2_ isotherms shown in Figures 10c and 10d, we observe reductions in the CO_2_ adsorption capacity at 1 bar for all three samples which follows the reduction in porosity observed above. After exposure to 15% RH, the CO_2_ adsorption capacity decreased by ∼5% at 1 bar. After exposure to 48% RH and 77% RH, the CO_2_ adsorption capacity decreased by ∼20% at 1 bar. The CO_2_ loading at 0.4 mbar is summarized below in Table 6, where, despite the changes in the textural properties, we observe no significant decrease in the CO_2_ adsorption performance after exposure to 15% RH, and a 15% decrease after exposure to 48% RH and 77% RH. Mukherjee et al. reported a much more severe reduction in CO_2_ saturation capacity of ∼60% from their dynamic column breakthrough tests at 25 °C under a flow of 3000 ppm of CO_2_ and 74% RH.^12^ Ullah et al. reported a similar decrease in CO_2_ adsorption of ∼70% after temperature-programmed desorption (TPD) experiments conducted at 25 °C with pure CO_2_ and 75% RH.^19^ For both these cases, the comparison was between the CO_2_ uptake when coadsorbed with H_2_O, vs the CO_2_ uptake under a dry stream. In our experiments, we compared the single-component CO_2_ adsorption of a sample before and after exposure to pure H_2_O. Therefore, while our experiments suggest that the CO_2_ adsorption performance of TIFSIX-3-Ni is recoverable after exposure to humidity at ambient temperatures, results in the literature suggest that the CO_2_ adsorption will be more severely limited when coadsorbed with H_2_O at similar conditions. In addition, since there is already a ∼ 15% decrease in CO_2_ adsorption capacity at 0.4 mbar for the samples exposed to 48% RH and 77% RH at 25 °C, it is unlikely that TIFSIX-3-Ni would be able to withstand a steam desorption process.

##### 3.4.1.2 Exposure to H_2_O and O_2_

**Figure 11:**
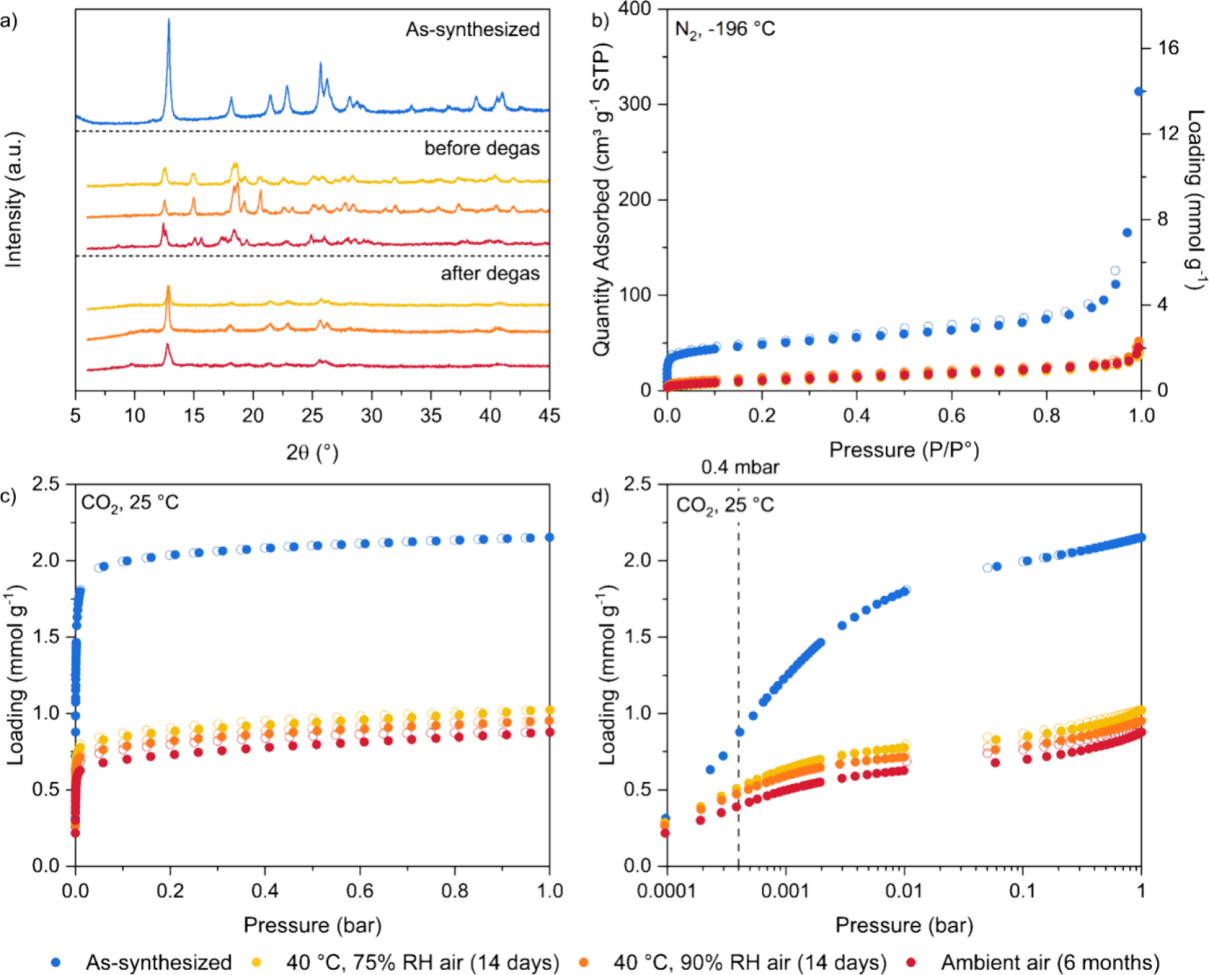
(a) XRD patterns, (b) N_2_ adsorption isotherms at −196 °C, and CO_2_ adsorption isotherms at 25 °C with (c) linear and (d) log-scale of pressure (filled symbols are adsorption; open symbols are desorption) for batch 3 TIFSIX-3-Ni powder before (blue) and after exposure to 40 °C and 75% RH air for 14 days (yellow), 40 °C and 90% RH air for 14 days (orange), and ambient air for 6 months (red).

To assess the stability of the adsorbent under humid air, TIFSIX-3-Ni powder (batch 3) was exposed to air environments of 40 °C and either 75% RH or 90% RH for 14 days, or to an ambient air environment for 6 months. We then characterized the samples’ crystallinity, textural properties, and CO_2_ adsorption and compared them to that of the as-synthesized sample, as shown in Figure 11. We measured XRD patterns of the samples immediately after their humidity treatment, *i.e.*, when there should still be adsorbed water in the sample, and after the same samples were degassed. We then measured N_2_ adsorption isotherms at −196 °C and CO_2_ adsorption isotherms at 25 °C on these degassed samples.

From Figure 11a, we see distinct changes in the XRD pattern of all three samples, both before and after degassing, compared to the as-synthesized sample. These changes are different than those we previously observed for the transition of the TIFSIX-3-Ni precursor to final TIFSIX-3-Ni powder (Figure 6). For the “before degas” samples, there are very noticeable changes including a left shift in many of the prominent peaks, such as those originally at 12° and 26°, the presence of additional peaks in the 15 to 20° range, and the disappearance of peaks in the 37 to 40° range. These changes in the XRD pattern suggest that water and/or oxygen is interacting with and perhaps inserting itself within the crystal structure of TIFSIX-3-Ni. Indeed, Barsoum et al. studied the structural changes in SIFSIX-3-Ni after exposure to 70 °C and 80% RH for 4 days, and reported that H_2_O was hydrogen bonding to SiF_6_^2–^ and forming coordination bonds with the Ni sites.^50^ After these samples are degassed, the XRD patterns are more comparable to the as-synthesized sample, however some crystallinity has been lost. Key changes include the broadening of remaining peaks, and the disappearance of peaks after ∼30°. The observed changes in the XRD pattern for the “after degas” sample exposed to 40 °C and 75% RH correspond well with those by Kumar et al.,^9^ who had subjected TIFSIX-3-Ni sample to 40 °C and 75% RH for 1, 7, and 14 days. Along with these structural changes, we observed color changes in the samples after degassing under vacuum at 160 °C as shown in Table S11, where the new color is somewhat reminiscent to that of NiTiF_6_ (Figure S5), further suggesting a change in the crystal structure/coordination. To understand this observation better, we performed XPS analysis on the sample exposed to ambient air for 6 months after it had been degassed. Compared to the as-synthesized sample, the oxygen content has almost doubled (Table S10), and there are noticeable shifts in the O 1*s* and Ti 2*p* core levels that may correspond to an increased presence of oxides of Ti (e.g., TiO_2_/TiOF_2_) (Figure S19). No distinct changes were observed for the C 1*s*, N 1*s*, F 1*s*, and Ni 2*p* spectra.

The N_2_ adsorption isotherms shown in Figure 11b and Figure S20b, indicate a severe decrease in porosity of all three samples compared to the as-synthesized sample, with a 70% to 80% decrease in BET areas, micropore volumes, and total pore volumes for all three samples (Table 6). Lastly, the CO_2_ isotherms shown in Figures 11c and 11d highlight significant reductions in the CO_2_ adsorption capacity at both 0.4 mbar and 1 bar for all three samples. At 0.4 mbar, we observe a 40% to 55% reduction in the CO_2_ uptake after exposure to all three conditions, with exposure to ambient air for 6 months having the most significant impact (Table 6). Our observations are not in alignment with the findings from Kumar et al.,^9^ who observed “negligible loss in terms of CO_2_ adsorption performance” after 14 days at 40 °C and 75% RH. However, Barsoum et al. also observed a drastic reduction in the CO_2_ uptake of SIFSIX-3-Ni after exposure to 70 °C and 80% RH, and suggested that SiF_6_^2–^ units were being displaced into the pores of SIFSIX-3-Ni which blocked the CO_2_ binding sites^50,9^

Overall, our findings indicate that TIFSIX-3-Ni would not be suitable for DAC processes implemented in tropical climates with warm temperatures and high humidity. Furthermore, TIFSIX-3-Ni would need to be replaced in a process at a minimum of once every 6 months, as exposure to ambient air for this period already results in more than a 50% decrease in CO_2_ adsorption capacity of the sample. This in turn would result in increased operational costs. In comparison, the lifetime of adsorbents currently used for other industrial separation processes, *e.g.*, zeolites, silicas, and activated carbons, is 5 to 10 years.^60^ However, given the differences in findings between our work and Kumar et al.,^9^ it is evident that further work is required to investigate the performance of TIFSIX-3-Ni in humid air, particularly around the interaction of water with the crystal structure of TIFSIX-3-Ni and its subsequent impacts. Preliminary investigations using ab initio calculations have recently been conducted by Ullah et al. to address this topic.^19^

#### 3.4.2 Cyclic Studies

**Figure 12:**
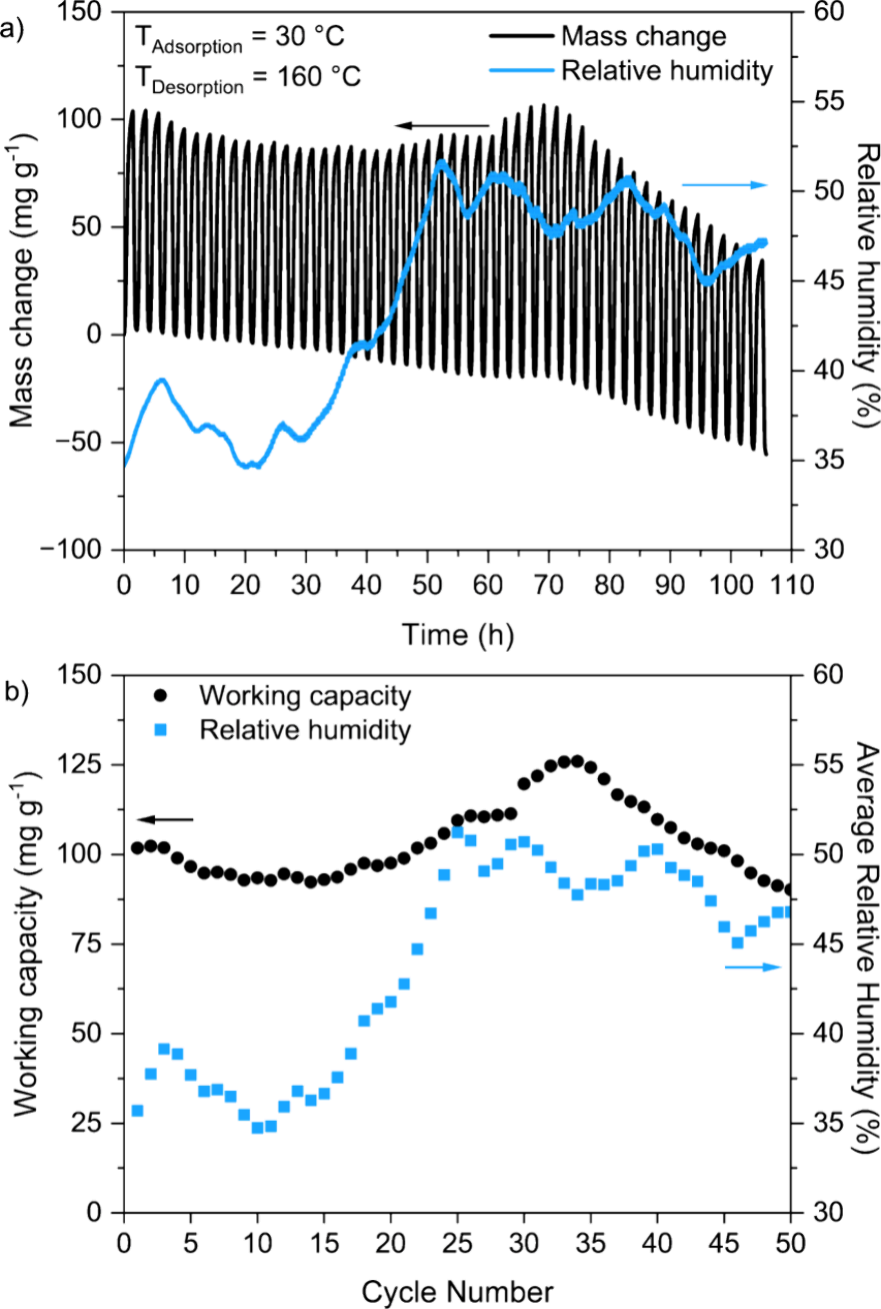
(a) Mass change of TIFSIX-3-Ni pellet (batch 5) per gram of activated sample (*i.e.*, sample mass just before the first cycle) over 50 adsorption–desorption cycles under a flow of ambient air with varying relative humidity. (b) Working capacity of the activated sample (*i.e.*, sample mass just before the first cycle) and average relative humidity of the ambient air over 50 cycles.

During a DAC process, an adsorbent will likely be subjected to multiple cycles of adsorption at ambient temperatures and desorption at elevated temperatures. The longer an adsorbent can maintain its CO_2_ adsorption performance under these conditions, the less frequently it will need to be replaced, leading to lower operation costs. Various desorption methods are currently considered for DAC, such as temperature swing, steam-assisted temperature swing, and temperature vacuum swing. Here, we investigated the stability of TIFSIX-3-Ni pellets (batch 5) under multiple cycles of a temperature swing adsorption process using ambient air with a CO_2_ concentration of ∼400 ppm. After an initial *in situ* activation of the sample at 160 °C under a mixed flow of ambient air (190 mL min^–1^) and protective He (10 mL min^–1^) to remove any preadsorbed gases, TIFSIX-3-Ni was subjected to 50 cycles of adsorption at 30 °C for 1 h and desorption at 160 °C for 15 min under the same mixed flow of ambient air and He with varying humidity (Figure 12a) using a thermogravimetric analyzer. Including the time needed to increase or decrease the temperature between 30 and 160 °C, the total time per cycle was 127 min. We chose 30 °C for the adsorption temperature as this was the minimum temperature which could be accurately maintained by the instrument. We chose 160 °C for the desorption temperature as this was the temperature used by Kumar et al.^9^ to degas the sorbent, and which we have subsequently applied for all our measurements thus far. We chose 1 h and 15 min for our adsorption and desorption times respectively, not including the time needed to reach the set temperatures, as the majority of adsorption (∼72%) and desorption (∼89%) could be achieved within this time based on our “long cycle” measurement shown in Figure S21 where we achieved saturation of the sample.

We calculated the resulting working capacity of each cycle shown below in Figure 12b, along with the average relative humidity during each cycle. Since we did not have a gas analyzer connected to our TGA to isolate the amount of CO_2_ adsorbed, we can only report the working capacity as an adsorbed mass per gram of activated sample. Over the first 15 cycles, there is a ∼ 10% decrease in the adsorbent’s working capacity. Then, over the next 20 cycles, there is a ∼ 40% increase in the working capacity. This increase correlates with the increasing relative humidity over these cycles, which would result in higher H_2_O adsorption and overall mass uptake by the sample. For the remaining 15 cycles, the relative humidity stays constant, but there is a ∼ 30% decrease in the working capacity. We link this change in the working capacity to the degradation of the sample. Indeed, when we repeated another “long cycle” on the same sample after cyclic testing, we observed a 36% decrease in its adsorption capacity under ambient air (Figure S22).

Our observations contrast with those reported by CSIRO, who observed no CO_2_ capacity loss in their Airthena DAC pilot-scale demonstrator based on a TIFSIX-3-Ni composite over 2680 cycles.^21^ CSIRO used similar adsorption and desorption times of 1 h and 30 min respectively, however their desorption was conducted under vacuum at 80 °C, and their air feed stream was predried to ∼3% RH at 25 °C. These differences, along with the presence of binder in the Airthena composite, could account for the enhanced stability observed by CSIRO. In comparison, in a separate study, Lewatit VP OC 1065 demonstrated ∼30% loss in its CO_2_ adsorption capacity after 180 cycles of adsorption in ambient air at 25% RH and desorption under vacuum at 80 °C.^61^

**Figure 13:**
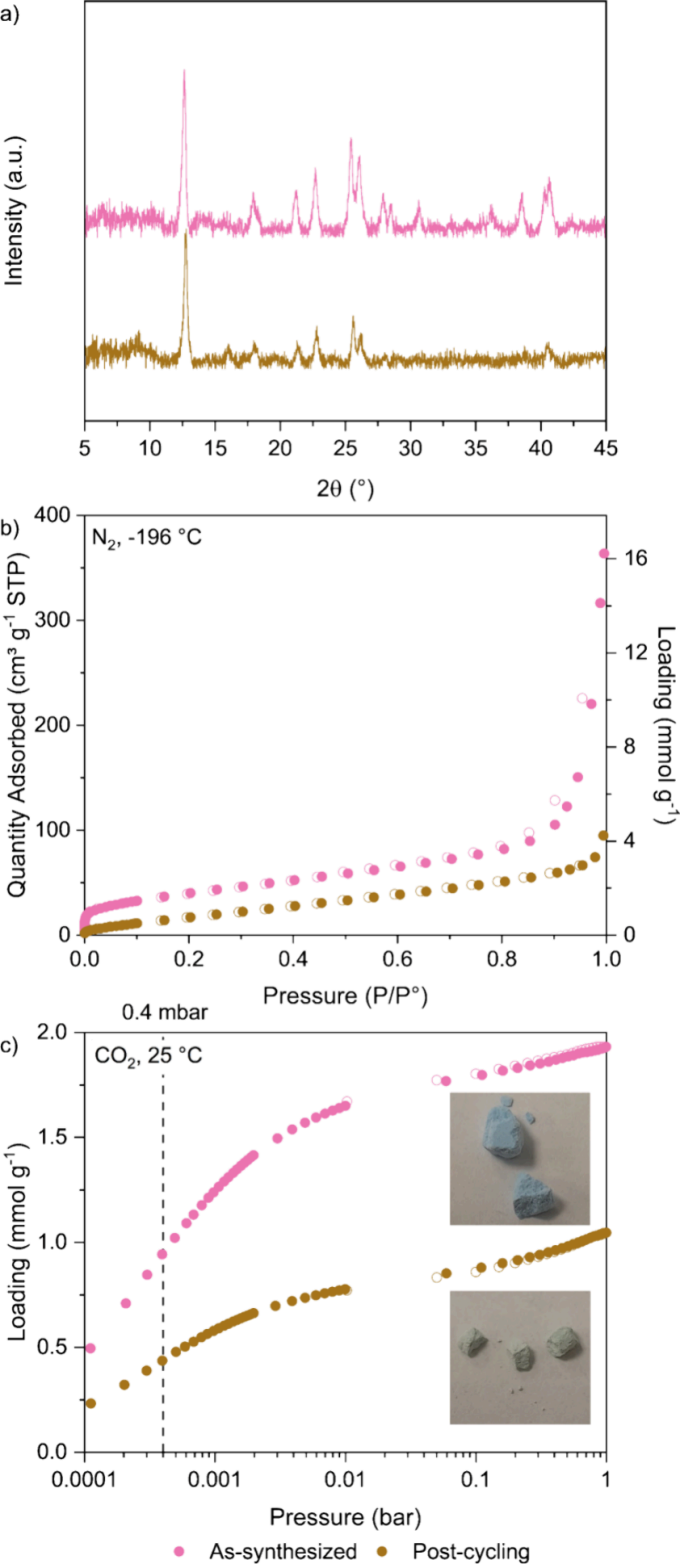
(a) XRD patterns, (b) N_2_ adsorption isotherms at −196 °C, and (c) CO_2_ adsorption isotherms at 25 °C with log-scale of pressure (filled symbols are adsorption; open symbols are desorption) for batch 5 TIFSIX-3-Ni pellet before (pink) and after postcyclic testing (bronze). Images of the sample before and after cycling are shown as insets in c).

We further characterized the postcyclic sample by comparing its crystallinity, textural properties, and CO_2_ adsorption to that of as-synthesized sample. From Figure 13a, peaks between 15 to 20° as well as after 26° have disappeared in the XRD pattern of postcyclic TIFSIX-3-Ni compared to as-synthesized TIFSIX-3-Ni, indicating some loss in crystallinity. The N_2_ adsorption data at −196 °C are shown in Figure 13b and Figure S20c with the textural parameters in Table 6. Here, we observe a ∼40% decrease in BET area and micropore volume, and ∼70% decrease in total pore volume. Lastly, as seen in Figure 13c, we observe a significant reduction in the CO_2_ adsorption capacity both at 1 bar and 0.4 mbar between the two samples. After 50 adsorption–desorption cycles, TIFSIX-3-Ni has suffered a ∼50% decrease in its CO_2_ adsorption capacity at 0.4 mbar. There is also a noticeable color change in the sample after cycling as shown in the insets in Figure 13c, which suggests a change in the crystal structure/coordination. Here, the sample has changed from a blue color to a color reminiscent of NiTiF_6_ (Figure S5).

We note that the BET area and CO_2_ adsorption at 0.4 mbar of the as-synthesized pellet samples reported in Table 6 (147 m^2^ g^–1^ and 0.94 mmol g^–1^) is different than those previously reported in Section 3.3.2 (106 m^2^ g^–1^ and 1.07 mmol g^–1^). This is likely due to the difference in sample mass used for the isotherm measurements, though we attempted to reduce this error by compensating with glass beads. Remeasuring the as-synthesized sample with the same methodology also allows a relative comparison between the samples.

Overall, TIFSIX-3-Ni would need to be replaced in a process after fewer than 50 cycles under these conditions, and its lifetime would not exceed more than 6 months based on our findings in Section 3.4.1.2. However, as shown by the CSIRO process,^20,21^ the cyclic stability of TIFSIX-3-Ni could be better than that of Lewatit VP OC 1065 by predrying the air feed stream, using a lower desorption temperature, and/or using an inert gas or vacuum for desorption. This could thus result in lower adsorbent costs compared to Lewatit VP OC 1065, though costs of manufacturing and raw chemicals would also need to be considered. In addition, reducing the desorption temperature would result in a lower working capacity, while the addition of a predrying step and use of inert gas or vacuum would likely increase the overall cost of the process. A technoeconomic assessment would therefore need to be conducted to accurately compare the cost of TIFSIX-3-Ni to other DAC adsorbents such as Lewatit VP OC 1065.

## 4 Conclusions

In this work, we aimed to thoroughly assess the potential of using TIFSIX-3-Ni as a DAC adsorbent by measuring as many of the material and equilibrium adsorption properties as needed to perform process scale modeling and optimization as possible, and investigating its shaping and stability under realistic process conditions.

We confirmed the successful synthesis of TIFSIX-3-Ni powder and reported its textural properties, thermal stability, and specific heat capacity. We also measured equilibrium adsorption isotherms of CO_2_, N_2_, Ar, O_2_, and H_2_O, and reported isotherm model fitting parameters and heats of adsorption for CO_2_, N_2_, and H_2_O. Compared to a benchmark DAC adsorbent, Lewatit VP OC 1065, TIFSIX-3-Ni may have higher gravimetric and volumetric CO_2_ adsorption capacity, but also higher N_2,_ Ar, O_2_, and H_2_O adsorption capacity. Therefore, the CO_2_ selectivity of TIFSIX-3-Ni is likely lower than that of Lewatit VP OC 1065. TIFSIX-3-Ni also has lower CO_2_ heat of adsorption compared to Lewatit VP OC 1065 and comparable N_2_ and H_2_O heats of adsorption, suggesting lower desorption energy requirements.

We easily scaled up the synthesis procedure of TIFSIX-3-Ni powder 4-fold but observed large variations in the textural properties and CO_2_ adsorption capacity between different batches. We also successfully pelletized TIFSIX-3-Ni powder with minimal loss in CO_2_ adsorption at 0.4 mbar, and we reported the skeletal, pellet, and bed density, total pore volume, and pellet porosity.

When subjected to humidity, TIFSIX-3-Ni is relatively stable at mild temperatures such as 25 °C, but it suffers a reduction in crystallinity, porosity, and CO_2_ adsorption at higher temperatures such as 40 °C, and there is a noticeable color change in the material. TIFSIX-3-Ni also has a relatively short lifetime, with again noticeable color changes and reduction in crystallinity, porosity, and CO_2_ adsorption after 50 sorption cycles in ambient air, or even storage in ambient air for 6 months. Future work still needs to be done, however, to gain a better fundamental understanding on the interaction of water with the crystal structure of TIFSIX-3-Ni and to further probe the possible structure flexibility under different conditions.

Overall, with this study, we hope we have provided the required data to facilitate process scale evaluation of TIFSIX-3-Ni for adsorption-based DAC, which can help continue the advancement and scale-up of this technology.

## Data Availability

The current version of the software package used for isotherm fitting and uncertainty calculation is available on the Imperial College London GitHub repository and can be accessed at https://github.com/ImperialCollegeLondon/IsothermFittingTool. The version of the code used in this publication is referred to by the git commit ID “a66f473”.

## Supporting Information Available

Additional experimental details and pictures of the setup and samples, XPS analysis of TIFSIX-3-Ni before and after exposure to humidity, additional isotherm fittings, heat of adsorption calculations, textural properties of TIFSIX-3-Ni pellets determined using CO_2_ adsorption at 0 °C, and *in situ* XRD analysis (PDF)
All experimentally measured data (isotherms, DSC, XRD, XPS, MIP, TGA) (ZIP)
