# Supplementary material for: Physicochemical Properties, Equilibrium Adsorption Performance, Manufacturability, and Stability of TIFSIX-3-Ni for Direct Air Capture of CO2
Source: Energy Fuels. 2024 Jun 18;38(13):11947–65. doi: 10.1021/acs.energyfuels.4c01368 (PMC11228916; doi:10.1021/acs.energyfuels.4c01368)
Supplement: Supplementary file 1 — ef4c01368_si_001.pdf [file ef4c01368_si_001.pdf]

# Supporting Information

# Physicochemical properties, equilibrium adsorption performance, manufacturability, and stability of TIFSIX-3-Ni for direct air capture of $CO_2$

*May-Yin (Ashlyn) Low*[1], *David Danaci*[1, 2, 3], *Hassan Azzan*[1], *Amanda Lim Jiayi*[1], *Gordon Wu Shun Yong*[1], *Ioanna Itskou*[1], *Camille Petit*[1*]

[1] Department of Chemical Engineering, Imperial College London, London SW7 2AZ, United Kingdom

[2]The Sargent Centre for Process Systems Engineering, Imperial College London, London SW7 2AZ, United Kingdom

[3]I-X Centre for AI in Science, Imperial College London, London W12 0BZ, United Kingdom

*Corresponding authors: camille.petit@imperial.ac.uk; d.danaci@imperial.ac.uk

# Table of Contents

1. Materials and methods ........................................................................................................3
2. Material properties ...............................................................................................................7
3. Adsorption properties ..........................................................................................................9
4. Manufacturing considerations.............................................................................................24
5. Adsorbent stability under process conditions .....................................................................29

## 1. Materials and methods

**Table S1.** Details of the chemicals used in this study.

| **Product name** | **Source** | **CAS** | **Product Code** | **Lot number** |
|---|---|---|---|---|
| Nickel (II) carbonate (anhydrous, 98%) | Alfa Aesar | 3333-67-3 | 22897 | M02G009 |
| Dihydrogen hexafluorotitanate | Alfa Aesar | 17439-11-1 | 39736 | X30F034 |
| Pyrazine | Fluorochem | 290-37-9 | 00275 | FCB111997 |
| Sodium chloride | VWR | 7747-14-5 | 27788 | - |
| Potassium nitrate | Sigma-Aldrich | 7757-79-1 | 221295-100G | MKCN5388 |

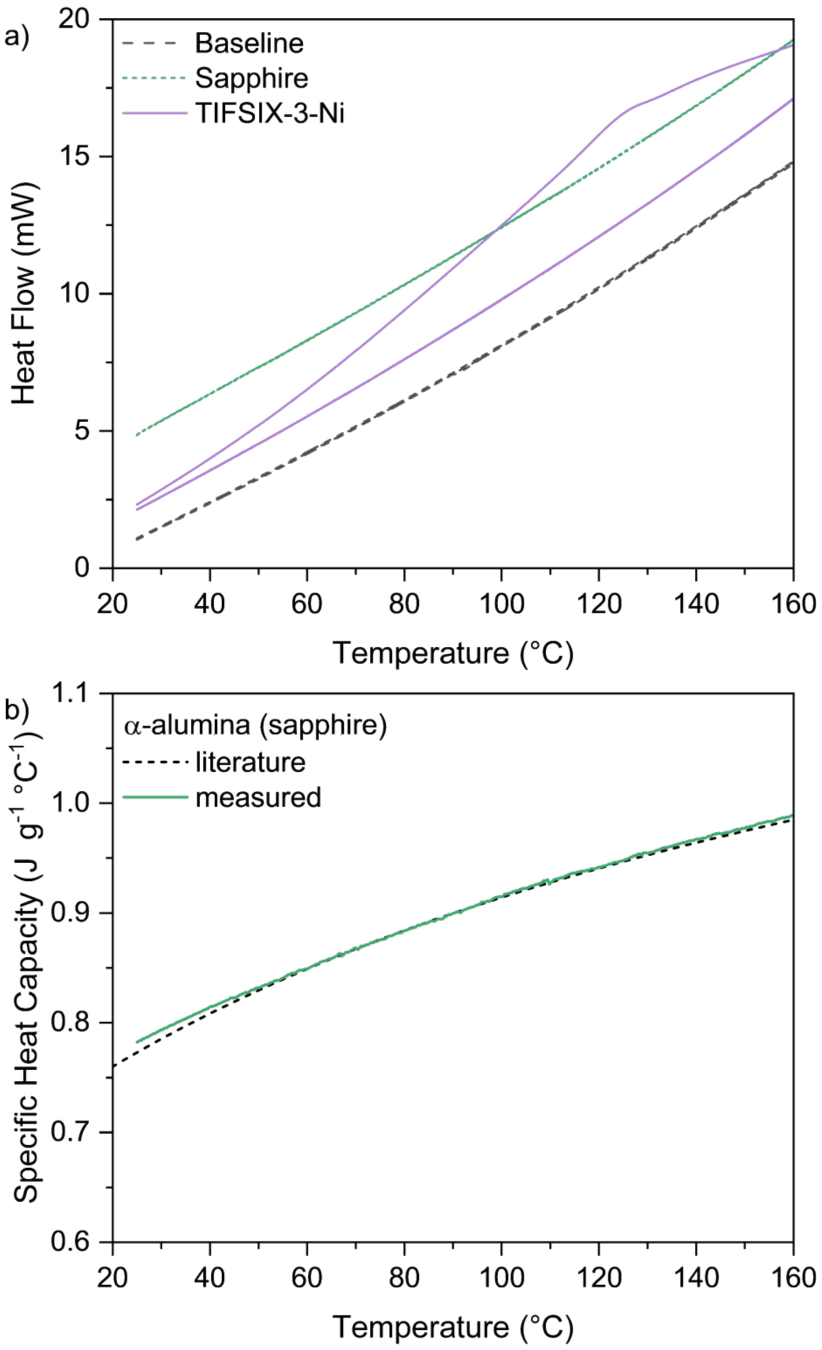


**Figure S1.** a) Heat flow curves of 3 baseline runs, 3 sapphire runs, and 4 sample runs for TIFSIX-3-Ni powder, where the first heat flow curve of the sample differs from the following three. b) Heat capacity of measured α-alumina disc provided by Perkin-Elmer, in comparison to literature values provided by NIST [1].

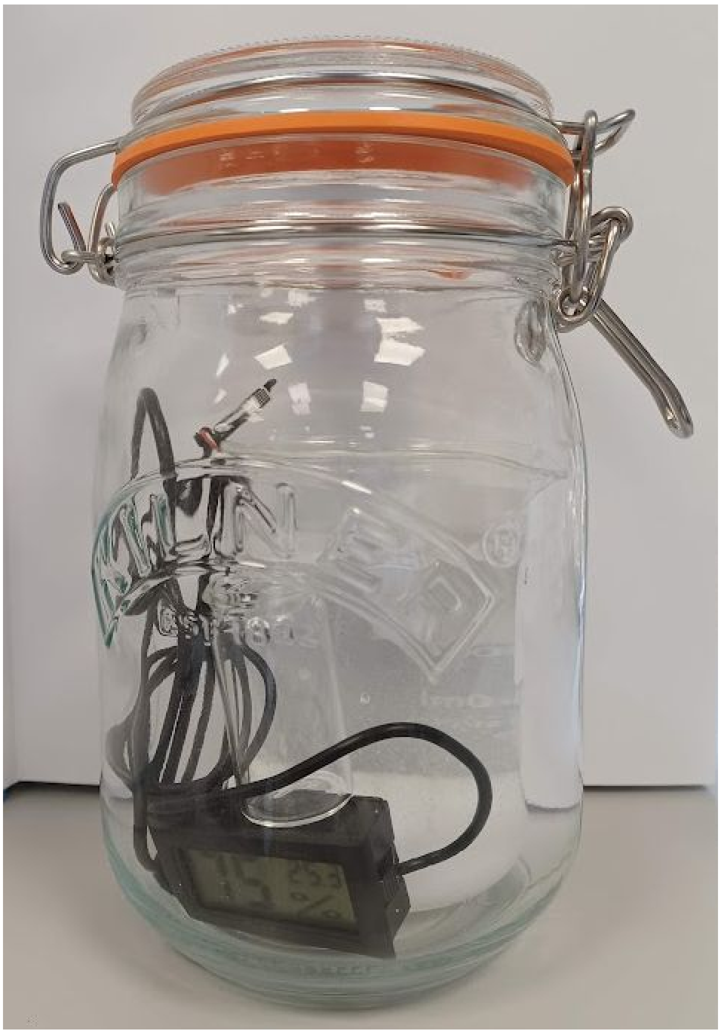

**Figure S2.** Image of the closed environment humidity setup. A 250 mL beaker filled with saturated salt solution of either sodium chloride or potassium nitrate is placed with a digital hygrometer-thermometer and an open 10 mL glass vial with TIFSIX-3-Ni sample (not shown in image) in a 1 L Kilner clip top mason jar with a rubber seal.

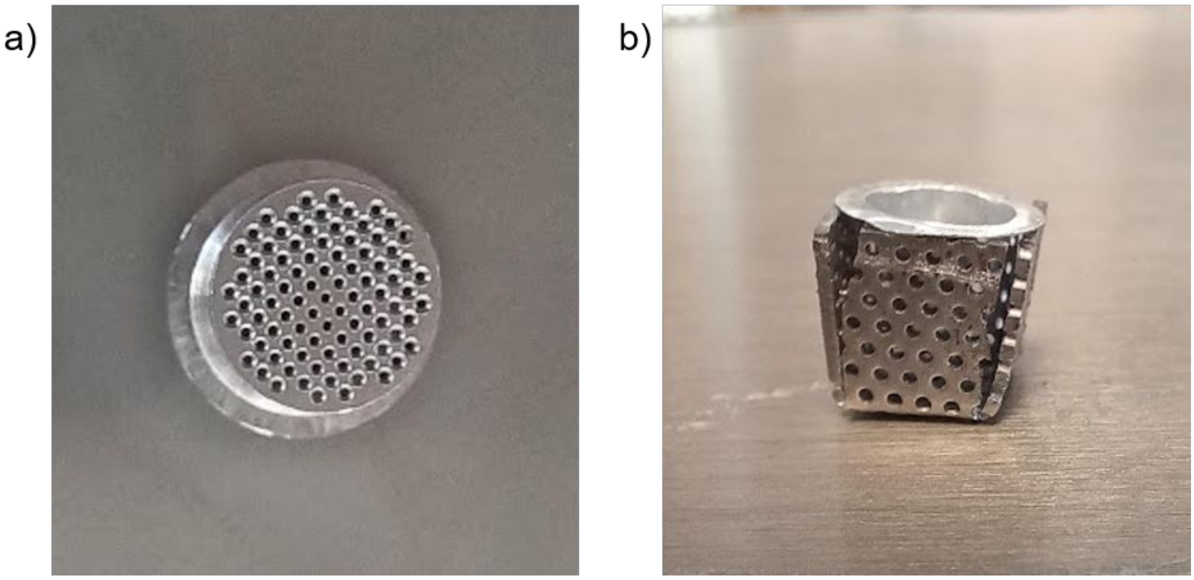


**Figure S3.** Image of a) aluminum crucible and b) its perforated stainless-steel holder used for our cyclic studies using a thermogravimetric analyzer.

## 2. Material properties

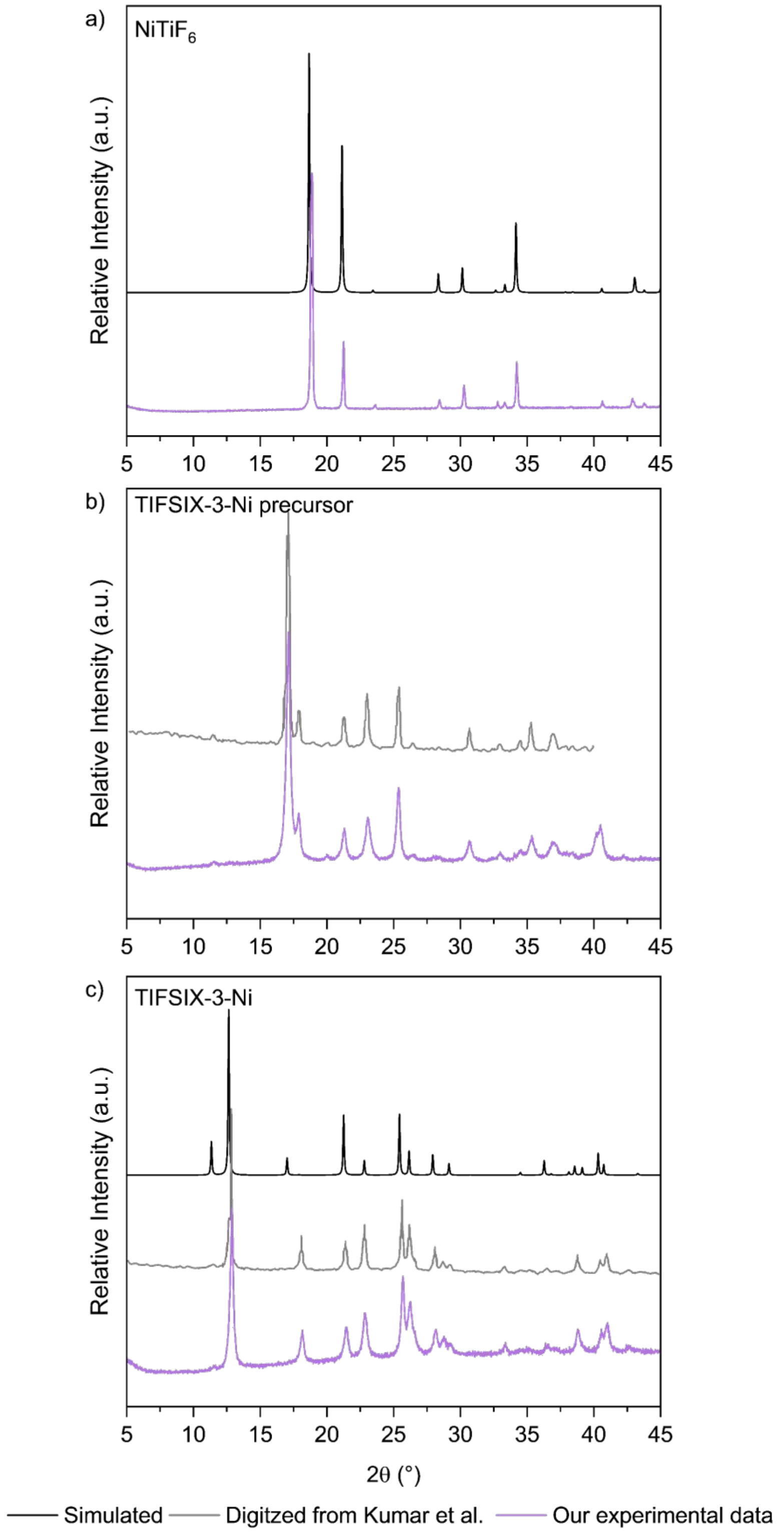


**Figure S4.** Comparison of measured XRD patterns to either simulated or digitized XRD patterns from literature for a) $NiTiF_6$, b) TIFSIX-3-Ni precursor, and c) TIFSIX-3-Ni. Simulated patterns are from the Cambridge Crystallographic Data Centre (CCDC) [2] ($NiTiF_6$: CCDC entry 404273 [3], TIFSIX-3-Ni: CCDC entry RIJGIE [4]) and digitized patterns are from Kumar et al. [4].

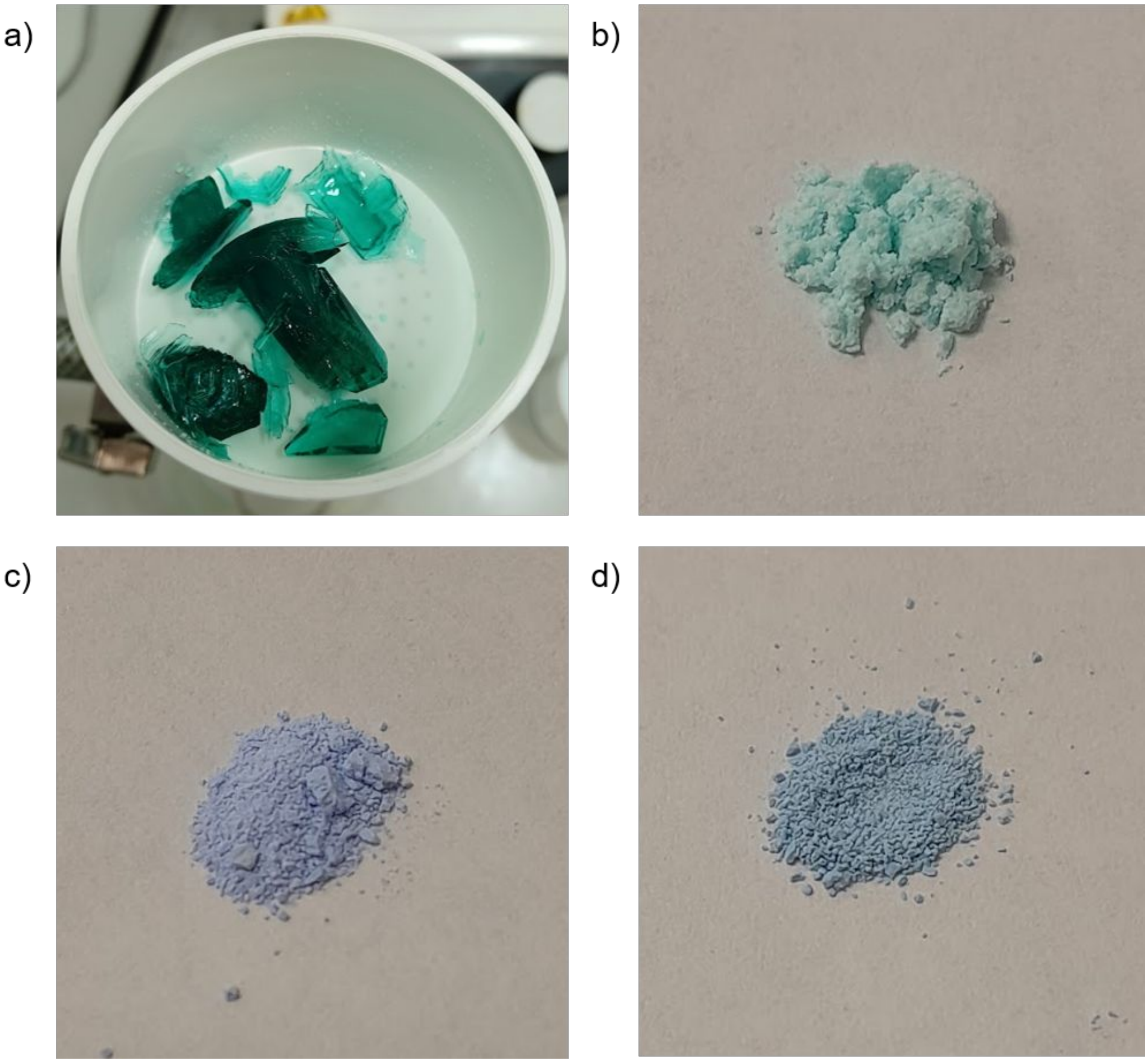


**Figure S5.** Images of a) as-synthesized $NiTiF_6$, b) ground $NiTiF_6$, c) TIFSIX-3-Ni precursor, and d) final TIFSIX-3-Ni powder.

## 3. Adsorption properties

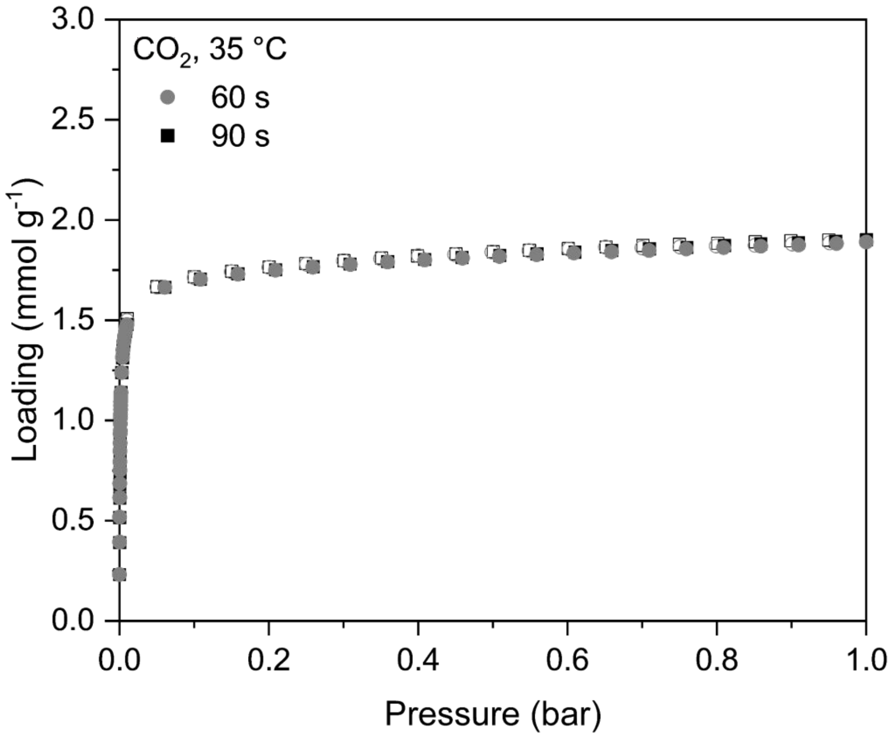


**Figure S6.** Equilibrium adsorption (filled symbols) and desorption (open symbols) isotherms of $CO_2$ at 35 °C for the same sample of TIFSIX-3-Ni with 60 s and 90 s equilibration intervals. Note that this is a different batch of TIFSIX-3-Ni (batch 1) from the one used to measure the $CO_2$ isotherms in the main study (batch 5).

**TIFSIX-3-Ni theoretical loading calculations:**

The following procedure was used to calculate the maximum theoretical $CO_2$ and $H_2O$ loadings in TIFSIX-3-Ni:

Molecular weight of unit cell [Ni $(C_4H_4N_2)_2$ $(TiF_6)$]: 381 g mol$^{-1}$ (CCDC entry RIJGIE [2, 4])

Avogadro's number: 6.022 x $10^{23}$ mol$^{-1}$

$$Mass\ of\ unit\ cell = \frac{381\ g\ mol^{-1}}{6.022\ \times\ 10^{23}} = 63.2\ \times 10^{-23} g$$

$$Number\ of\ unit\ cells\ in\ 1\ g = \frac{1}{63.2\ \times 10^{-23} g} = 0.0158\ \times 10^{23}\ g^{-1}$$

For $CO_2$, where one $CO_2$ molecule fits in a single unit cell cage:

Number of $CO_2$ molecules in 1 g = 0.0158 x $10^{23}$ g$^{-1}$

$$Moles\ of\ CO_2\ in\ 1\ g = \frac{0.0158\ \times 10^{23} g^{-1}}{6.022\ \times 10^{23} mol^{-1}} = 0.00263\ g\ mol^{-1} = 2.63\ g\ mmol^{-1}$$

For $H_2O$, where up to four $H_2O$ molecules fit in a single unit cell cage:

$$Number\ of\ H2O\ molecules\ in\ 1\ g\ = 4 * (0.0158\ \times 10^{23}\ g^{-1}) = 0.0633\ \times 10^{23} g^{-1}$$

$$Moles\ of\ H_2O\ in\ 1\ g = \frac{0.0633\ \times 10^{23} g^{-1}}{6.022\ \times 10^{23} mol^{-1}} = 0.0105\ g\ mol^{-1} = 10.5\ g\ mmol^{-1}$$

**Table S2.** Atomic composition and molar ratio (normalized to Ni) of TIFSIX-3-Ni determined by XPS.

| | **C** | **N** | **O** | **F** | **Ti** | **Ni** |
|---|---|---|---|---|---|---|
| Atomic composition (%) | 36.7 | 16.6 | 4.7 | 28.8 | 5.4 | 7.8 |
| Molar ratio (normalized to Ni) | 4.7 | 2.1 | 0.6 | 3.7 | 0.7 | 1.0 |

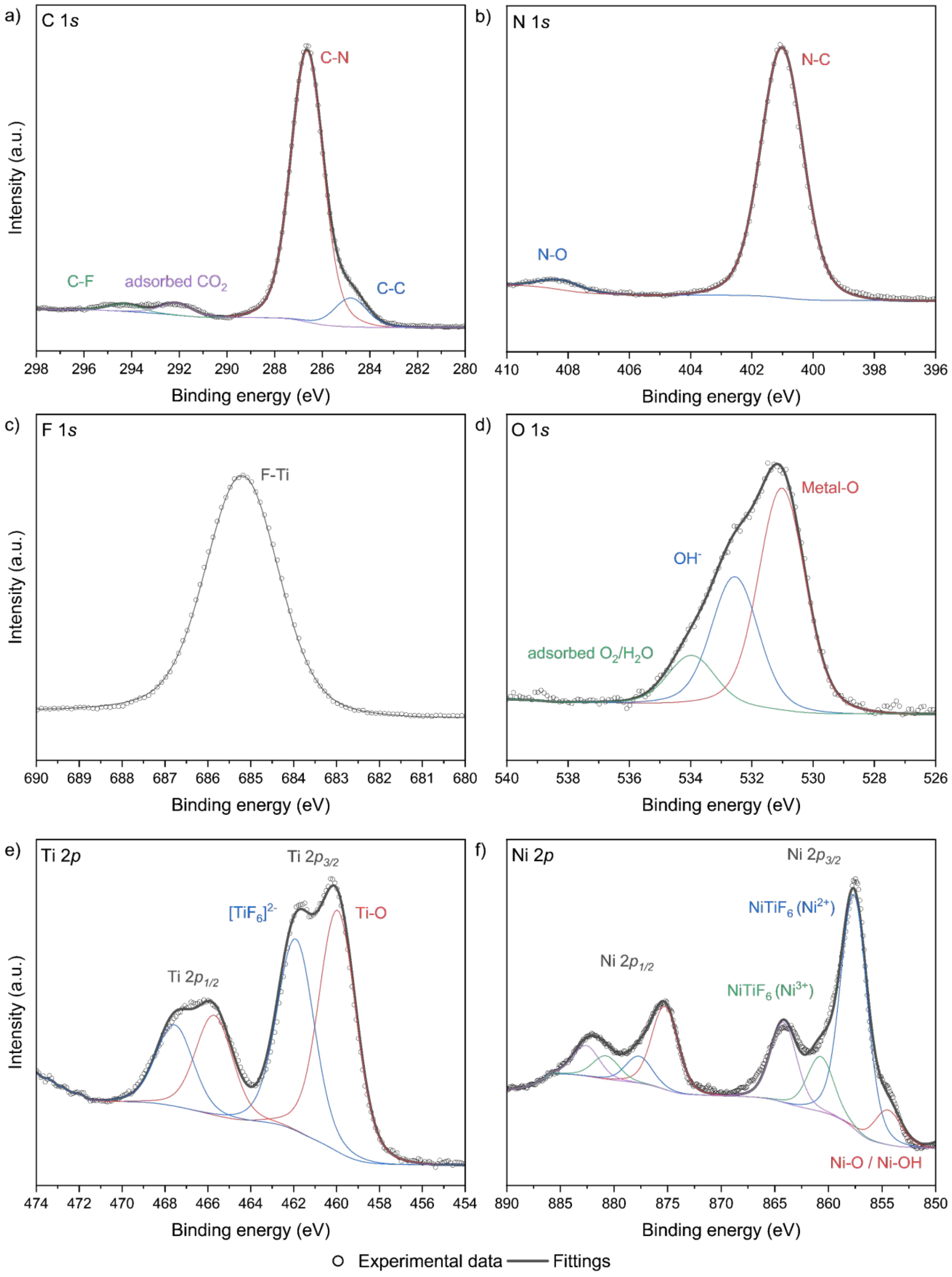


**Figure S7.** XPS data and peak fittings of the a) C 1*s*, b) N 1*s*, c) F 1*s*, d) O1*s*, e) Ti 2*p*, and f) Ni 2*p* core levels of TIFSIX-3-Ni. Peaks were assigned based on information from the NIST XPS Database [5] and the sample's structure [6].

**Table S3.** Upper and lower bounds imposed on fitting parameters for the DSL and SSL isotherm models.

| Fitting equation | Parameter | Units | Lower bound | Upper bound |
|---|---|---|---|---|
| DSL | $q_{s1}$ | mmol $g^{-1}$ | 0 | 30 |
| | $b_{0,1}$ | $bar^{-1}$ | 0 | 0.001 |
| | $-\Delta H_1$ | kJ $mol^{-1}$ | 0 | 100 |
| | $q_{s1}$ | mmol $g^{-1}$ | 0 | 30 |
| | $b_{0,2}$ | $bar^{-1}$ | 0 | 0.001 |
| | $-\Delta H_2$ | kJ $mol^{-1}$ | 0 | 100 |
| SSL | $q_s$ | mmol $g^{-1}$ | 0 | 30 |
| | $b_0$ | $bar^{-1}$ | 0 | 0.001 |
| | $-\Delta H$ | kJ $mol^{-1}$ | 0 | 100 |

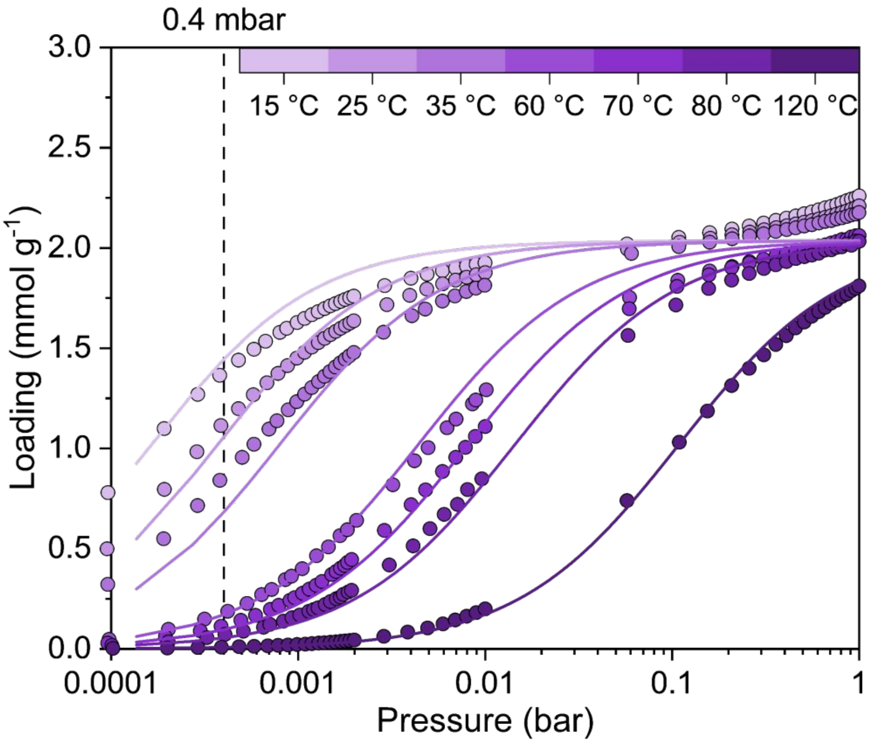


**Figure S8.** Equilibrium adsorption isotherms for $CO_2$, with a log-scale of pressure measured, at 15, 25, 35, 60, 70, 80, and 120 °C up to 1 bar for TIFSIX-3-Ni powder. Solid lines represent the fitting results from the SSL isotherm model, whose fitting parameters are found in Table S4.

**Table S4.** Fitting parameters with uncertainty bounds for a 95% confidence interval for the SSL isotherm model for $CO_2$ adsorption on TIFSIX-3-Ni.

| Adsorbate | Equation | Parameter | Unit | TIFSIX-3-Ni |
|---|---|---|---|---|
| $CO_2$ | SSL | $q_s$ | mmol $g^{-1}$ | 2.04 ± 0.02 |
| | | $b_0$ | ($\times 10^{-7}$) $bar^{-1}$ | 1.68 ± 0.08 |
| | | $-\Delta H$ | kJ $mol^{-1}$ | 58.3 ± 0.1 |

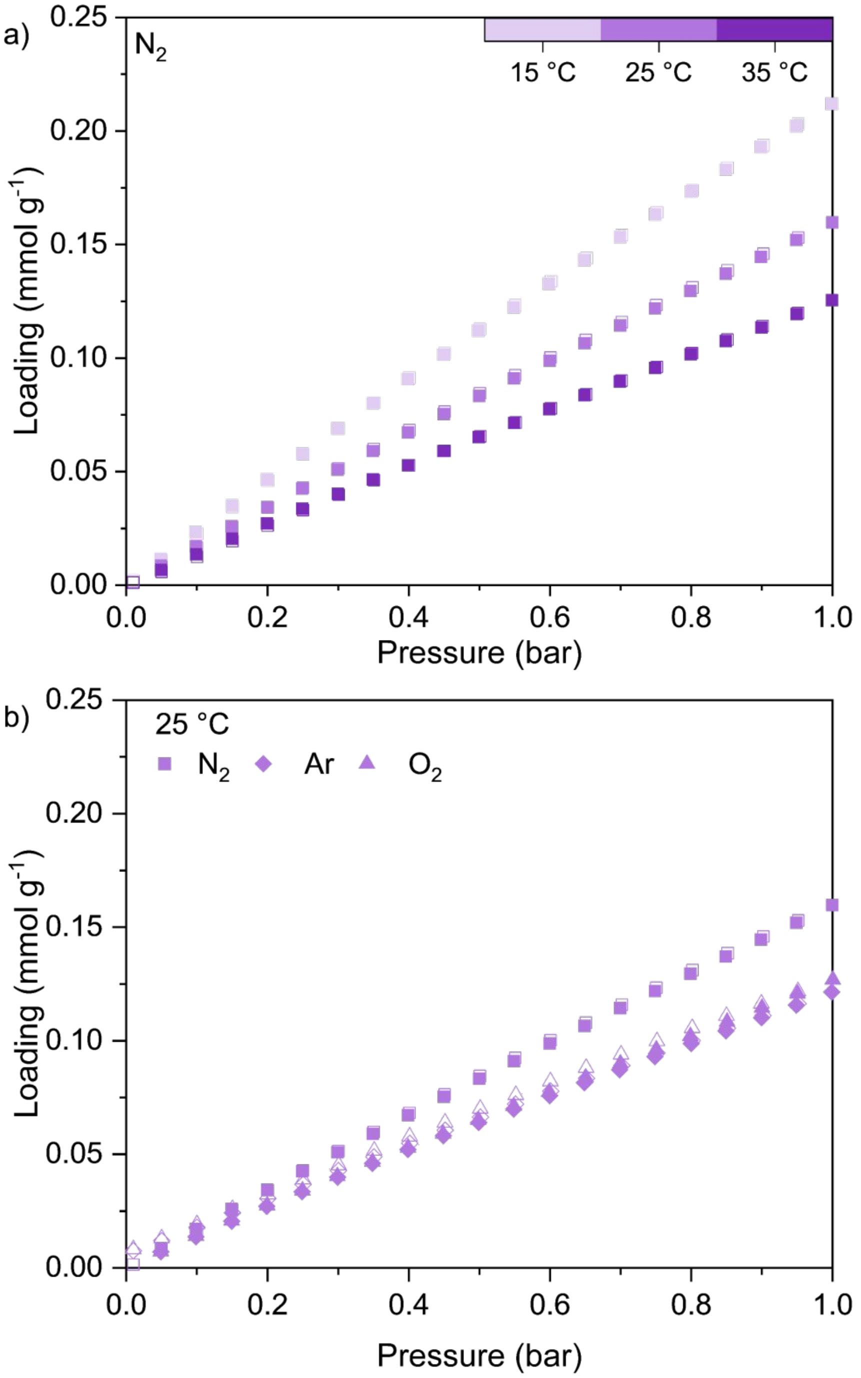


**Figure S9.** a) Equilibrium adsorption (filled symbols) and desorption (open symbols) isotherms for a) $N_2$ measured at 15, 25, and 35 °C up to 1 bar, and b) $N_2$, Ar, and $O_2$ measured at 25 °C up to 1 bar.

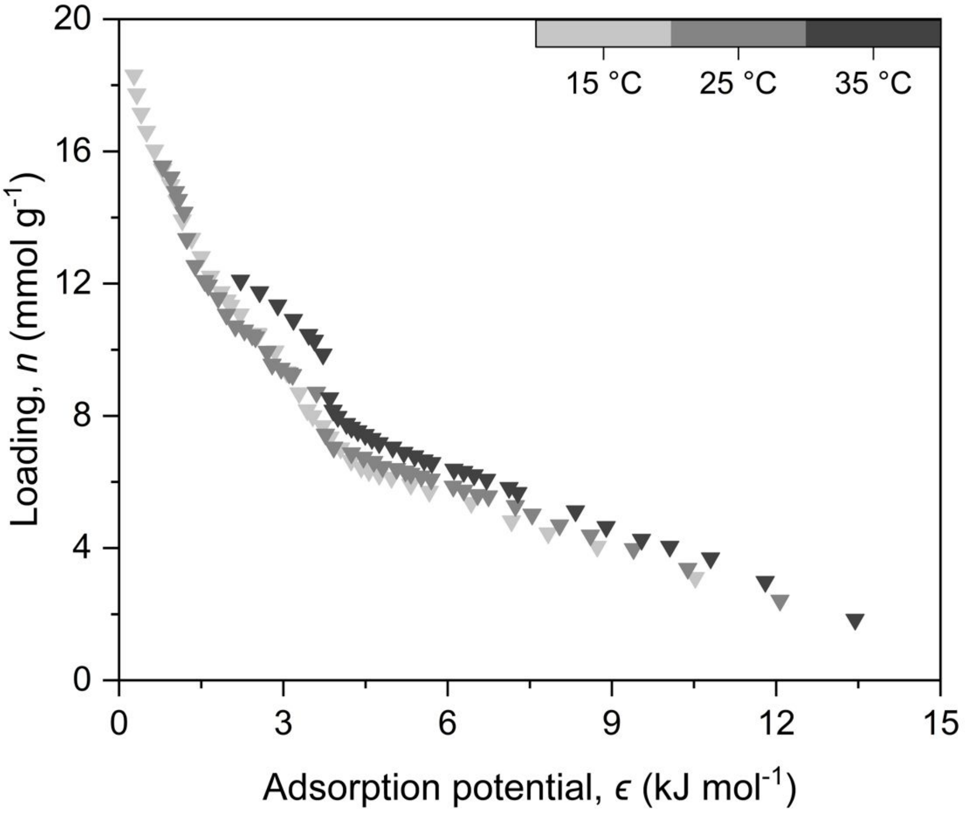


**Figure S10.** $H_2O$ adsorption potential for TIFSIX-3-Ni plotted against the $H_2O$ loading.

**Table S5.** Upper and lower bounds imposed on fitting parameters for the UNIV6 isotherm model. For this fitting procedure, the inverse of the adsorption equilibrium constant *K* was solved for and then converted to the final *K* value post-fitting. The bounds were also updated after every iteration with the initial and final bounds presented here. This was done as the magnitudes for the $\varepsilon$ parameters and correspondingly the *m* parameters can vary drastically. The same initial bounds are set for all $\varepsilon$ and *m* parameters and following the initial fitting, the upper and lower bounds are updated around the initial estimates (± 50%) and the parameter estimation is repeated by consequently updating the bounds, until the error between subsequent estimations is unchanged.

| **Parameter** | **Units** | **Initial bounds** | | **Final bounds** | |
|---|---|---|---|---|---|
| | | Lower bound | Upper bound | Lower bound | Upper bound |
| $q_s$ | mmol $g^{-1}$ | 0 | 100 | 18.301 | 119.28 |
| $\alpha_1$ | - | 0 | 1 | 0 | 1 |
| $\alpha_2$ | - | 0 | 1 | 0 | 1 |
| $\alpha_3$ | - | 0 | 1 | 0 | 1 |
| $\varepsilon_1$ | kJ $mol^{-1}$ | 0 | 0.0115129 | 0.21218 | 9688.0 |
| $\varepsilon_2$ | kJ $mol^{-1}$ | 0 | 0.0115129 | 0.18113 | 6041.1 |
| $\varepsilon_3$ | kJ $mol^{-1}$ | 0 | 0.0115129 | 0.19261 | 7257.8 |
| $\varepsilon_4$ | kJ $mol^{-1}$ | 0 | 0.0115129 | 0.17640 | 5583.3 |
| $m_1$ | kJ $mol^{-1}$ | 0 | 1 | 1.3451 | 4.0353 |
| $m_2$ | kJ $mol^{-1}$ | 0 | 1 | 0.29558 | 0.88675 |
| $m_3$ | kJ $mol^{-1}$ | 0 | 1 | 0.0028251 | 0.084754 |
| $m_4$ | kJ $mol^{-1}$ | 0 | 1 | 1.4323 | 4.2969 |
| $1/K$ | bar | 0 | 0.15 | 16.451 | $4.4603 \times 10^7$ |

**Table S6.** Fitting parameters with uncertainty bounds for a 95 % confidence interval for the UNIV6 isotherm model for $H_2O$ adsorption for TIFSIX-3-Ni determined using experimental isotherm data only at 15 and 25 °C. For this fitting procedure, the inverse of the adsorption equilibrium constant *K* was solved for and will need to be converted to the final *K* value post-fitting.

| **Parameter** | **Unit** | **TIFSIX-3-Ni** | **Initial bounds** | | **Final bounds** | |
|---|---|---|---|---|---|---|
| | | | Lower bound | Upper bound | Lower bound | Upper bound |
| $q_s$ | mmol $g^{-1}$ | 34.299 ± 0.4134 | 0 | 100 | 18.301 | 51.449 |
| $\alpha_1$ | - | 0.22737 ± 0.00745 | 0 | 1 | 0 | 1 |
| $\alpha_2$ | - | 0.69133 ± 0.00584 | 0 | 1 | 0 | 1 |
| $\alpha_3$ | - | 0.018041 ± 0.006288 | 0 | 1 | 0 | 1 |
| $\varepsilon_1$ | kJ $mol^{-1}$ | 51.397 ± 0.364 | 0 | 0.0115129 | 0.22571 | 11653 |
| $\varepsilon_2$ | kJ $mol^{-1}$ | 41.715 ± 0.051 | 0 | 0.0115129 | 0.20324 | 8520.2 |
| $\varepsilon_3$ | kJ $mol^{-1}$ | 43.267 ± 0.000 | 0 | 0.0115129 | 0.20701 | 9000.2 |
| $\varepsilon_4$ | kJ $mol^{-1}$ | 45.553 ± 0.198 | 0 | 0.0115129 | 0.21243 | 9722.8 |
| $m_1$ | kJ $mol^{-1}$ | 3.2648 ± 0.2670 | 0 | 1 | 1.6324 | 4.8972 |
| $m_2$ | kJ $mol^{-1}$ | 0.99795 ± 0.03845 | 0 | 1 | 0.49898 | 1.4969 |
| $m_3$ | kJ $mol^{-1}$ | 0.0014329 ± 0.0141271 | 0 | 1 | 0.00071643 | 0.0021493 |
| $m_4$ | kJ $mol^{-1}$ | 0.26840 ± 0.16651 | 0 | 1 | 0.13420 | 0.40260 |
| *1/K* | bar | $7.4650 \times 10^5 \pm 0.1120 \times 10^5$ | 0 | 0.15 | 86.390 | $6.4498 \times 10^9$ |

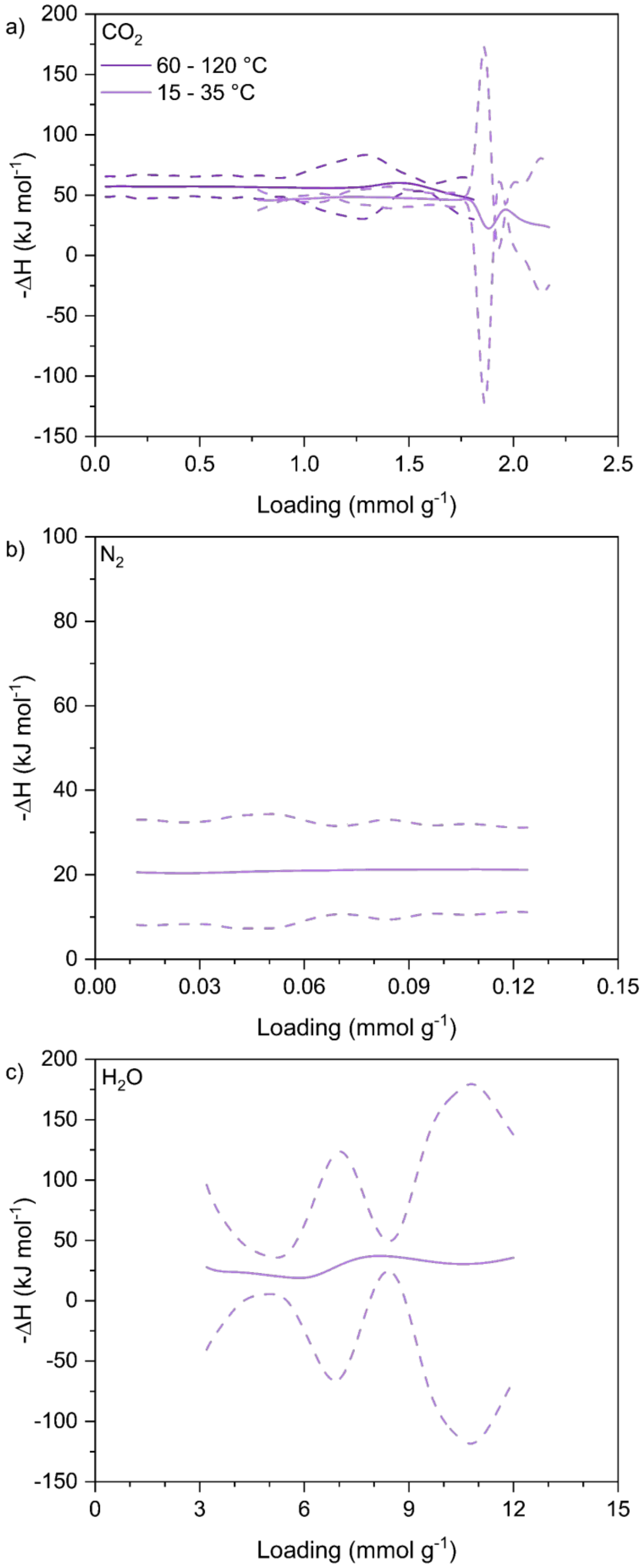


**Figure S11.** a) Isosteric heats of adsorption (solid lines) for a) $CO_2$ calculated using isotherm data measured at 60, 70, 80 and 120 °C, and 15, 25, and 35 °C, and for b) $N_2$ and c) $H_2O$, with corresponding upper and lower confidence intervals (dashed lines).

**Table S7.** Smoothing spline values used in MATLAB for the interpolation of $CO_2$, $N_2$, and $H_2O$ data to calculate the corresponding isosteric heats of adsorption for TIFSIX-3-Ni.

| **Adsorbate** | **Smoothing values** |
| --- | --- |
| $CO_2$ | 0.999995 |
| $N_2$ | 0.999999 |
| $H_2O$ | 0.95 |

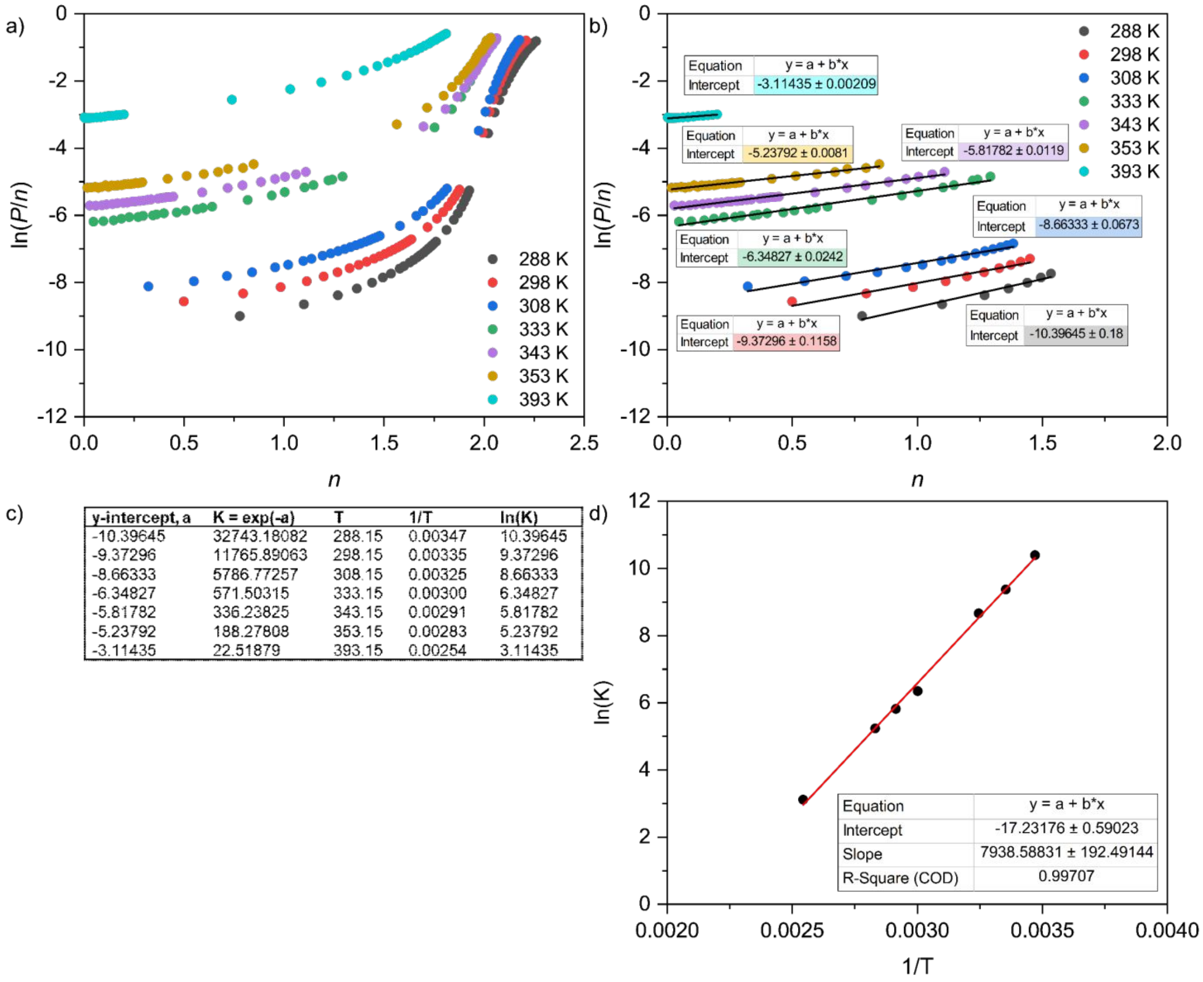


| y-intercept, a | K = exp(-a) | T | 1/T | ln(K) |
|---|---|---|---|---|
| -10.39645 | 32743.18082 | 288.15 | 0.00347 | 10.39645 |
| -9.37296 | 11765.89063 | 298.15 | 0.00335 | 9.37296 |
| -8.66333 | 5786.77257 | 308.15 | 0.00325 | 8.66333 |
| -6.34827 | 571.50315 | 333.15 | 0.00300 | 6.34827 |
| -5.81782 | 336.23825 | 343.15 | 0.00291 | 5.81782 |
| -5.23792 | 188.27808 | 353.15 | 0.00283 | 5.23792 |
| -3.11435 | 22.51879 | 393.15 | 0.00254 | 3.11435 |

**Figure S12.** a) Virial plot of all measured $CO_2$ isotherm data for TIFSIX-3-Ni, where pressure (*P*), loading (*n*), and temperature (*T*) values are in units of bar, mmol $g^{-1}$, and Kelvin, respectively. b) Virial plot showing the selected low loading data fitted to linear equations. The y-intercepts of each fitted equation are used to calculate the Henry constant K. c) Summary of fitted and calculated parameters used to determine $\Delta H_0$. d) Plot of lnK vs. 1/T fitted to a linear equation, whose slope is used to calculate $\Delta H_0$.

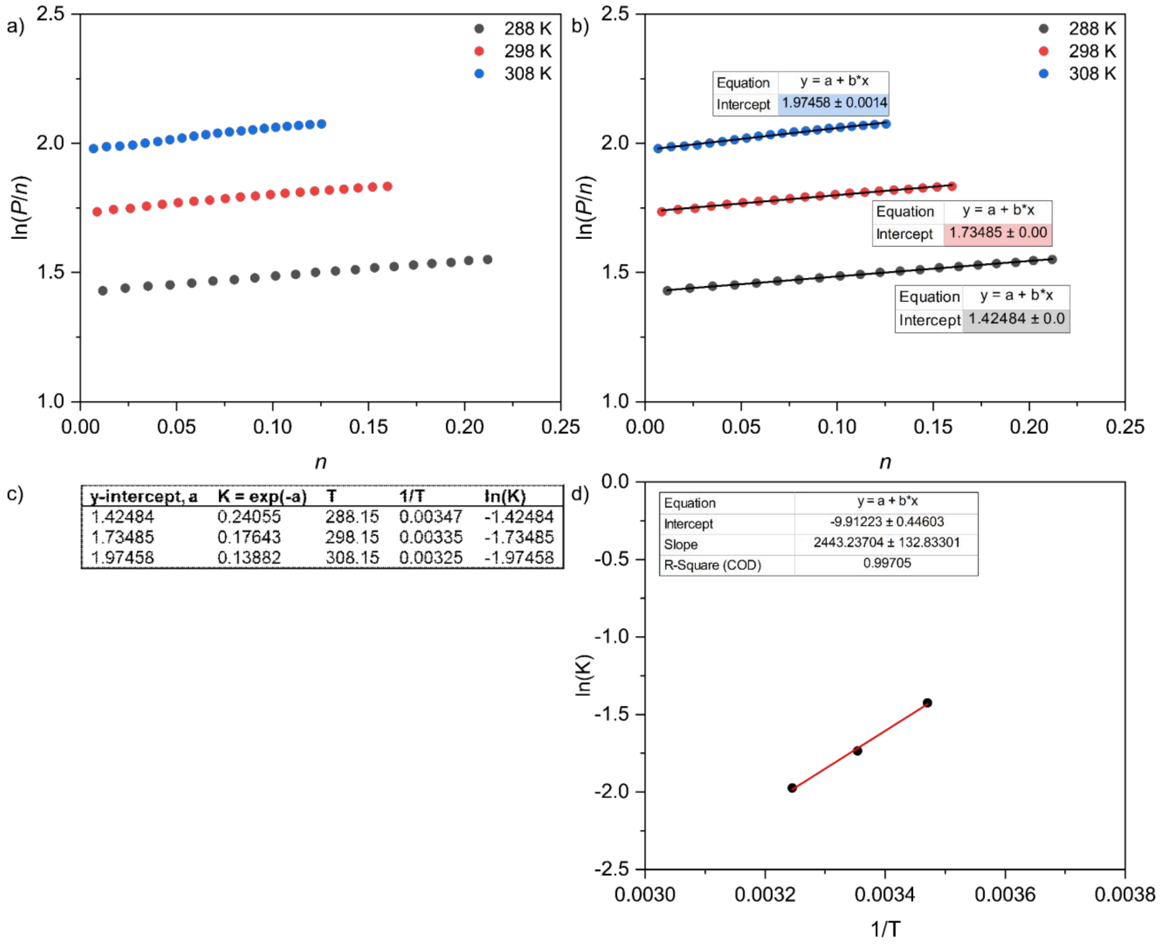


| y-intercept, a | K = exp(-a) | T | 1/T | ln(K) |
|---|---|---|---|---|
| 1.42484 | 0.24055 | 288.15 | 0.00347 | -1.42484 |
| 1.73485 | 0.17643 | 298.15 | 0.00335 | -1.73485 |
| 1.97458 | 0.13882 | 308.15 | 0.00325 | -1.97458 |

**Figure S13.** a) Virial plot of all measured $N_2$ isotherm data for TIFSIX-3-Ni, where pressure (*P*), loading (*n*), and temperature (*T*) values are in units of bar, mmol $g^{-1}$, and Kelvin, respectively. b) Virial plot showing the selected low loading data fitted to linear equations. The y-intercepts of each fitted equation are used to calculate the Henry constant K. c) Summary of fitted and calculated parameters used to determine $\Delta H_0$. d) Plot of lnK vs. 1/T fitted to a linear equation, whose slope is used to calculate $\Delta H_0$.

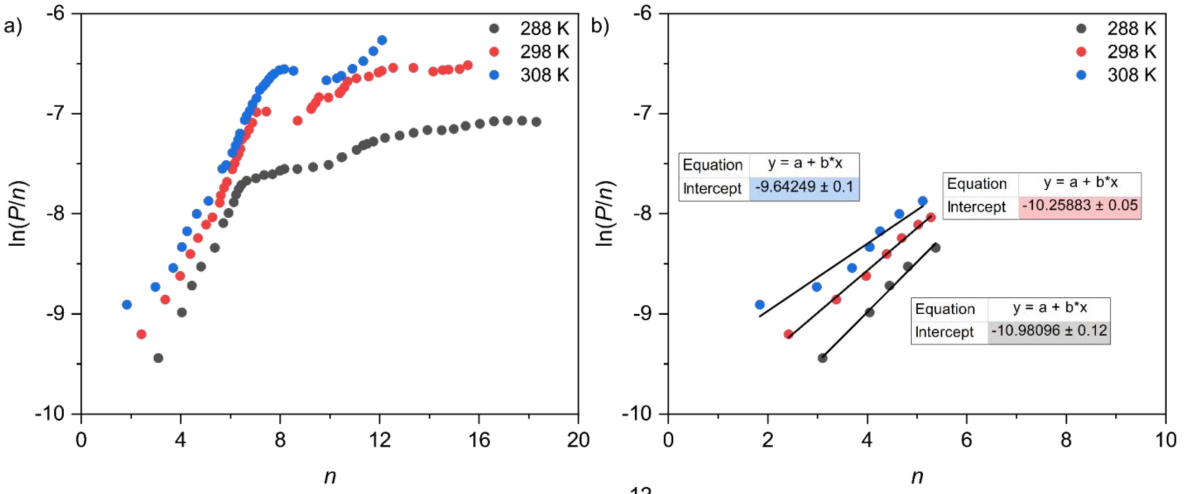


c)

| y-intercept, a | K = exp(-a) | T | 1/T | ln(K) |
|---|---|---|---|---|
| -10.98096 | 58744.92234 | 288.25 | 0.00347 | 10.98096 |
| -10.25883 | 28533.3826 | 298.15 | 0.00335 | 10.25883 |
| -9.64249 | 15405.6561 | 308.15 | 0.00325 | 9.64249 |

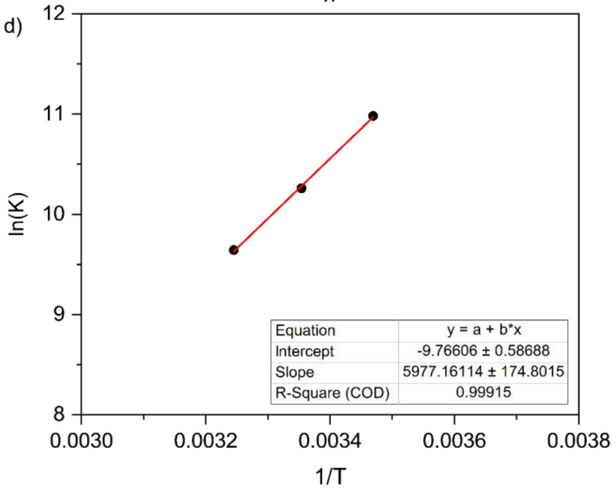


**Figure S14.** a) Virial plot of all measured $H_2O$ isotherm data for TIFSIX-3-Ni, where pressure (*P*), loading (*n*), and temperature (*T*) values are in units of bar, mmol $g^{-1}$, and Kelvin, respectively. b) Virial plot showing the selected low loading data fitted to linear equations. The y-intercepts of each fitted equation are used to calculate the Henry constant K. c) Summary of fitted and calculated parameters used to determine $\Delta H_0$. d) Plot of lnK vs. 1/T fitted to a linear equation, whose slope is used to calculate $\Delta H_0$.

## 4. Manufacturing considerations

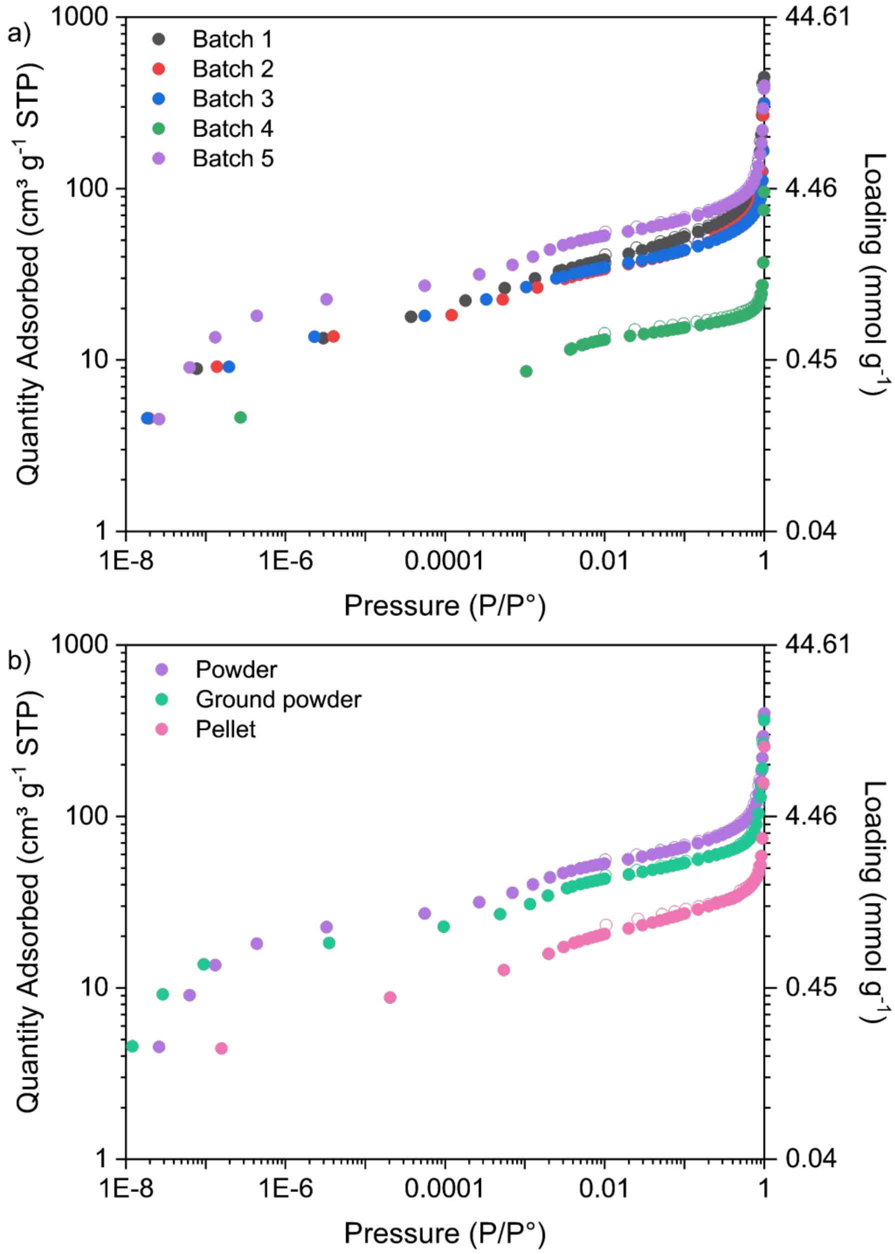


**Figure S15.** $N_2$ adsorption (filled symbols) and desorption (open symbols) isotherms at -196 °C with log-scale of pressure and loading for a) different batches of TIFSIX-3-Ni and b) TIFSIX-3-Ni powder, ground powder, and pellet (batch 5).

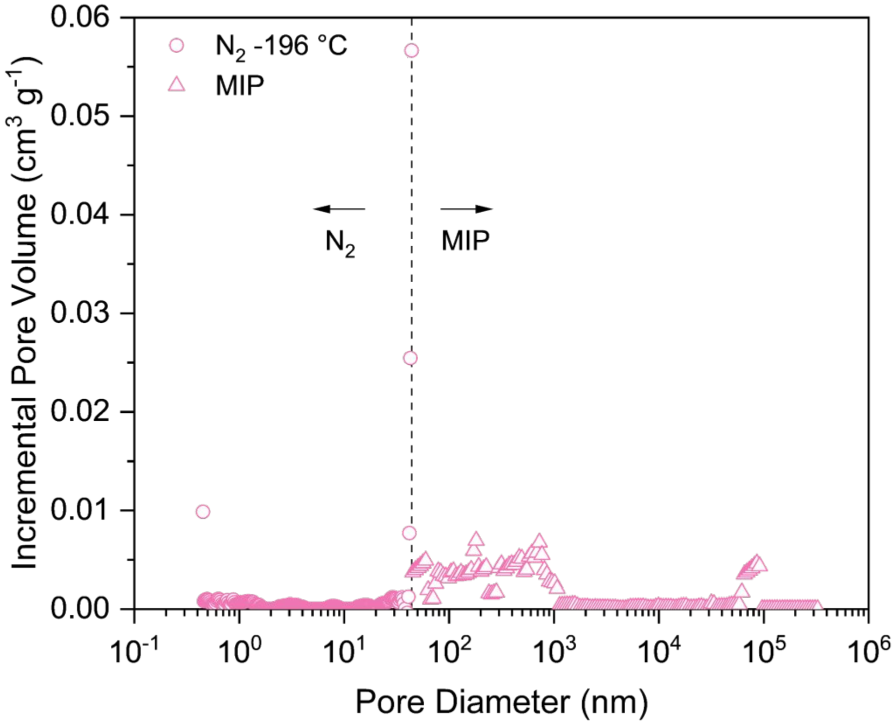


**Figure S16.** Incremental pore volume as a function of pore diameter of TIFSIX-3-Ni pellet, obtained from $N_2$ sorption at -196 °C (circles) and mercury intrusion porosimetry (triangles).

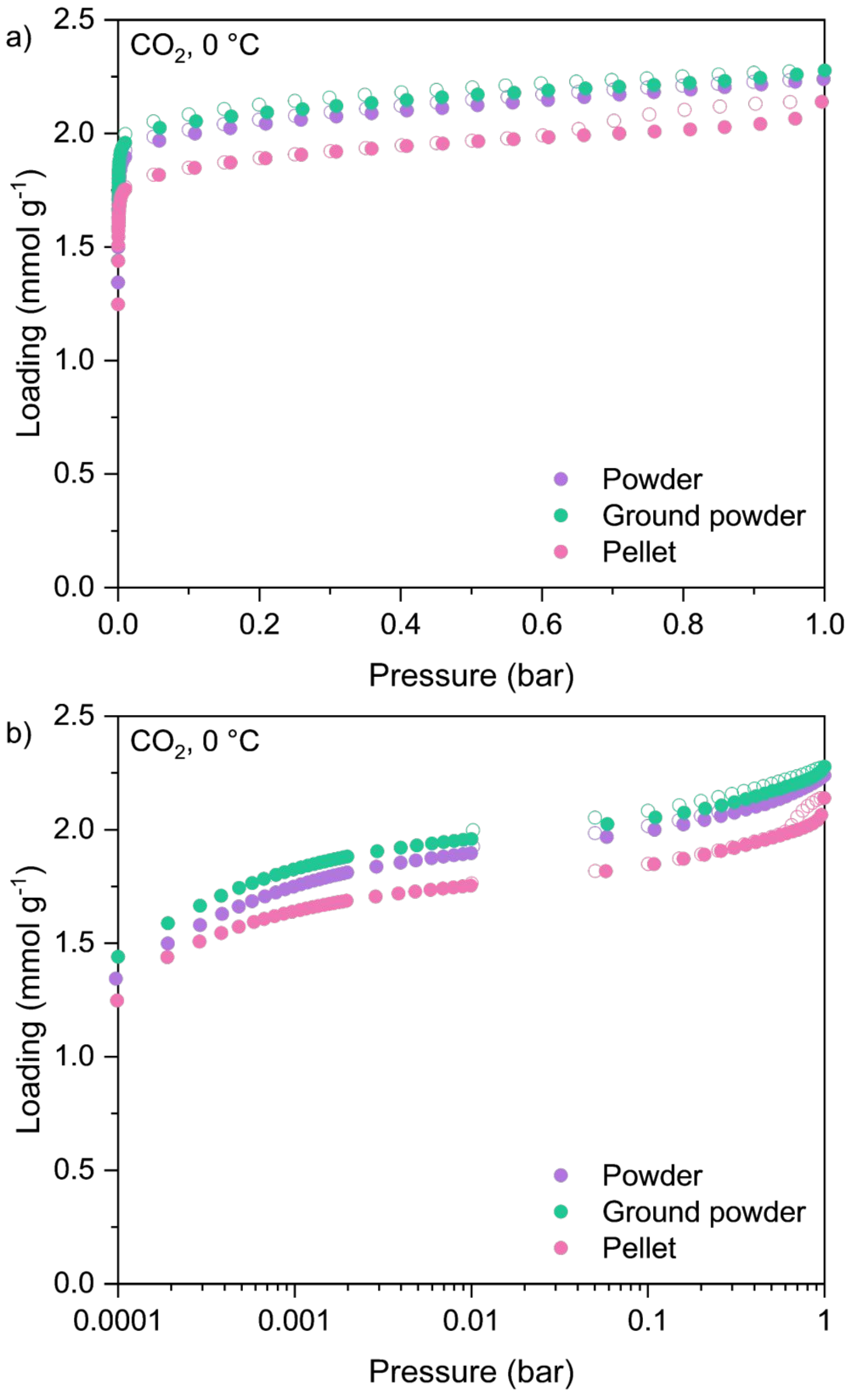


**Figure S17.** $CO_2$ adsorption (filled symbols) and desorption (open symbols) isotherms at 0 °C with a) linear and b) log-scale of pressure for batch 5 TIFSIX-3-Ni powder, ground powder, and pellet.

**Table S8.** Comparison of textural properties of batch 5 TIFSIX-3-Ni powder, ground powder, and pellet determined using $N_2$ adsorption isotherms measured at -196 °C and $CO_2$ adsorption isotherms measured at 0 °C.

| **TIFSIX-3-Ni** | **$N_2$ -196 °C** | | | **$CO_2$ 0 °C** | | |
|---|---|---|---|---|---|---|
| | **$S_{BET}$ ($m^2$ $g^{-1}$)** | **$V_{micro}$ ($cm^3$ $g^{-1}$)** | **$V_{total}$ ($cm^3$ $g^{-1}$)** | **$S_{BET}$ ($m^2$ $g^{-1}$)** | **$V_{micro}$ ($cm^3$ $g^{-1}$)** | **$V_{total}$ ($cm^3$ $g^{-1}$)** |
| Powder | 264 | 0.11 | 0.50 | 183 | 0.10 | - |
| Ground powder | 213 | 0.09 | 0.41 | 186 | 0.10 | - |
| Pellet | 106 | 0.06 | 0.24 | 170 | 0.09 | - |

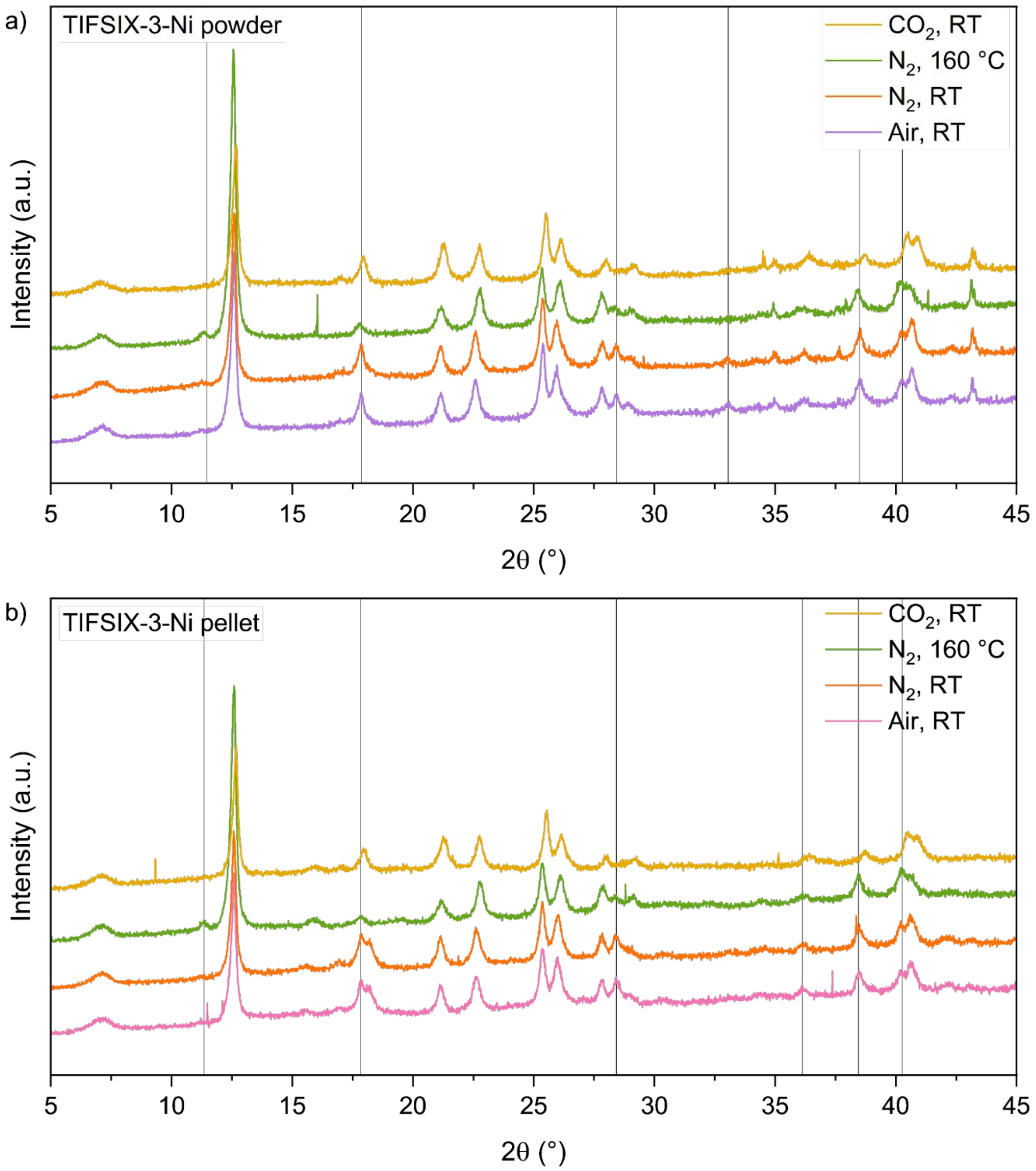


**Figure S18.** In-situ XRD patterns of batch 5 TIFSIX-3-Ni a) powder and b) pellet under different gas flows and temperatures. Vertical lines are included to help visualize the changes in the patterns.

## 5. Adsorbent stability under process conditions

**Table S9.** Images of the colors of batch 5 TIFSIX-3-Ni powder samples after exposure to different humidity conditions and subsequent characterizations.

| | | **1** | **2** | **3** |
|---|---|---|---|---|
| | Situation | Initial appearance | Appearance after ex-situ degas at 160 °C | Appearance after $CO_2$ 25 °C isotherm measurement |
| **A** | As synthesized (batch 5) | 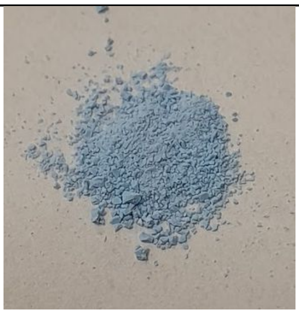 | 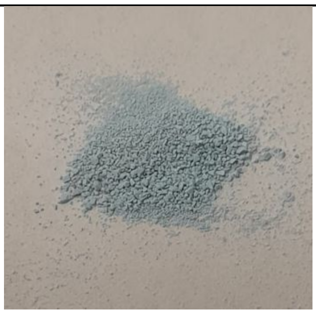 | 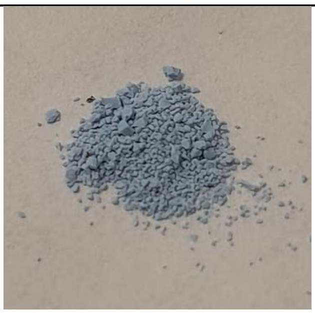 |
| **B** | Exposure to $H_2O$ (25 °C, 15% RH) | Similar to C1 | Similar to A2 | 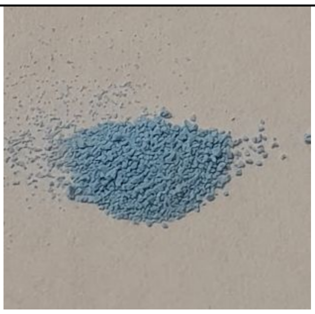 |
| **C** | Exposure to $H_2O$ (25 °C, 48% RH) | 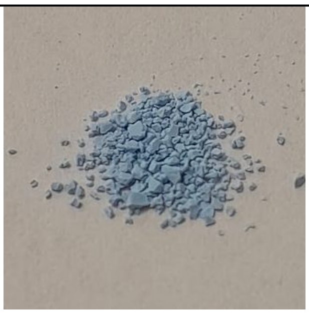 | Similar to A2 | 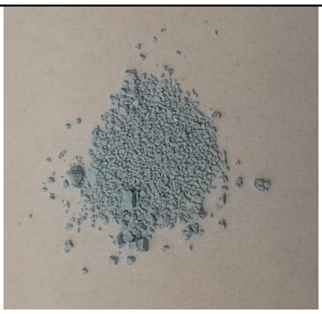 |
| **D** | Exposure to $H_2O$ (25 °C, 77% RH) | Similar to C1 | Similar to A2 | 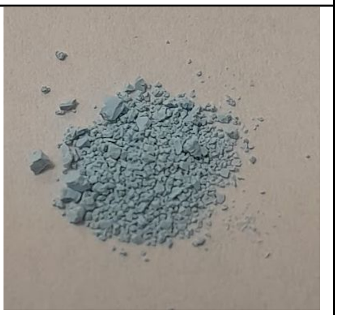 |

**Table S10.** Atomic composition and molar ratio (normalized to Ni) of TIFSIX-3-Ni samples (as synthesized, after $H_2O$ exposure, and after air exposure) determined by XPS.

| | **TIFSIX-3-Ni sample** | **C** | **N** | **O** | **F** | **Ti** | **Ni** |
|---|---|---|---|---|---|---|---|
| **Atomic composition (%)** | As-synthesized | 36.7 | 16.6 | 4.7 | 28.8 | 5.4 | 7.8 |
| | After exposure to $H_2O$ (25 °C, 77% RH) | 32.4 | 14.5 | 5.8 | 32.7 | 6.1 | 8.6 |
| | After exposure to ambient air (6 months) | 29.8 | 13 | 8.7 | 32.4 | 7.1 | 8.9 |
| **Molar ratio (normalized to Ni)** | As-synthesized | 4.7 | 2.1 | 0.6 | 3.7 | 0.7 | 1.0 |
| | After exposure to $H_2O$ (25 °C, 77% RH) | 3.8 | 1.7 | 0.7 | 3.8 | 0.7 | 1.0 |
| | After exposure to ambient air (6 months) | 3.3 | 1.5 | 1.0 | 3.6 | 0.8 | 1.0 |

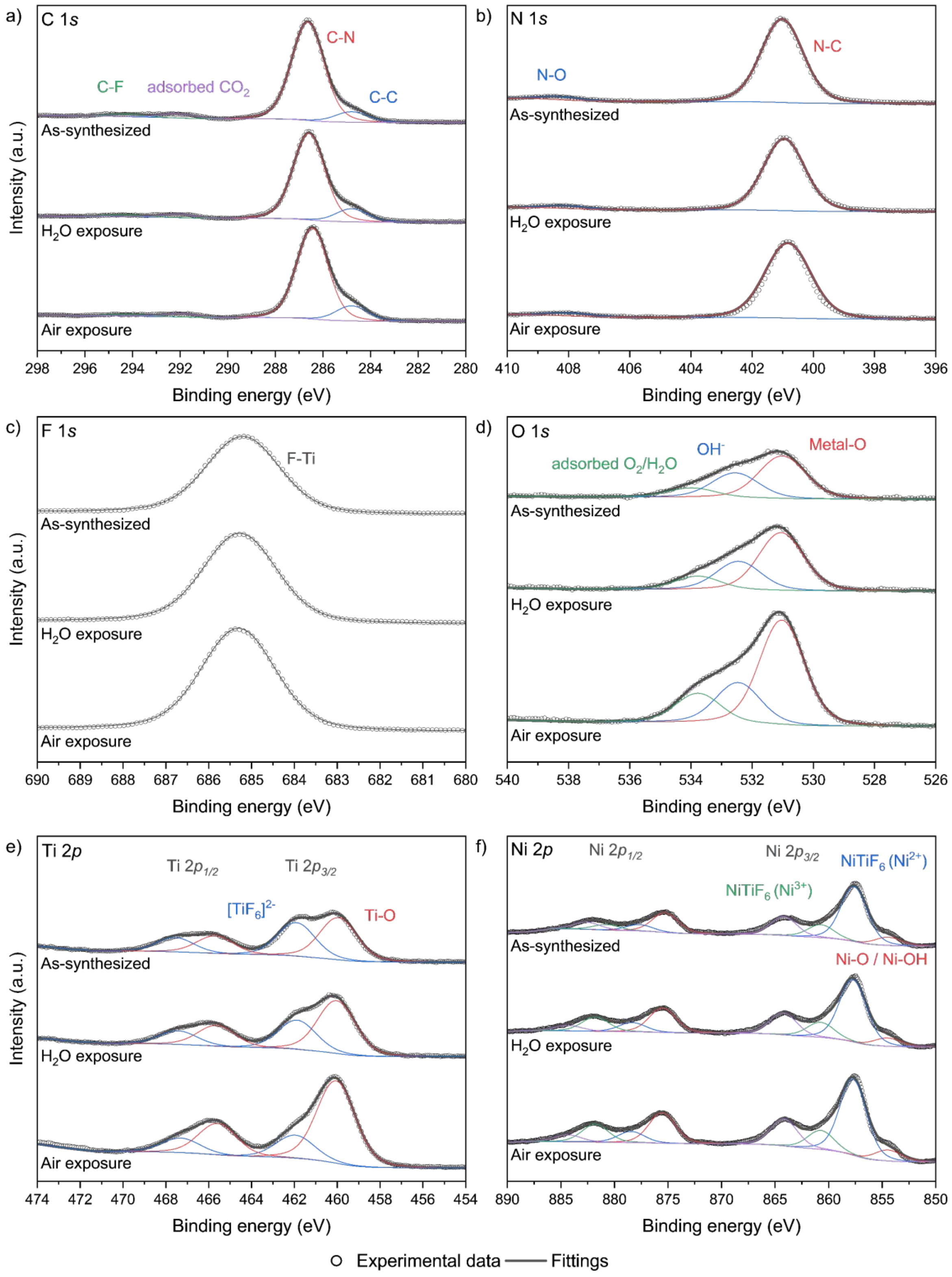


**Figure S19.** XPS data and peak fittings of the a) C 1*s*, b) N 1*s*, c) F 1*s*, d) O1*s*, e) Ti 2*p*, and f) Ni 2*p* core levels of TIFSIX-3-Ni samples (as synthesized, after $H_2O$ exposure, and after air exposure). Peaks were assigned based on information from the NIST XPS Database [5] and the sample's structure [6].

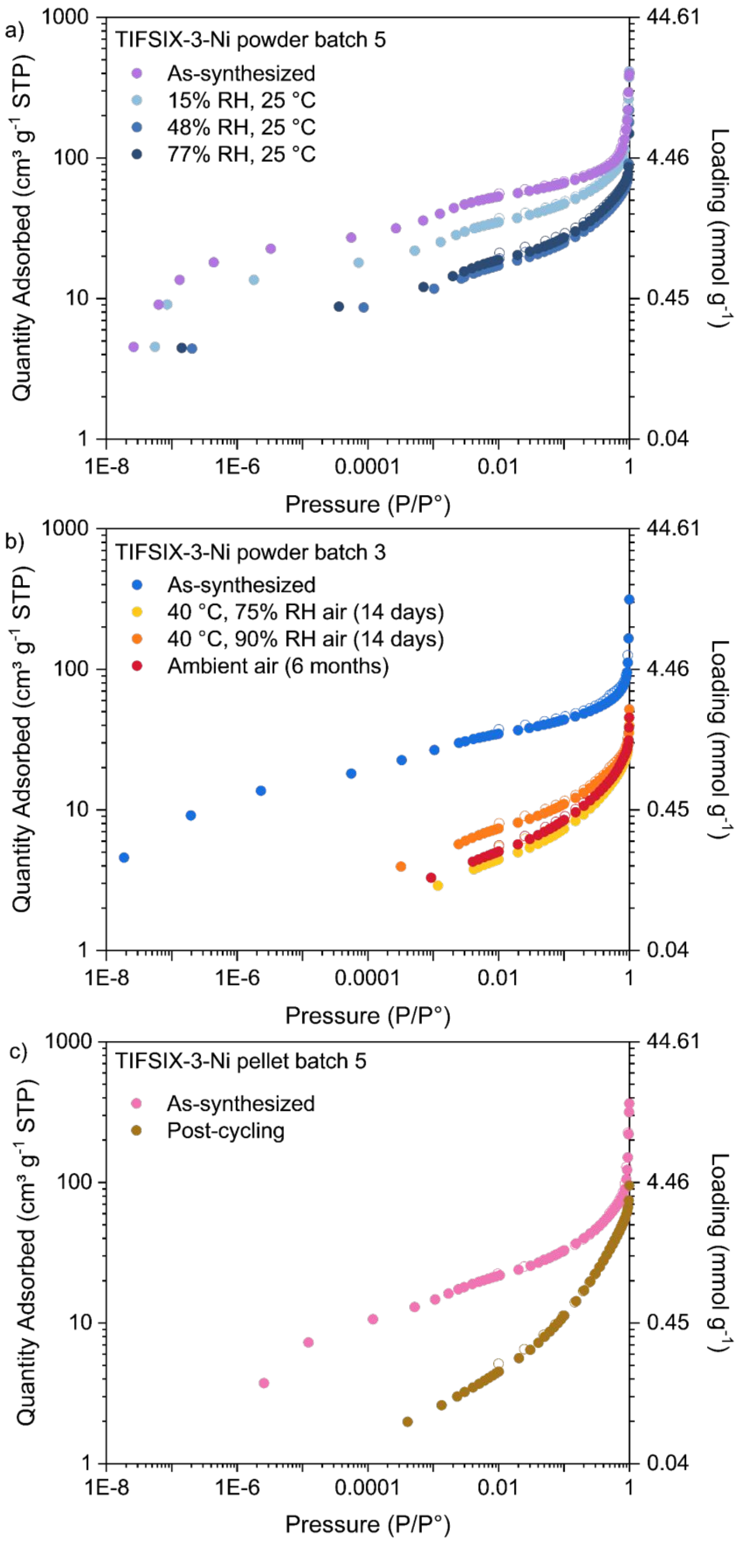


**Figure S20.** $N_2$ adsorption (filled symbols) and desorption (open symbols) isotherms at -196 °C with log-scale of pressure for a) batch 5 TIFSIX-3-Ni powder exposed to $H_2O$, b) batch 3 TIFSIX-3-Ni powder exposed to $H_2O$ and $O_2$, and c) batch 5 TIFSIX-3-Ni pellet before and after cycling.

**Table S11.** Images of the colors of batch 3 TIFSIX-3-Ni powder samples after exposure to different humidity conditions in air and subsequent characterizations.

| | | 4 | 5 | 6 |
|---|---|---|---|---|
| | Situation | Initial appearance | Appearance after ex-situ degas at 160 °C | Appearance after $CO_2$ 25 °C isotherm measurement |
| **E** | As synthesized (batch 3) | 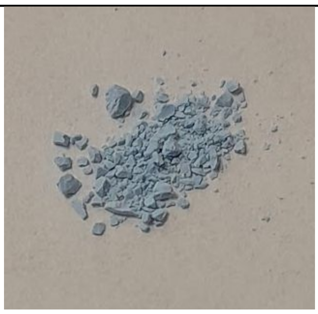 | Similar to Table S8 A2 | Similar to E4 |
| **F** | Exposure to air (40 °C, 75% RH, 14 days) | 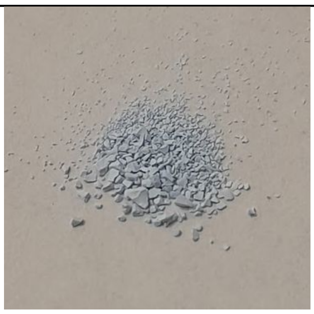 | Similar to Table S8 A2 | 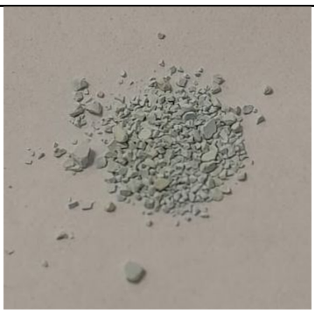 |
| **G** | Exposure to air (40 °C, 90% RH, 14 days) | 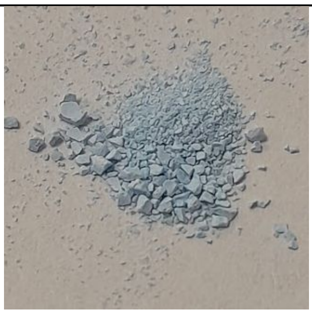 | Similar to Table S8 A2 | 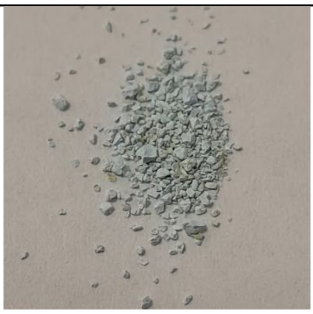 |
| **H** | Exposure to ambient air from the laboratory for 6 months | 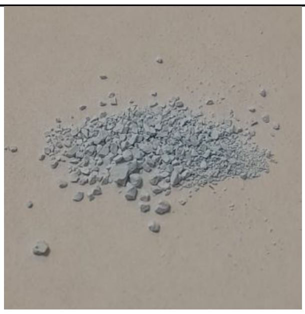 | Similar to Table S8 A2 | 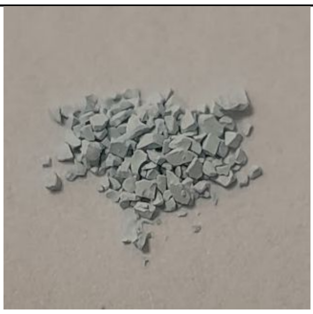 |

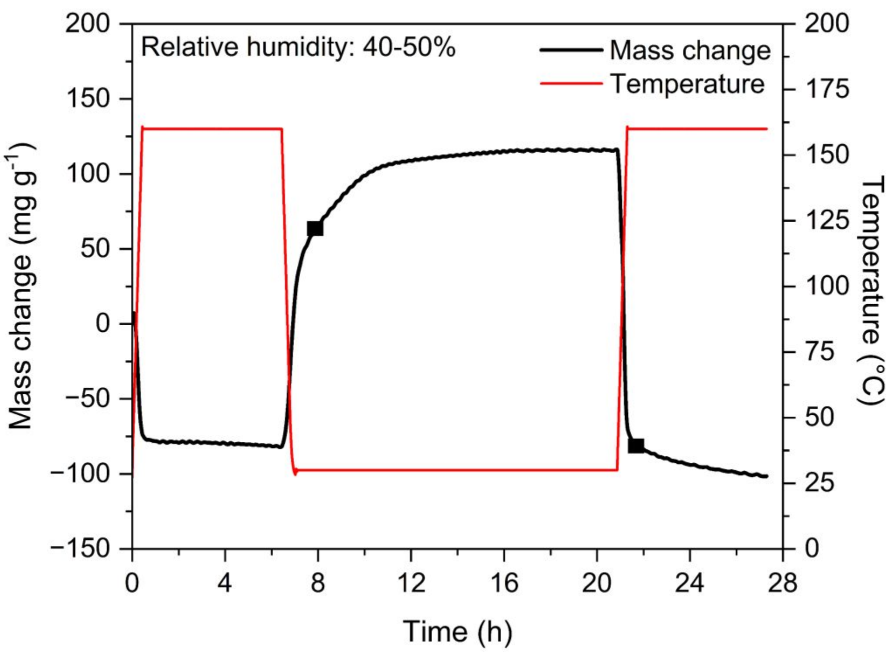


**Figure S21.** Mass change of batch 5 TIFSIX-3-Ni pellets over the course of a desorption-adsorption-desorption cycle under a flow of ambient air from the laboratory carried out using a thermogravimetric analyzer. Black squares indicate the cut-off times used for adsorption and desorption segments in the cyclic experiment.

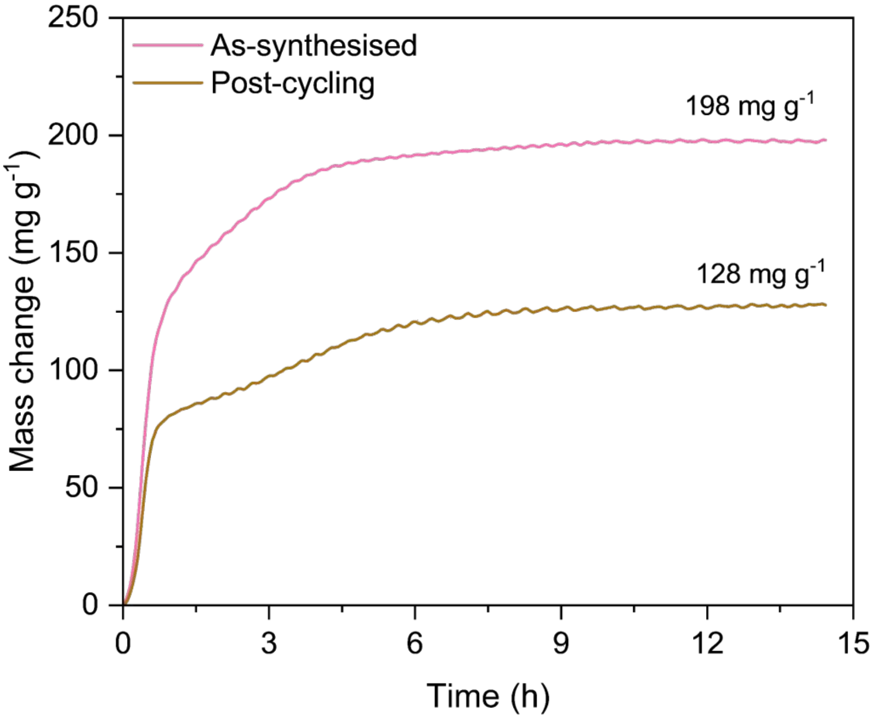


**Figure S22.** Comparison of the amount of gas adsorbed under a flow of ambient air by as-synthesized batch 5 TIFSIX-3-Ni pellets and TIFSIX-3-Ni pellets which have undergone 50 cycles of adsorption at 30 °C for 1 h and desorption at 160 °C for 15 min using a thermogravimetric analyzer.

## References

(1) NIST. *Aluminium Oxide*. 2023. https://webbook.nist.gov/cgi/cbook.cgi?ID=C1344281&Type=JANAFS&Plot=on (accessed 2024).

(2) *Cambridge Crystallographic Data Centre*. https://www.ccdc.cam.ac.uk/ (accessed 2024).

(3) Halasyamani, P.; Willis, M. J.; Stern, C. L.; Poeppelmeier, K. R. Crystal growth in aqueous hydrofluoric acid and (HF)x · pyridine solutions: syntheses and crystal structures of $[Ni(H_2O)_6]^{2+}[MF_6]^{2-}$ (M = Ti, Zr, Hf) and $Ni_3(py)_{12}F_6 \cdot 7H_2O$. *Inorganica Chimica Acta* **1995**, *240* (1-2), 109-115. DOI: 10.1016/0020-1693(95)04650-x.

(4) Kumar, A.; Hua, C.; Madden, D. G.; O'Nolan, D.; Chen, K. J.; Keane, L. J.; Perry, J. J.; Zaworotko, M. J. Hybrid ultramicroporous materials (HUMs) with enhanced stability and trace carbon capture performance. *Chem Commun (Camb)* **2017**, *53* (44), 5946-5949. DOI: 10.1039/c7cc02289a.

(5) Naumkin, A. V.; Kraut-Vass, A.; Gaarenstroom, S. W.; Powell, C. J.; Lee, A. Y. *NIST X-ray Photoelectron Spectroscopy Database*. https://srdata.nist.gov/xps/ (accessed 2024).

(6) Korochentsev, V. V.; Laptash, N. M. Intermetallic charge transfer in $MTiF_6{\cdot}6H_2O$ (M = Mn, Fe, Co, and Ni): A study by X-ray photoelectron spectroscopy. *Solid State Sciences* **2024**, *148* (107433), 1-8. DOI: 10.1016/j.solidstatesciences.2023.107433.
